# Using deep neural networks and LASSO regression to predict miRNA expression changes based on mRNA data

**DOI:** 10.3389/fbinf.2025.1566162

**Published:** 2025-07-08

**Authors:** Franz Leonard Böge, Helena U. Zacharias, Stefanie C. Becker, Klaus Jung

**Affiliations:** ^1^ Institute for Animal Genomics, University of Veterinary Medicine Hannover Foundation, Hannover, Germany; ^2^ Peter L. Reichertz Institute for Medical Informatics of TU Braunschweig and Hannover Medical School, Hannover Medical School, Hannover, Germany; ^3^ Institute of Parasitology, University of Veterinary Medicine Hannover Foundation, Hannover, Germany

**Keywords:** microRNA, artificial neural networks, LASSO regularization, West Nile virus, human immunodeficiency virus, multi-omics

## Abstract

**Introduction:**

Since the rise of molecular high-throughput technologies, many diseases are now studied on multiple omics layers in parallel. Understanding the interplay between microRNAs (miRNA) and their target mRNAs is important to understand the molecular level of diseases. While much public data from mRNA experiments are available for many diseases, few paired datasets with both miRNA and mRNA expression profiles are available. This study aimed to assess the possibility of predicting miRNA expression data based on mRNA expression data, serving as a proof of principle that such cross-omics predictions are feasible. Furthermore, current research relies on target databases where information about miRNA–target relationships is provided based on experimental and computational studies.

**Methods:**

To make use of publicly available mRNA profiles, we investigate the ability of artificial deep neural networks and linear least absolute shrinkage and selection operator (LASSO) regression to predict unknown miRNA expression profiles. We evaluate the approach using seven paired miRNA/mRNA expression datasets, four from studies on West Nile virus infection in mouse tissues and three from human immunodeficiency virus (HIV) infection in human tissues. We assessed the performance of each model first by within-data evaluations and second by cross-study evaluations. Furthermore, we investigated whether data augmentation or separate models for data from diseased and non-diseased samples can improve the prediction performance.

**Results:**

In general, most settings achieved strong correlations at the Level of individual samples. In some datasets and settings, correlations of log-fold changes and p-values from differential expression analysis (DEA) between true and predicted miRNA profiles can be observed. Correlation between log fold changes could also be seen in a cross-study evaluation for the HIV datasets. Data augmentation consistently improved performance in neural networks, while its impact on LASSO models was not significant.

**Discussion:**

Overall, cross-omics prediction of expression profiles appears possible, even with some correlations on the Level of the differential expression analysis.

## 1 Introduction

After microRNAs (miRNAs) were detected in 1993 (Lee et al., 1993), it was widely shown that these molecules play an essential role in the regulation of mRNA expression (Hammond, 2015). Much research on miRNAs was done in the context of cancer (Di Leva et al., 2014), but their role in neurodegenerative diseases (Nelson et al., 2008) and—to a lesser extent—during infections was also shown (Correia et al., 2017). miRNA biogenesis occurs in both the nucleus and cytoplasm. Initially, RNA polymerase II transcribes pri-miRNA (∼70 bp), which is processed by the Drosha-DGCR8 complex into precursor miRNA (pre-miRNA). Exported to the cytoplasm, pre-miRNAs are further processed by Dicer into mature double-stranded miRNAs (∼22 nt). The functional strand then integrates into the RNA-induced silencing complex (RISC), enabling gene regulation. miRNAs exert control by binding target mRNAs via partial complementarity, primarily through the seed sequence (2–8 nt from the 5′-end), which pairs with the 3′-UTR of target mRNAs, ensuring regulatory specificity (O’Brian et al., 2018). Due to the requirement for only partial complementarity, a single miRNA can regulate multiple mRNAs, ranging from a few to several hundred. Additionally, target sets often overlap, allowing miRNAs to coordinate complex gene regulatory networks.

While an increasing number of studies on mRNA expression have been conducted, either using microarray or RNA-seq technology (Lowe et al., 2017), the number of studies on miRNA expression is much lower. Furthermore, even fewer studies collect parallel data of both mRNA and miRNA expression from the same samples, and parallel data can sometimes be incomplete. Although some approaches have been presented to impute missing data in multi-omics settings (Dong et al., 2019; Song et al., 2020), these approaches make little use of the special relationship between miRNAs and mRNA targets. Because there are masses of mRNA expression data available at public databases such as NCBI Gene Expression Omnibus (GEO) (Clough and Barrett, 2016) or ArrayExpress (Kolesnikov et al., 2015), it would be of great use if these data could be employed to infer by which miRNAs the individual mRNAs were targeted, for example, during a disease. Currently, this association is mostly made by relying on information from target databases that connect miRNAs and target mRNAs, where the information was either experimentally or computationally validated (Ru et al., 2014). We recently presented a simple approach to treat this problem by running gene-set tests on mRNA target sets (Böge et al., 2024). For significant target sets, we concluded that their corresponding miRNA was altered by the disease. Overall, we found a significant but rather small correlation between the p-values for the target set and the p-values from differential miRNA testing.

Here, we present and evaluate an approach to directly infer miRNA expression profiles from available mRNA expression profiles using deep learning neural networks and linear least absolute shrinkage and selection operator (LASSO) regression. LASSO regression is one of the most popular regularization techniques for analyzing high-dimensional datasets in the p > n scenario (Tibshirani, 1996). It has been extensively used for the development of sparse biomarker signatures from routine patients (Zacharias et al., 2022), as well as (multi-)omics data (Zacharias et al., 2019; Ma et al., 2022; Onwuka et al., 2024), and likewise for multi-omics data integration (Sun et al., 2023). So far, deep neural networks and other machine learning models trained on mRNA expression profiles were mainly fitted for the purpose of predicting class labels of samples—with the first attempts reaching back 20 years (Van’t Veer et al., 2002; Zhu and Hastie, 2004)—but not expression profiles from another omics layer. In addition, in the context of integrating multi-omics data, most approaches aimed at making joint use of different but parallel omics data to classify sample labels (Kang et al., 2022). Specifically, Fuchs et al. (2013) combined parallel miRNA and mRNA data for predicting sample labels. Doing predictions on the miRNA level using mRNA expression data has also been evaluated by Nielsen and Pedersen (2021) and Olgun et al. (2022) but not yet with deep neural networks or LASSO regression. The approach by Nielsen and Pederson is similar to our approach presented in Böge et al. (2024): they used motif enrichment analyses on the mRNA level to predict a score of miRNA activity and evaluated the performance by correlating the predicted score against the true miRNA expression levels. Olgun et al., instead, trained miRNA-individual models using gradient boosting combined with decision trees and single-cell RNA-seq (scRNA-seq) data as input and finally correlated fold changes derived from the predicted miRNA expression data against fold changes derived from the true data.

In order to build on our previous work, we use expression data from infection research: four datasets from studies on West Nile virus (WNV) infections and three datasets on human immunodeficiency virus (HIV) infections. MicroRNAs (miRNAs) play a key role in WNV infection, replication, and neuropathogenesis. In WNV strain 385-99-infected HEK293 and SK-N-MC cells as well as mouse central nervous system tissues, Hs_154 was upregulated, reducing viral replication and modulating apoptosis by targeting CTCF and ECOP/VOPP1 (Smith et al., 2012). Similarly, in WNV strain KUN-infected HEK293 cells, miR-532-5p exhibited antiviral effects by suppressing SESTD1 and TAB3, essential for viral replication (Slonchak et al., 2016). In WNV-infected mouse brains, 139 miRNAs were altered, with miR-196a, miR-202-3p, miR-449c, and miR-125a-3p regulating inflammation, immune-cell trafficking, and apoptosis (Kumar and Nerurkar, 2014).

Even more information is available for HIV infection and miRNAs (Chinniah et al., 2022). In brief, miRNAs influence HIV infection by modulating viral replication, host immunity, and disease progression. HIV exploits cellular miRNAs to enhance its replication while also using viral-encoded miRNAs to regulate both viral and host mRNAs, potentially controlling its replication and latency. Specifically, miRNA expression in CD4^+^ T cells correlates with HIV viral load. When viral load decreases, miR-146a, miR-29a/b, miR-155, and Let-7c are upregulated, reducing CXCR4, gag, nef, LEDGF, and p21, while miR-148a is downregulated, increasing HLA-C expression. Conversely, when viral load increases, the opposite occurs: miR-146a, miR-29a/b, miR-155, and Let-7c are downregulated, promoting viral replication, while miR-148a is upregulated, suppressing HLA-C. These miRNA shifts play a key role in HIV progression and immune response. Additionally, miRNAs may affect HIV susceptibility in monocytes and macrophages.

The seven parallel datasets used for training and evaluation, as well as the machine learning methods and the methods for evaluation and DEA, are described in the following chapter. In the results chapter, we present the performance of within-data evaluation and of evaluation with independent data. Finally, we discuss the benefits and limitations of our approach.

## 2 Methods

In this section, we first describe the data used for training and evaluation of the machine learning models, as well as how they were selected from the public databases. Next, we specify the modeling process for the deep neural networks and the LASSO approach. All data analyses were performed using the R programming environment (V 4.4.2, www.r-project.org) and bioinformatics packages from the Bioconductor repository (www.bioconductor.org).

### 2.1 Identification of parallel miRNA/mRNA expression data for training and evaluation

Data were sourced from the NCBI Gene Expression Omnibus (GEO) (https://www.ncbi.nlm.nih.gov/geo/), a public database for high-throughput functional genomics data, particularly data from transcriptome studies. We searched for studies related to zoonotic diseases in humans and mice as this continues our previous work on transcriptomic changes under zoonotic diseases. First, we searched for data on ArrayExpress (https://www.ebi.ac.uk/biostudies/arrayexpress), a database for high-throughput transcriptomics data. We searched for all studies related to mouse and WNV (search term: {“West Nile” AND mouse}), yielding 19 search results from December 2023. Of these, three were removed due to insufficient data or a flawed experimental setup. From the remaining studies, we selected those studies containing both miRNA and mRNA transcriptomic data. Among the 16 studies, five, all parts of a super-series, contained both miRNA and mRNA transcriptomic data. Both types of data were not available on ArrayExpress for all five studies, but the data were available on the GEO database. All five were microarray datasets. Dataset GSE68380-GSE68381 was excluded due to low quality of the data, leaving four WNV parallel datasets (Table 1). Second, we selected three HIV parallel datasets from GEO, two of which were microarray data and one of which was RNA-seq data (Table 2).

**TABLE 1:** List of parallel WNV mRNA–miRNA expression datasets used to evaluate the aim of predicting miRNA expression from mRNA expression data.

Dataset no.	Databank ID	Organism	Tissue	Time after infection	Sample size (control + WNV)	References	Omics level
1	GSE77193	C57BL/6	Cortex	4 days	5 + 5	Feng et al. (2021)	mRNA
1	GSE77161	C57BL/6	Cortex	4 days	5 + 5	Feng et al. (2021)	miRNA
2	GSE77192	C57Bl/6	Cerebellum	4 days	5 + 5	Feng et al. (2021)	mRNA
2	GSE77160	C57Bl/6	Cerebellum	4 days	5 + 5	Feng et al. (2021)	miRNA
3	GSE78888	C57BL/6	Popliteal lymph node	1 day	3 + 5	Feng et al. (2021)	mRNA
3	GSE78887	C57BL/6	Popliteal lymph node	2 days	3 + 5	Feng et al. (2021)	miRNA
4	GSE67473	C57Bl/6	Cortical neuron	24 h	6 + 6	Feng et al. (2021)	mRNA
4	GSE67474	C57Bl/6	Cortical neuron	12 h	5 + 6	Feng et al. (2021)	miRNA

The table provides the GEO, database ID, organism, type of tissue, time after infection, sample sizes in the control and diseased group, literature reference, and omics level. For dataset no. 5, mRNA and miRNA data were provided under the same accession number.

**TABLE 2:** List of parallel HIV mRNA–miRNA expression datasets used to evaluate the aim of predicting miRNA expression from mRNA expression data.

Dataset no.	Databank ID	Organism	Tissue	Time after infection	Sample size (control + HIV)	References	Omics level
5	GSE76246	Human	Whole blood	-	7 + 50		mRNA/miRNA
6	GSE247191	Human	CD4+T cells	2 days	3 + 6	Bellini et al. (2024)	mRNA
6	GSE247194	Human	CD4+T cells	2 days	3 + 6	Bellini et al. (2024)	miRNA
7	GSE140713	Human	Whole blood	-	8 + 50	Shen et al. (2021)	mRNA
7	GSE140650	Human	Whole blood	-	8 + 50	Shen et al. (2021)	miRNA

The table provides the GEO, database ID, organism, type of tissue, time after infection, sample sizes in the control and diseased group, literature reference, and the omics level. For dataset no. 5, mRNA and miRNA data were provided under the same accession number.

### 2.2 Data preprocessing and augmentation

Expression data of all WNV datasets (No. 1–4) were labeled as normalized on the ArrayExpression database. According to the original publication by Feng et al. (2021), quantile normalization of the R package “limma” was used for these datasets. In fact, a check by boxplots showed the same distribution for all samples of a dataset and, therefore, we continued without additional normalization. Two of the HIV datasets, No. 5 and No. 7, were also available in a normalized form. As there is not yet a publication for dataset No. 5, the normalization method is unknown, but boxplots again suggested that the data were quantile normalized. According to the original publication of dataset No. 7 by Shen et al. (2021), the authors also employed quantile normalization, however, using the R package “AgiMicroRna.” Only for the non-normalized dataset No. 6 were expression data profiled by RNA-seq. We applied the voom transformation (Law et al., 2014) combined with quantile normalization, implemented in the R package “limma,” separately for mRNA and miRNA data.

In addition to normalization and for better comparability of datasets, all input and output data were compressed to have expression levels between 0 and 1. Because we initially observed that prediction can shift miRNA data compared to the true data, the compression also had a positive effect on suppressing the shift and making the true and predicted data more comparable.

For the data augmentation, we added Gaussian noise to the expression data from both the control and diseased groups as proposed by Kircher et al. (2022). Noise was added to each sample’s feature label using a small standard deviation of 0.01. The process was repeated until at least 100 samples were generated.

### 2.3 Deep neural networks and linear LASSO regression models

#### 2.3.1 Architecture of the deep neural network

All neural networks were trained using the R package “keras” (V 2.15), an implementation of the keras deep learning library (Gulli and Pal, 2017). After probing different networks with different numbers and types of layers, numbers of neurons, dropout rates, and activation functions, we continued with the most promising setting as follows. Each neural network trained in this study was structured with four hidden dense layers. All started with an input layer of as many neurons as there were relevant mRNA transcripts, reflecting the input feature count. With the different datasets, the size of the input layer varied between 14,977 and 60,623 neurons. Specifically for the WNV datasets, the number of mRNA predictors is 39,429 features, and the number of miRNAs to be predicted is 3,105. In the case of the HIV datasets, dataset No. 5 consists of 14,977 mRNA features and 499 mRNAs, dataset No. 6 involves 60,623 mRNAs and 3,017 miRNAs, and finally, dataset No. 7 has 41,091 mRNAs and 2,027 miRNAs.

For the number of knots per layer, we chose a hierarchical reduction in layer size, with the layers containing 1,024, 512, 256, and 128 neurons, respectively. Each dense layer employs the rectified linear unit (ReLU) activation function. To accelerate the learning rate of the model, we applied batch normalization after each dense layer. To avoid overfitting of the model, each dense layer is regularized with L2 penalties. In addition, we included a dropout rate of 0.4. The final output layer is configured to reflect the output feature count, that is, the number of miRNA features to be predicted, and uses a linear activation function.

As the loss function, we chose mean squared error, and for the performance metric, we chose mean absolute error. To improve training efficiency and convergence, we used the Adam optimizer with a learning rate of 0.001 to update the model’s parameters. We further introduced adaptive learning rate adjustments, which reduce the learning rate by a factor of 0.5 upon performance plateaus. Each neural network is trained over a maximum of 150 epochs, with a batch size of 32 and a validation split of 20% to ensure generalization.

#### 2.3.2 Linear LASSO regression models

For training linear LASSO regression models, the R package “glmnet” (V 4.1.8, Hastie et al., 2020) was used. Here, an individual model was trained for each miRNA. The whole data matrix of mRNA expression levels was used as predictor variables. Due to the small sample size of our datasets, we did not optimize λ by cross-validation. Instead, we fitted models for a decreasing set of λ-values that is automatically generated by the glmnet function. For evaluation of the models, we first used those based on the minimal λ-value, which results in a small mean squared error (MSE) while bearing a strong risk for overfitting. Second, we chose models trained with the 10th largest of the λ-values. Those models have a larger MSE but are more generalizable. We also checked models with even larger λ-values, but these did not result in good correlation between true and predicted miRNA expression profiles. The idea of the evaluation was not to find the optimal model but to check two oppositional scenarios.

### 2.4 Differential expression analysis

Differential expression analysis (DEA) on the true and on the predicted miRNA expression profiles was performed using the R package “limma” (Ritchie et al., 2015) (V 3.6). Specifically, we used these models to derive log2 fold changes (log2FC) and raw p-values for each miRNA. We omitted adjustments for multiple testing as we were only interested in studying the correlations between logFCs or p-values from true and predicted data.

### 2.5 Evaluation of prediction performance

**FIGURE 1:**
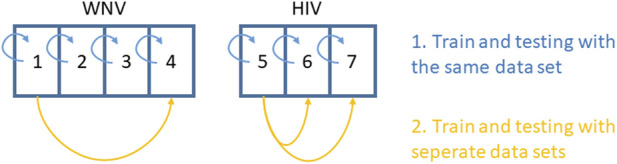
Setup for within-data and cross-study evaluation of model performance.

For all seven datasets, we first made a within-dataset evaluation to check whether the models are in principle able to produce expression profiles with a similar distribution to the original data. That is, we used the same data for evaluation as for training (Figure 1). Due to the small sample sizes of all datasets, we also avoided within-data cross-validation procedures, being aware that we will most likely overestimate the performance of the models. Therefore, we also performed cross-study evaluations with selected datasets. As the WNV mouse data were collected from different tissues, we only used the two datasets obtained from the cortex and cortical neurons (datasets no. 1 and 4), respectively. From the HIV setting, we chose datasets no. 5 for training and either no. 6 or no. 7 for cross-study validation.

In each evaluation, we studied the sample-wise correlations between predicted and true miRNA expression profiles. Next, we correlated log2FCs and p-values as obtained from the DEA of the predicted and true expression profiles. All correlation analyses were performed using Pearson’s correlation coefficient R.

## 3 Results

In this section, we will first present the results obtained from using the neural networks and then the results from training the data with linear LASSO regression. For both types of models, we describe first the results of the within-dataset evaluation and then the results of the evaluation with independent datasets.

### 3.1 Performance of the deep neural networks

#### 3.1.1 Within-dataset evaluation

We used the four WNV and three HIV parallel miRNA/mRNA datasets to analyze the capabilities of the neural networks to predict miRNA expression profiles based on mRNA expression profiles. For all seven datasets, we evaluated the performance of the networks under four different settings, based on whether the datasets were augmented or not and whether models were trained jointly or separately for the infected and non-infected groups. That is, we first investigated whether an insufficient quantity of training data could be overcome through augmenting the training data with artificially generated samples. Second, we investigated whether training separate neural networks for healthy and diseased groups would improve the prediction performance.

As detailed in the Methods section, we tested all WNV and HIV datasets after training the neural network with a given dataset using the same mRNA data to predict the related miRNA expression profile. We then analyzed the performance on the level of the predicted data themselves and also on the level of the analyses with these data. In particular, we first checked the correlation between predicted and true miRNA expression levels for each individual sample. Next, we performed DEA with both the predicted and the true miRNA expression profiles and subsequently investigated the correlation of the fold changes and p-values derived from the true and predicted data.

**FIGURE 2:**
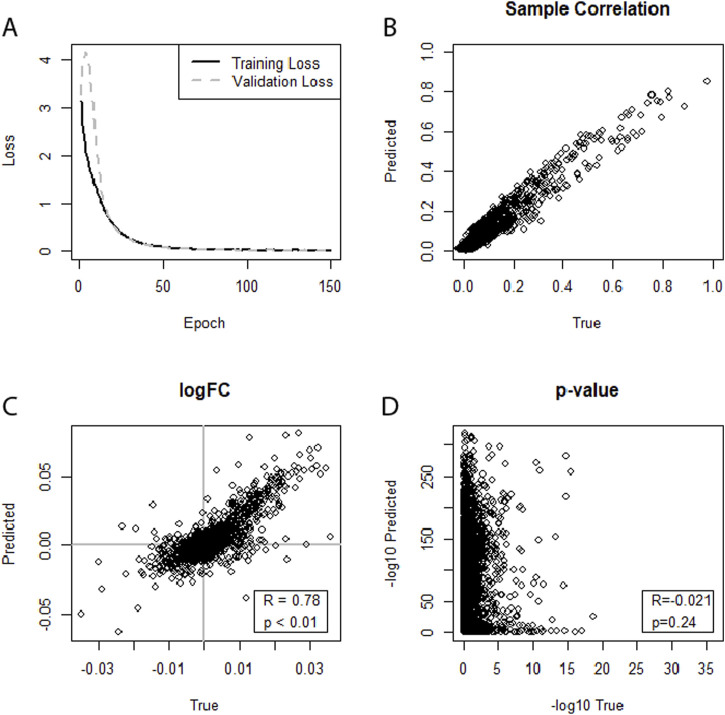
**(A–D)** Illustrating the performance of separate neural networks trained for each condition (healthy, sick) in predicting an miRNA transcriptome based on an augmented mRNA transcriptome. Dataset no. 4 (WNV, GSE67473-GSE67474) was used for training and testing. **(A)** Training loss and validation loss of the neural network. **(B)** Depicts good miRNA expression-level correlation between predicted and true miRNA values for the first sample of the dataset. **(C)** Shows a moderate correlation for log2FCs, **(D)** Indicates no correlation between the predicted and true DEA p-values.

With all four WNV datasets, no. 1 (Supplementary Figures S1–S4), no. 2 (Supplementary Figures S5–S8), no. 3 (Supplementary Figures S1–S4), and no. 4 (Figure 2), significant correlations of the miRNA expression levels of predicted versus true datasets were observed at the level of individual samples. However, further correlation between log2FCs or p-values of the DEA was only observed for datasets no. 3 and no. 4. For dataset no. 3 (WNV, GSE78887-GSE78888), the best performance was achieved when using augmented data and training on an individual network for the two study groups. With these settings, a correlation of R = 0.37 between log2FCs was reached. Dataset no. 4 (WNV, GSE67473-GSE67474) yielded the best result. When training separate neural networks for both conditions and using augmented data, the model’s ability to predict miRNA profiles that correlate with real miRNA profiles reached R = 0.78 (Figure 2).

**FIGURE 3:**
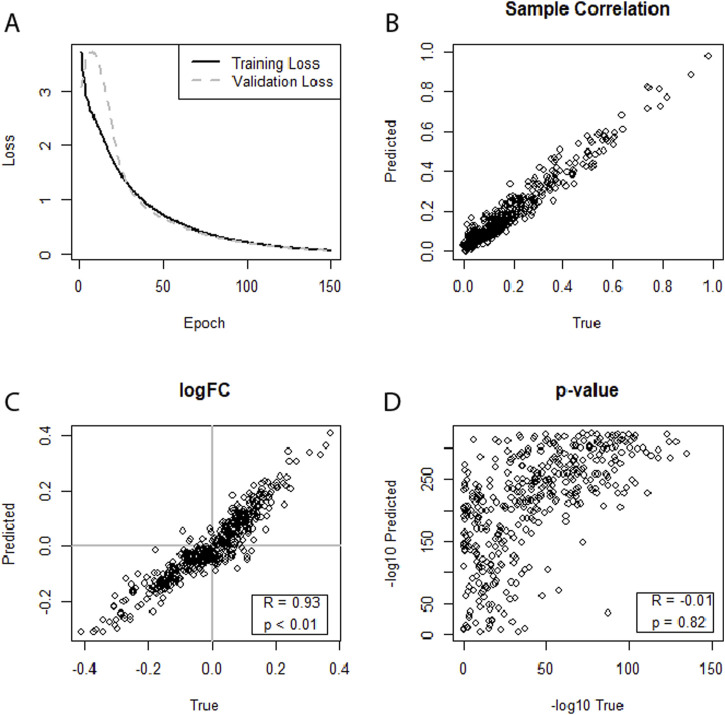
**(A–D)** Illustrating the performance of separate neural networks trained for each condition (healthy, sick) in predicting an miRNA transcriptome based on an augmented mRNA transcriptome. Dataset no. 5 (HIV, GSE76246) was used for training and testing. **(A)** Training loss and validation loss of the neural network. **(B)** Depicts strong miRNA expression-level correlation between predicted and true miRNA values for the first sample of the dataset. **(C)** Shows a strong correlation for log2FCs. **(D)** Indicates a weak-to-moderate correlation between predicted and true differential expression p-values.

In the case of HIV, all three datasets, no. 5 (GSE76246), no. 6 (GSE247191-GSE247194), and no. 7 (GSE140713-GSE140650), using separate neural networks on the augmented dataset no. 5 (GSE76246) yielded strong correlations of the miRNA expression levels for individual samples. In addition, all HIV datasets also showed strong correlations between the log2FCs, with dataset no. 5 achieving a correlation between the log2FCs of R = 0.93 (Figure 3) when using separate neural networks and augmented data. The graph also hinted at a possible extension of this trend to the p-values. However, this was not substantiated as the correlation of the p-values was R = −0.019. Overall, predictions in the HIV datasets performed well, with dataset no. 6 (GSE247191-GSE247194) achieving correlations of log2FCs of up to R = 0.57 (SF24) and dataset No.7 (GSE140713-GSE140650) achieving correlations of R = 0.75 (SF28).

After examining the overall performance of neural networks across all seven datasets (Supplementary Figures S1–S12, S14–29), we then checked whether data augmentation or training separate neural networks for each condition could boost the performance. For data augmentation, we compared the average correlation between log2FCs of predicted and true miRNA transcriptomes when the training data were augmented and when not across all settings of the seven datasets. We found that augmentation on average increased Pearson’s correlation coefficient R by 0.3 (minimum: −0.3, maximum: 1.12), indicating that data augmentation increases performance.

While investigating whether separate neural networks for each condition would enhance performance, we found that training separate neural networks on not-augmented data showed a decrease in correlation across all seven datasets. This is potentially due to decreasing the already limited number of samples for training. Therefore, we considered only augmented data when investigating whether separate neural networks could enhance the performance. We found that training separate neural networks on augmented data rather than one single neural network on average decreased the correlation of log2FCs between predicted and true miRNA transcriptomes by 0.19 (minimum: −0.83, maximum: 0.64). This indicates that, on average, separate neural networks have a negative effect on the performance. However, in some instances, like dataset no. 4, having trained two separate neural networks drastically improved results (R = 0.64).

For dataset no. 3 (WNV, GSE78887-GSE78888), we further evaluated the strategy of filtering out the 20% lowest-expressed mRNAs and miRNAs because these features can be supposed to be less affected by the disease and therefore not informative for the neural network. We also assumed that this strategy could enhance the prediction accuracy by reducing noise introduced by the minimally expressed features. In the instance of the dataset no. 3, filtering improved performance from R = 0.37 to R = 0.55. However, the same approach did not yield consistent improvements in other datasets tested, such as the HIV datasets no. 5–7, which all showed a decrease in correlation for log2FCs. This suggests that the effectiveness of expression-based filtering may depend on dataset-specific characteristics rather than being a universally applicable strategy.

#### 3.1.2 Cross-dataset predictions

With the neural network architecture optimized and validated on single datasets, we proceeded to test the neural network performance on independent datasets for validation (Supplementary Figures S30–S37). For WNV, we encountered the challenge of having four datasets, each corresponding to a different tissue type. To maintain biological relevance, we trained the model using cortex tissue data (dataset no. 1: GSE77161-GSE77193) and tested it on cortical neuron data (dataset no. 4: GSE67473-GSE67474). However, the independent validation failed to produce meaningful predictions for these datasets. There was no correlation observed between the individual samples regarding predicted and true miRNA expression levels. In addition to dataset no. 4, dataset no. 3 also performed well in the “within data” validation; however, no datasets with closely related tissue types were available. Even when ignoring biological relevance and training with dataset no. 3 (GSE78887-GSE78888), while testing with any other WNV dataset, predictions also did not yield any correlation, even on the sample level.

**FIGURE 4:**
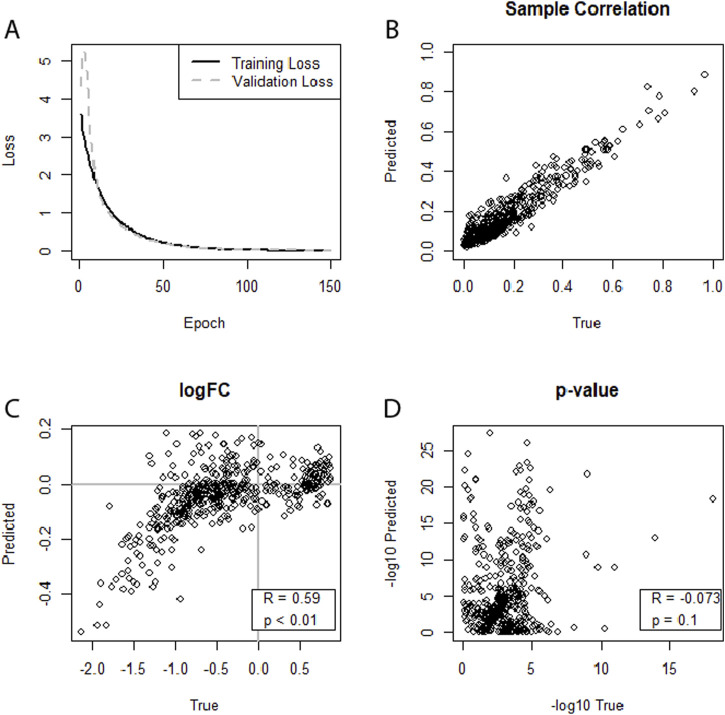
**(A–D)** Illustrating the performance of a neural network on predicting an miRNA transcriptome based on an mRNA transcriptome. Augmented dataset no. 5 (GSE76246) was used for training, and dataset no. 7 (GSE140713-GSE140650) was used for testing. **(A)** Training loss and validation loss of the neural network. **(B)** Depicts good miRNA expression-level correlation between predicted and true miRNA values for the first sample of the dataset. **(C)** Shows correlation for log2FCs. **(D)** Indicates no correlation between predicted and true differential expression p-values.

For HIV, we had three datasets, no. 5 (GSE76246), no. 6 (GSE247191-GSE247194), and no. 7 (GSE140713-GSE140650), where no. 5 and 7 are based on whole blood samples and no. 6 is based on CD4+T cells. In addition, no. 5 and no. 7 were Agilent microarray data, while no. 6 was RNA-seq data acquired through an Illumina NovaSeq 6000. We tested two different dataset combinations for training and validation: dataset no. 5 for training combined with either of the other two datasets for cross-study validation. Both approaches produced strong correlations at the sample level. However, when using no. 7 for cross-study validation, a strong correlation between the log2FCs of the DEA of R = 0.59 can be observed, demonstrating the model’s ability to generalize effectively within datasets of the same tissue type (Figure 4).

### 3.2 Comparison with linear LASSO regression models

**FIGURE 5:**
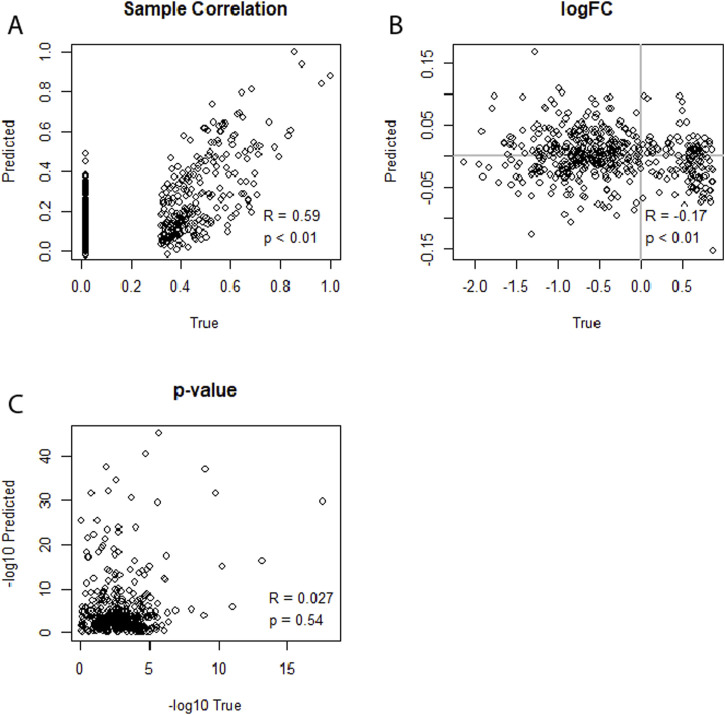
**(A–C)** Illustrating the performance of separate LASSO regression models for each condition (healthy, sick) in predicting an miRNA transcriptome based on an mRNA transcriptome. Dataset no. 5 (GSE76246) was used for training, and dataset no. 7 (GSE140713-GSE140650) was used for testing. The 10th highest lambda was used. **(A)** Depicts strong miRNA expression-level correlation between predicted and true miRNA values for the first sample of the dataset. **(B)** Shows no correlation for log2FCs. **(C)** Indicates no correlation between the predicted and true differential expression p-values.

To compare the performance of the neural network, we compared its predictive capability to that of linear LASSO regression models (Supplementary Figures S38–S49). In contrast to the neural networks, we did not fit combined models; that is, we used separate LASSO models for each condition (healthy, infected). For these analyses, the same datasets as for the cross-data neural network evaluations were used: dataset no. 1 and no. 4 for WNV, and no. 5, no. 6, and no. 7 for HIV. We tested whether the strength of the model regularization or the augmentation of the data had a positive effect on the performance of the LASSO models. We found that linear LASSO models had the ability to achieve sample-level correlations of up to R = 0.78 between predicted and actual miRNA expression profiles, even when using non-augmented data (Supplementary Figure S49; Figure 5). However, upon examining the results of the DEA, the LASSO model predictions showed no meaningful correlations for the log2FCs or p-values of the DEA. We further found that the performance was irrespective of the λ regularization parameter used (minimal or 10th highest λ) or whether the data was augmented or not. This pattern was consistent across both the WNV and HIV datasets, highlighting the limitation of linear LASSO regression models in capturing the nuances of differential expression patterns in the framework of predicted expression profiles, despite their strong performance in sample-level correlations. When using dataset no. 5 (GSE76246) for training and no. 7 (GSE140713-GSE140650) for testing, we further investigated whether a voom transformation of the data could increase the model’s performance. However, this approach did not improve the model’s capability to predict miRNA expression profiles to the extent that it could get similar results to the actual miRNA expression data.

## 4 Discussion

The rise of molecular high-throughput technologies enabled the study of diseases on multiple omics layers in parallel. This also allowed studying intricate biological mechanisms, for example, the interplay between individual omics layers, such as between the proteome and transcriptome (Ragno et al., 2001; Wu et al., 2020; Barbato et al., 2009).

Another layer of major interest is the miRNA transcriptome. It has been shown that miRNAs are involved in nearly all pathologies, from viral infection to cancer and heart disease. This makes miRNA a prime target for future therapeutics. However, there are two challenges limiting practical applications of miRNAs: their instability, making them very tedious to work with, and their intricate and subtle interactions that are difficult to entangle with current methods (Chen et al., 2015).

Most existing studies on microRNA aim to identify their target mRNAs, either by experimental studies (Thomson et al., 2011) or by computational methods (Watanabe et al., 2007). In contrast, little effort has been made concerning the reverse approach, predicting miRNA profiles from mRNA data. In this work, we demonstrate as a proof of principle that with deep neural networks and LASSO regression, it is possible to predict the composition of one omics layer based on another one.

It is important to note that in the case of cross-study validation, we mostly observed correlations on the individual sample level. Strong correlations between predicted and true miRNA transcriptome extending to the DEA could only be observed with dataset no. 5 for training and then testing it on no. 7. These two datasets were comparably sized, were produced by the same study group, and used the same microarray platform. This indicates that the best performance can be achieved by datasets with similar data size and sufficient size. It seems plausible that a more generalizable neural network could be achieved with substantially more data available for training.

When comparing the neural network approach with linear LASSO regression models, two key observations stand out. First, linear LASSO regression models are capable of capturing sample-level correlations; however, they struggle to predict miRNA expression profiles whose DEA correlates with the ones of the actual miRNA profiles. Second, in contrast to neural networks, LASSO regression models did not benefit from data augmentation. In fact, the linear character of LASSO regression might be causative for their limited performance. Non-linear machine learning methods, such as random forest and other tree-based approaches, might be a good alternative for further efforts to make cross-omics predictions. In fact, Olgun et al. (2022), who used gradient boosting combined with decision trees, reached very good performance when comparing fold changes from true and predicted miRNA profiles. The performance they obtained is difficult to compare with the correlations we obtained because they used scRNA-seq data with much more training data available than in our examples. Random forest has already been used to integrate multi-omics data from transcriptomics and proteomics (Acharjee et al., 2016). From our findings, however, we consider that the architecture of artificial neural networks might be better suited for predicting the complex nature of expression matrices.

Thus, for future studies, a significant challenge will be obtaining a sufficiently large sample size of high-quality training data. We encountered a substantial difference in performance between the individual datasets that we tested. Specifically, lower performance was observed with datasets no. 1 and no. 2. However, we found that augmenting the training data can mitigate some of the challenges introduced by the small datasets. Meanwhile, further methods for data augmentation of transcriptomics data have been published, which we will consider for our continued research (Janakarajan et al., 2025; Kalimumbalo et al., 2025). Data augmentation has not only been demonstrated to substantially improve the prediction performance of neural networks in general, but it is also known to prevent models from overfitting.

We also investigated the impact of excluding the 20% lowest-expressed miRNAs and mRNAs from the analysis to reduce potential noise. This approach yielded only situational benefits. Similarly, attempting to boost the performance by training separate neural networks for each individual condition (healthy, diseased) yielded only situational improvement.

It is important to emphasize that this approach has the potential to be applied to any omics layer of interest. For example, predicting protein expression profiles based on mRNA data would also be very helpful to understand the cross-omics interplay. In addition, this suggests that by analyzing only a single omics layer, one may infer information about all other omics layers of a sample and thus the state of the sample in its entirety.

Another application of interest that this approach provides is that even if the model fails to reveal insights of the differential expression directly, its performance can be a valuable indicator of the correlation that exists between two omics layers.

Finally, machine learning models for cross-omics predictions can be of great value for biological interpretation. For better interpretability of the deep neural networks shown in Figures 2, 3, we additionally analyzed the weights obtained for the first layer of these networks. In particular, we determined the average weight with which the normalized mRNA expression levels are forwarded to the second layer. We then studied what proportion of transcription factors (TF) was among the top 5% of mRNA with the largest average weight. For that purpose, we used the set of known human TFs, as reported on https://humantfs.ccbr.utoronto.ca/index.php (Lambert et al., 2018).

For the networks trained with the WNV data, 2.7% (diseased cases) and 2.8% (control cases) of the top 5% predictors were classified as TFs, compared to 3.9% TFs in the whole mRNA dataset. For the networks trained with the HIV data, 6.1% TFs were found in the total mRNA dataset, and 8.6% (diseased cases) and 8.6% (control cases) were among the top 5% predictors.

Thus, in both scenarios, the proportion of TFs among the top 5% predictors did not strongly deviate from the proportion in the whole dataset. Nevertheless, identifying these TFs can be an important contribution to the interpretability of the models, as it is well known that there is an overall strong interaction between miRNAs and transcription factors during gene regulation (Chen and Rajewsky, 2007).

## Data Availability

Publicly available datasets were analyzed in this study. The expression data of the HIV study can be publicly retrieved from the NCBI Gene Expression Omnibus database (https://www.ncbi.nlm.nih.gov/geo/) using the accession numbers GSE76246, GSE247191, GSE247194, GSE140713, and GSE140650. The expression data of the WNV studies can be retrieved using the accession numbers GSE77193, GSE77161, GSE77192, GSE77160, GSE78888, GSE78887, GSE67473, and GSE67474.
