# Supplementary material for: Using deep neural networks and LASSO regression to predict miRNA expression changes based on mRNA data
Source: Front Bioinform. 2025 Jul 8;5:1566162. doi: 10.3389/fbinf.2025.1566162 (PMC12279838; doi:10.3389/fbinf.2025.1566162)
Supplement: Supplementary file 1 [file Presentation1.pptx]

## Slide 1
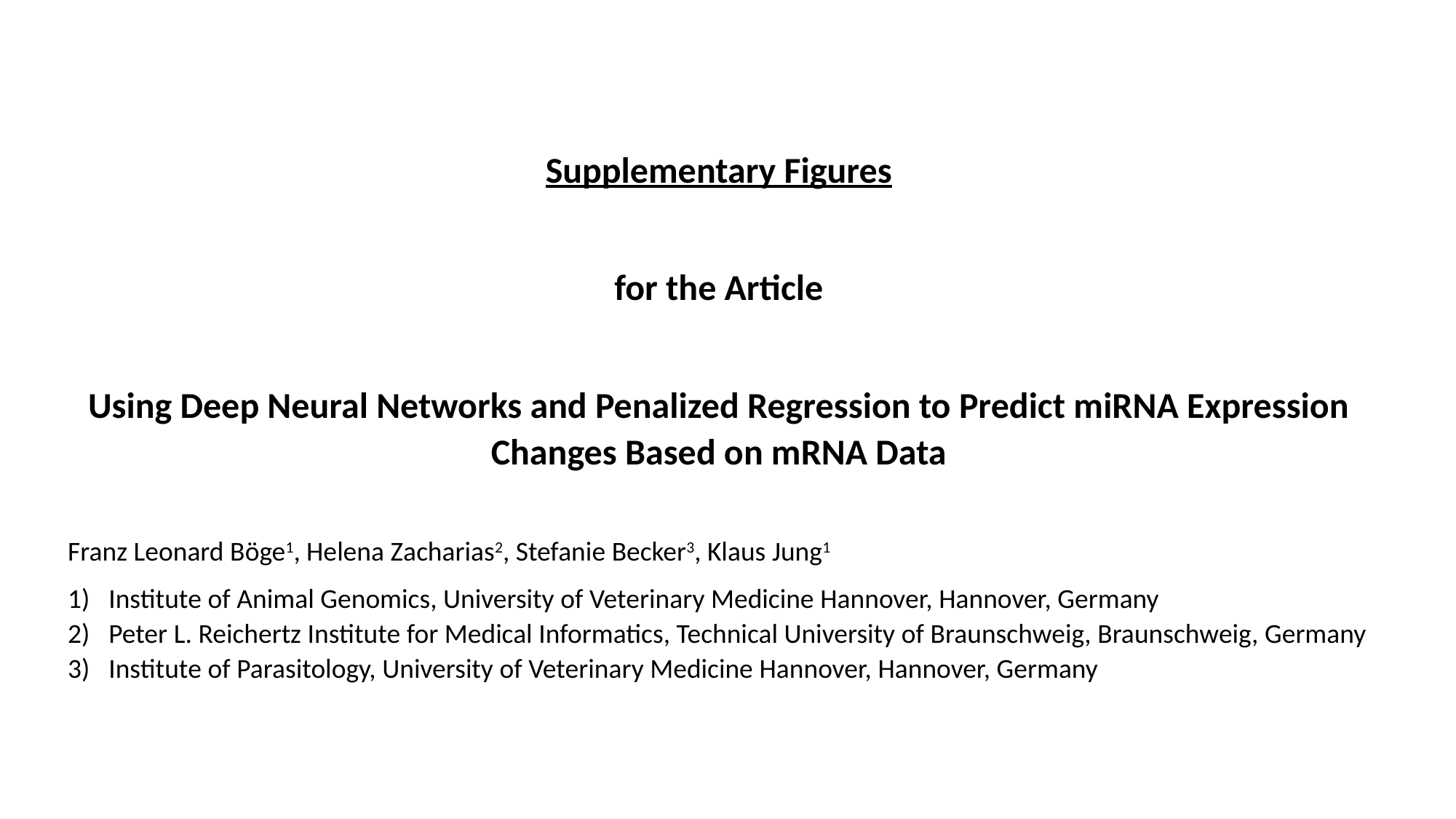

Supplementary Figures
for the Article
Using Deep Neural Networks and Penalized Regression to Predict miRNA Expression Changes Based on mRNA Data
Franz Leonard Böge1, Helena Zacharias2, Stefanie Becker3, Klaus Jung1
Institute of Animal Genomics, University of Veterinary Medicine Hannover, Hannover, Germany
Peter L. Reichertz Institute for Medical Informatics, Technical University of Braunschweig, Braunschweig, Germany
Institute of Parasitology, University of Veterinary Medicine Hannover, Hannover, Germany

## Slide 2
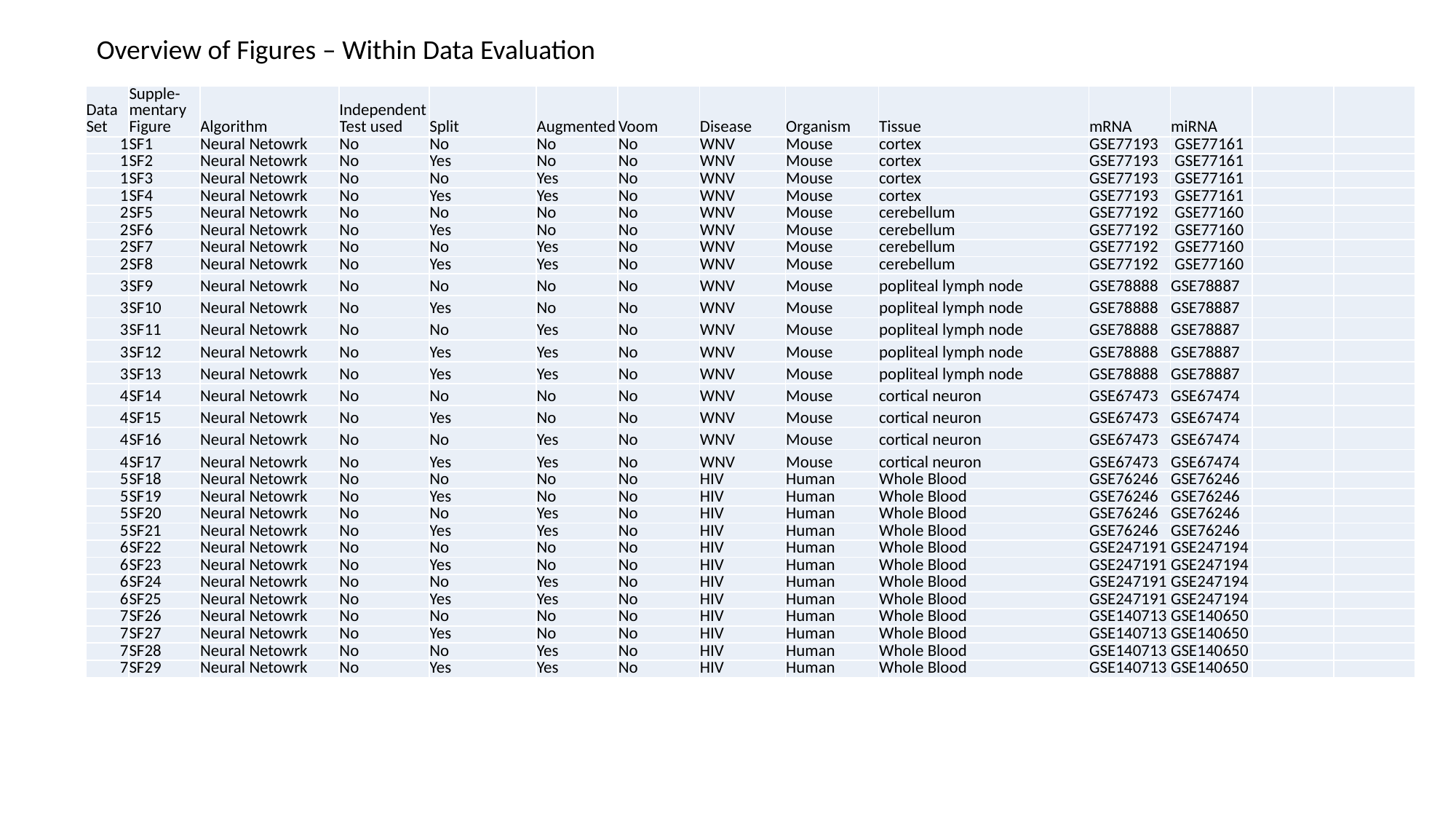

Overview of Figures – Within Data Evaluation
| Data Set | Supple-mentary Figure | Algorithm | Independent Test used | Split | Augmented | Voom | Disease | Organism | Tissue | mRNA | miRNA | | |
| --- | --- | --- | --- | --- | --- | --- | --- | --- | --- | --- | --- | --- | --- |
| 1 | SF1 | Neural Netowrk | No | No | No | No | WNV | Mouse | cortex | GSE77193 | GSE77161 | | |
| 1 | SF2 | Neural Netowrk | No | Yes | No | No | WNV | Mouse | cortex | GSE77193 | GSE77161 | | |
| 1 | SF3 | Neural Netowrk | No | No | Yes | No | WNV | Mouse | cortex | GSE77193 | GSE77161 | | |
| 1 | SF4 | Neural Netowrk | No | Yes | Yes | No | WNV | Mouse | cortex | GSE77193 | GSE77161 | | |
| 2 | SF5 | Neural Netowrk | No | No | No | No | WNV | Mouse | cerebellum | GSE77192 | GSE77160 | | |
| 2 | SF6 | Neural Netowrk | No | Yes | No | No | WNV | Mouse | cerebellum | GSE77192 | GSE77160 | | |
| 2 | SF7 | Neural Netowrk | No | No | Yes | No | WNV | Mouse | cerebellum | GSE77192 | GSE77160 | | |
| 2 | SF8 | Neural Netowrk | No | Yes | Yes | No | WNV | Mouse | cerebellum | GSE77192 | GSE77160 | | |
| 3 | SF9 | Neural Netowrk | No | No | No | No | WNV | Mouse | popliteal lymph node | GSE78888 | GSE78887 | | |
| 3 | SF10 | Neural Netowrk | No | Yes | No | No | WNV | Mouse | popliteal lymph node | GSE78888 | GSE78887 | | |
| 3 | SF11 | Neural Netowrk | No | No | Yes | No | WNV | Mouse | popliteal lymph node | GSE78888 | GSE78887 | | |
| 3 | SF12 | Neural Netowrk | No | Yes | Yes | No | WNV | Mouse | popliteal lymph node | GSE78888 | GSE78887 | | |
| 3 | SF13 | Neural Netowrk | No | Yes | Yes | No | WNV | Mouse | popliteal lymph node | GSE78888 | GSE78887 | | |
| 4 | SF14 | Neural Netowrk | No | No | No | No | WNV | Mouse | cortical neuron | GSE67473 | GSE67474 | | |
| 4 | SF15 | Neural Netowrk | No | Yes | No | No | WNV | Mouse | cortical neuron | GSE67473 | GSE67474 | | |
| 4 | SF16 | Neural Netowrk | No | No | Yes | No | WNV | Mouse | cortical neuron | GSE67473 | GSE67474 | | |
| 4 | SF17 | Neural Netowrk | No | Yes | Yes | No | WNV | Mouse | cortical neuron | GSE67473 | GSE67474 | | |
| 5 | SF18 | Neural Netowrk | No | No | No | No | HIV | Human | Whole Blood | GSE76246 | GSE76246 | | |
| 5 | SF19 | Neural Netowrk | No | Yes | No | No | HIV | Human | Whole Blood | GSE76246 | GSE76246 | | |
| 5 | SF20 | Neural Netowrk | No | No | Yes | No | HIV | Human | Whole Blood | GSE76246 | GSE76246 | | |
| 5 | SF21 | Neural Netowrk | No | Yes | Yes | No | HIV | Human | Whole Blood | GSE76246 | GSE76246 | | |
| 6 | SF22 | Neural Netowrk | No | No | No | No | HIV | Human | Whole Blood | GSE247191 | GSE247194 | | |
| 6 | SF23 | Neural Netowrk | No | Yes | No | No | HIV | Human | Whole Blood | GSE247191 | GSE247194 | | |
| 6 | SF24 | Neural Netowrk | No | No | Yes | No | HIV | Human | Whole Blood | GSE247191 | GSE247194 | | |
| 6 | SF25 | Neural Netowrk | No | Yes | Yes | No | HIV | Human | Whole Blood | GSE247191 | GSE247194 | | |
| 7 | SF26 | Neural Netowrk | No | No | No | No | HIV | Human | Whole Blood | GSE140713 | GSE140650 | | |
| 7 | SF27 | Neural Netowrk | No | Yes | No | No | HIV | Human | Whole Blood | GSE140713 | GSE140650 | | |
| 7 | SF28 | Neural Netowrk | No | No | Yes | No | HIV | Human | Whole Blood | GSE140713 | GSE140650 | | |
| 7 | SF29 | Neural Netowrk | No | Yes | Yes | No | HIV | Human | Whole Blood | GSE140713 | GSE140650 | | |

## Slide 3
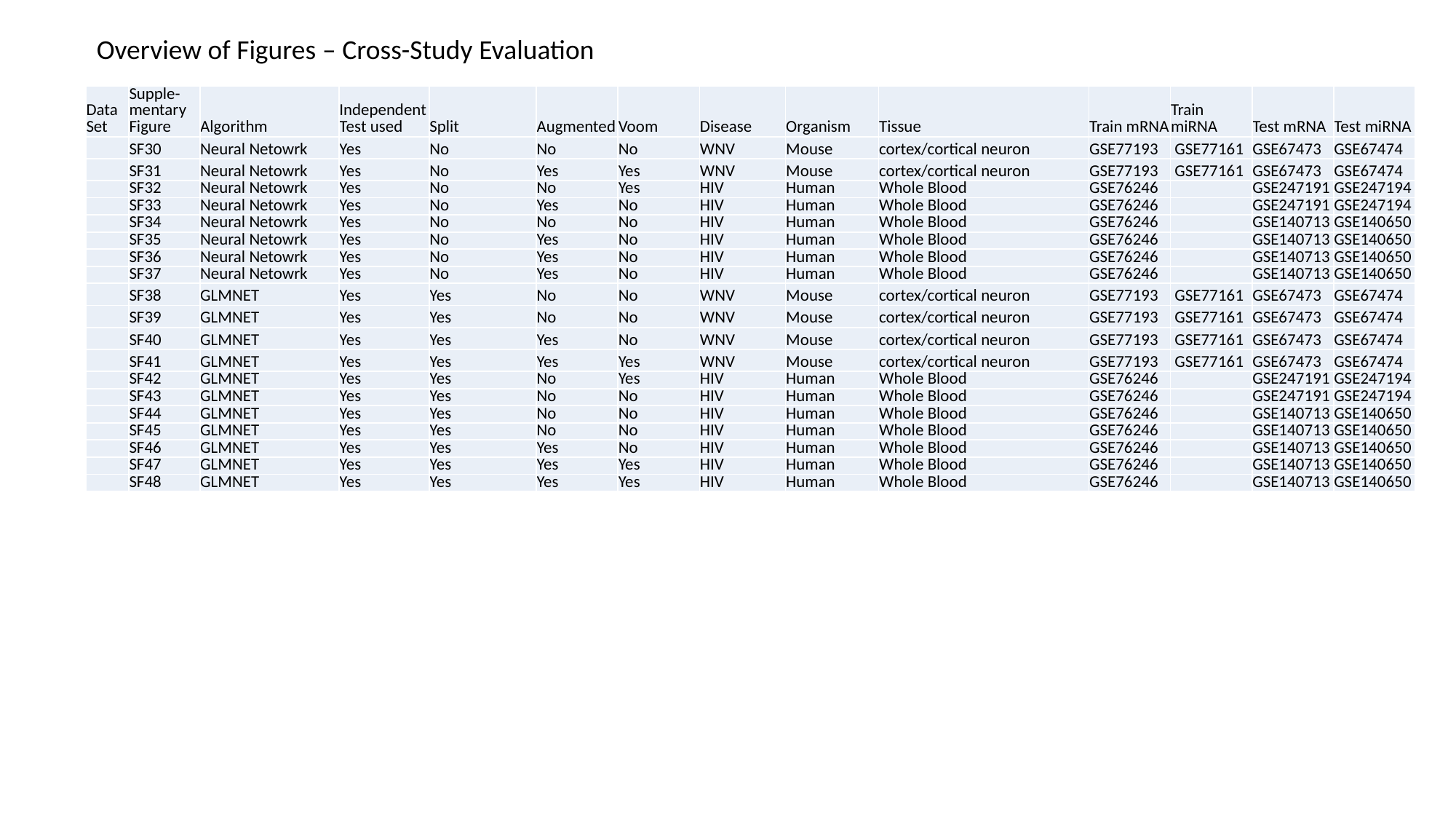

Overview of Figures – Cross-Study Evaluation
| Data Set | Supple-mentary Figure | Algorithm | Independent Test used | Split | Augmented | Voom | Disease | Organism | Tissue | Train mRNA | Train miRNA | Test mRNA | Test miRNA |
| --- | --- | --- | --- | --- | --- | --- | --- | --- | --- | --- | --- | --- | --- |
| | SF30 | Neural Netowrk | Yes | No | No | No | WNV | Mouse | cortex/cortical neuron | GSE77193 | GSE77161 | GSE67473 | GSE67474 |
| | SF31 | Neural Netowrk | Yes | No | Yes | Yes | WNV | Mouse | cortex/cortical neuron | GSE77193 | GSE77161 | GSE67473 | GSE67474 |
| | SF32 | Neural Netowrk | Yes | No | No | Yes | HIV | Human | Whole Blood | GSE76246 | | GSE247191 | GSE247194 |
| | SF33 | Neural Netowrk | Yes | No | Yes | No | HIV | Human | Whole Blood | GSE76246 | | GSE247191 | GSE247194 |
| | SF34 | Neural Netowrk | Yes | No | No | No | HIV | Human | Whole Blood | GSE76246 | | GSE140713 | GSE140650 |
| | SF35 | Neural Netowrk | Yes | No | Yes | No | HIV | Human | Whole Blood | GSE76246 | | GSE140713 | GSE140650 |
| | SF36 | Neural Netowrk | Yes | No | Yes | No | HIV | Human | Whole Blood | GSE76246 | | GSE140713 | GSE140650 |
| | SF37 | Neural Netowrk | Yes | No | Yes | No | HIV | Human | Whole Blood | GSE76246 | | GSE140713 | GSE140650 |
| | SF38 | GLMNET | Yes | Yes | No | No | WNV | Mouse | cortex/cortical neuron | GSE77193 | GSE77161 | GSE67473 | GSE67474 |
| | SF39 | GLMNET | Yes | Yes | No | No | WNV | Mouse | cortex/cortical neuron | GSE77193 | GSE77161 | GSE67473 | GSE67474 |
| | SF40 | GLMNET | Yes | Yes | Yes | No | WNV | Mouse | cortex/cortical neuron | GSE77193 | GSE77161 | GSE67473 | GSE67474 |
| | SF41 | GLMNET | Yes | Yes | Yes | Yes | WNV | Mouse | cortex/cortical neuron | GSE77193 | GSE77161 | GSE67473 | GSE67474 |
| | SF42 | GLMNET | Yes | Yes | No | Yes | HIV | Human | Whole Blood | GSE76246 | | GSE247191 | GSE247194 |
| | SF43 | GLMNET | Yes | Yes | No | No | HIV | Human | Whole Blood | GSE76246 | | GSE247191 | GSE247194 |
| | SF44 | GLMNET | Yes | Yes | No | No | HIV | Human | Whole Blood | GSE76246 | | GSE140713 | GSE140650 |
| | SF45 | GLMNET | Yes | Yes | No | No | HIV | Human | Whole Blood | GSE76246 | | GSE140713 | GSE140650 |
| | SF46 | GLMNET | Yes | Yes | Yes | No | HIV | Human | Whole Blood | GSE76246 | | GSE140713 | GSE140650 |
| | SF47 | GLMNET | Yes | Yes | Yes | Yes | HIV | Human | Whole Blood | GSE76246 | | GSE140713 | GSE140650 |
| | SF48 | GLMNET | Yes | Yes | Yes | Yes | HIV | Human | Whole Blood | GSE76246 | | GSE140713 | GSE140650 |

## Slide 4
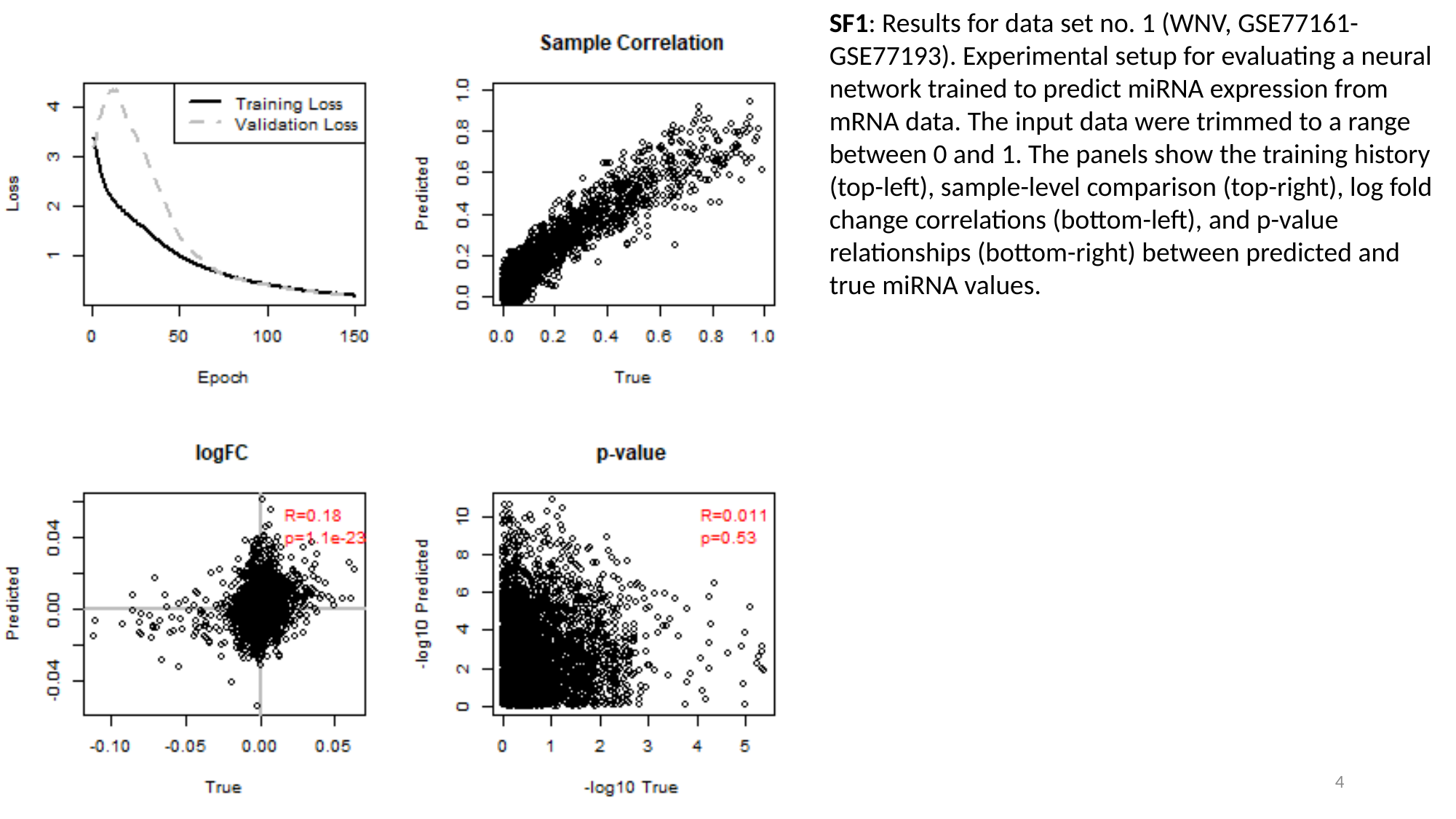

SF1: Results for data set no. 1 (WNV, GSE77161-GSE77193). Experimental setup for evaluating a neural network trained to predict miRNA expression from mRNA data. The input data were trimmed to a range between 0 and 1. The panels show the training history (top-left), sample-level comparison (top-right), log fold change correlations (bottom-left), and p-value relationships (bottom-right) between predicted and true miRNA values.
4

## Slide 5
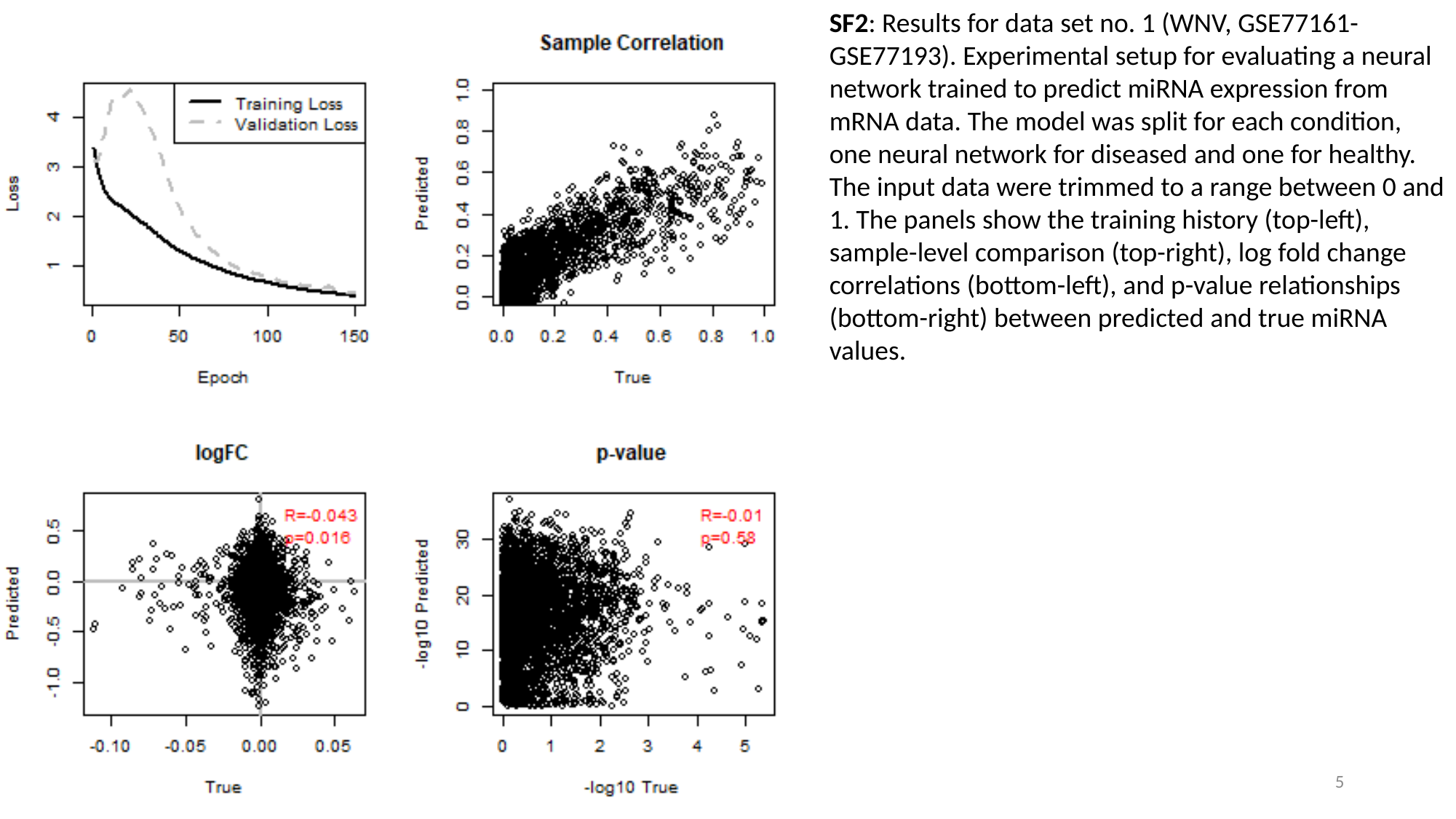

SF2: Results for data set no. 1 (WNV, GSE77161-GSE77193). Experimental setup for evaluating a neural network trained to predict miRNA expression from mRNA data. The model was split for each condition, one neural network for diseased and one for healthy. The input data were trimmed to a range between 0 and 1. The panels show the training history (top-left), sample-level comparison (top-right), log fold change correlations (bottom-left), and p-value relationships (bottom-right) between predicted and true miRNA values.
5

## Slide 6
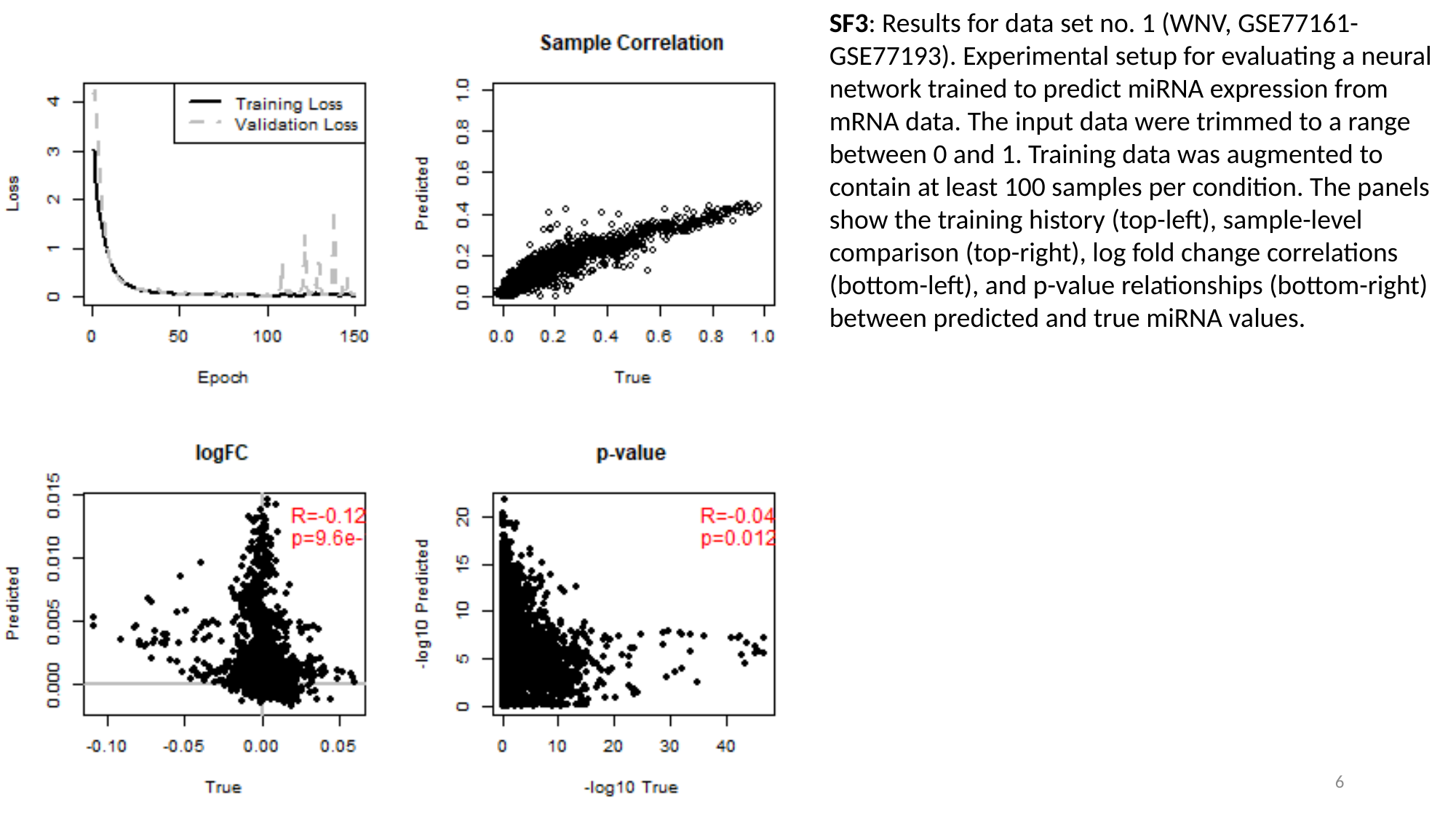

SF3: Results for data set no. 1 (WNV, GSE77161-GSE77193). Experimental setup for evaluating a neural network trained to predict miRNA expression from mRNA data. The input data were trimmed to a range between 0 and 1. Training data was augmented to contain at least 100 samples per condition. The panels show the training history (top-left), sample-level comparison (top-right), log fold change correlations (bottom-left), and p-value relationships (bottom-right) between predicted and true miRNA values.
6

## Slide 7
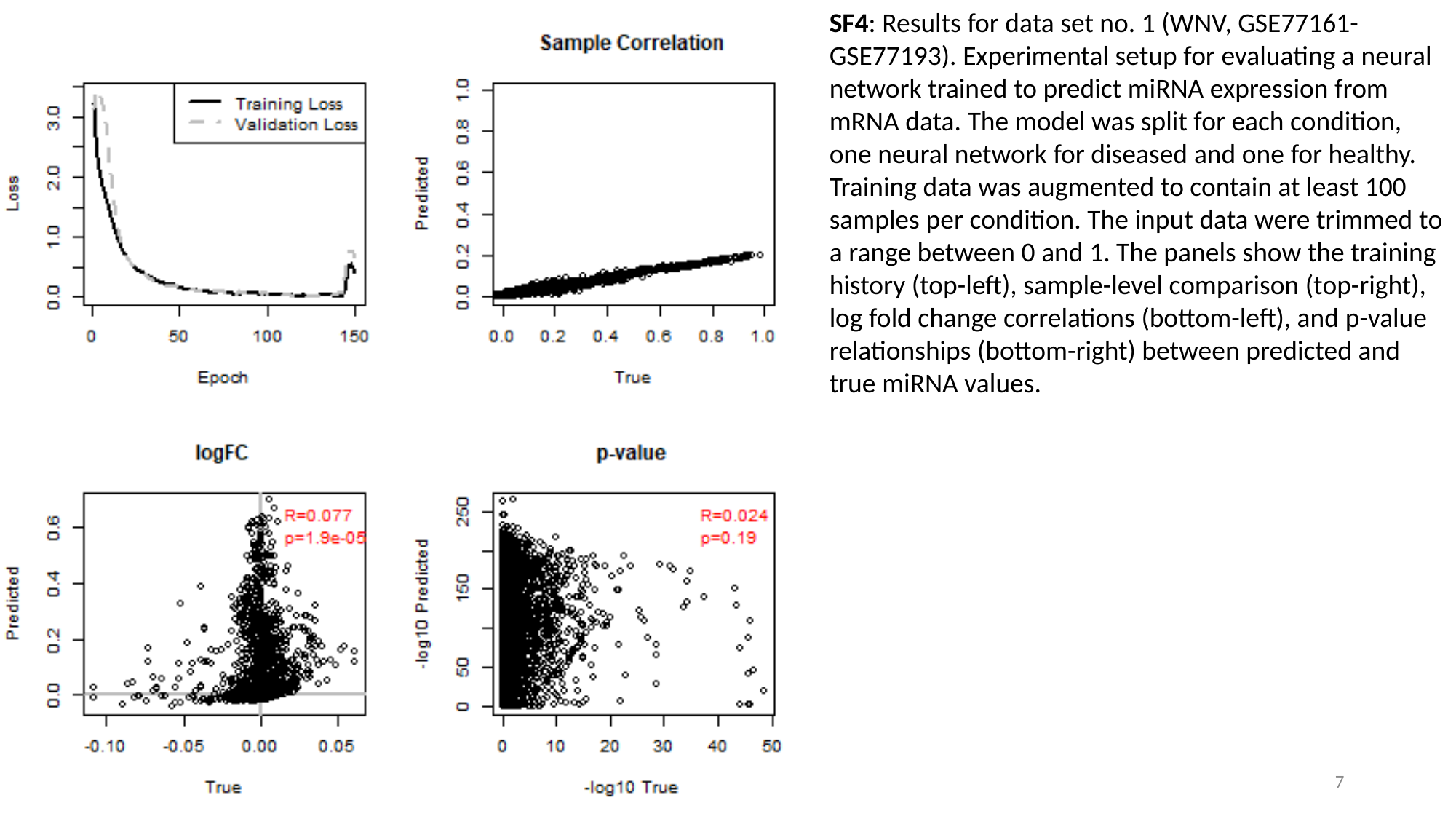

SF4: Results for data set no. 1 (WNV, GSE77161-GSE77193). Experimental setup for evaluating a neural network trained to predict miRNA expression from mRNA data. The model was split for each condition, one neural network for diseased and one for healthy. Training data was augmented to contain at least 100 samples per condition. The input data were trimmed to a range between 0 and 1. The panels show the training history (top-left), sample-level comparison (top-right), log fold change correlations (bottom-left), and p-value relationships (bottom-right) between predicted and true miRNA values.
7

## Slide 8
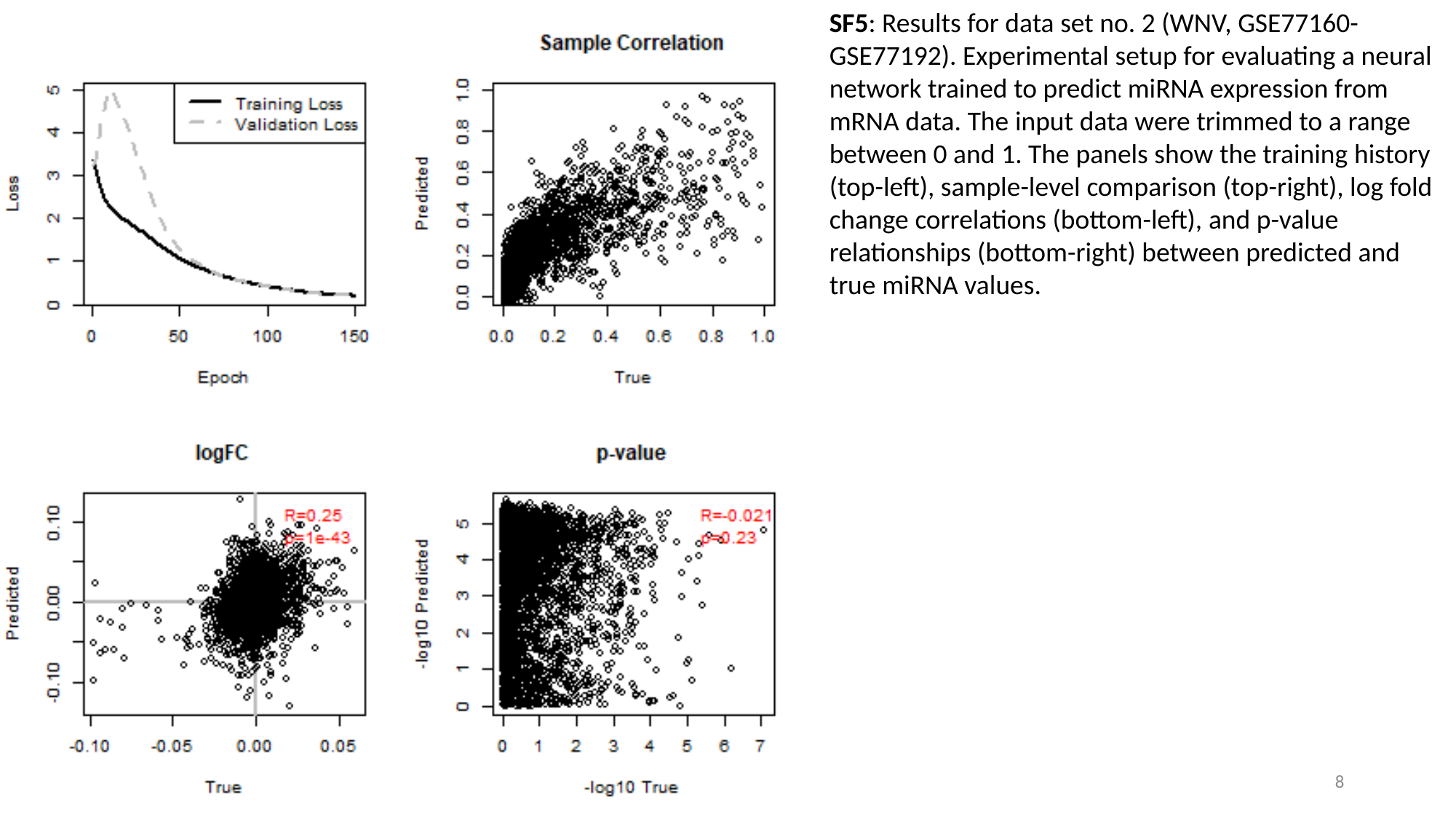

SF5: Results for data set no. 2 (WNV, GSE77160-GSE77192). Experimental setup for evaluating a neural network trained to predict miRNA expression from mRNA data. The input data were trimmed to a range between 0 and 1. The panels show the training history (top-left), sample-level comparison (top-right), log fold change correlations (bottom-left), and p-value relationships (bottom-right) between predicted and true miRNA values.
8

## Slide 9
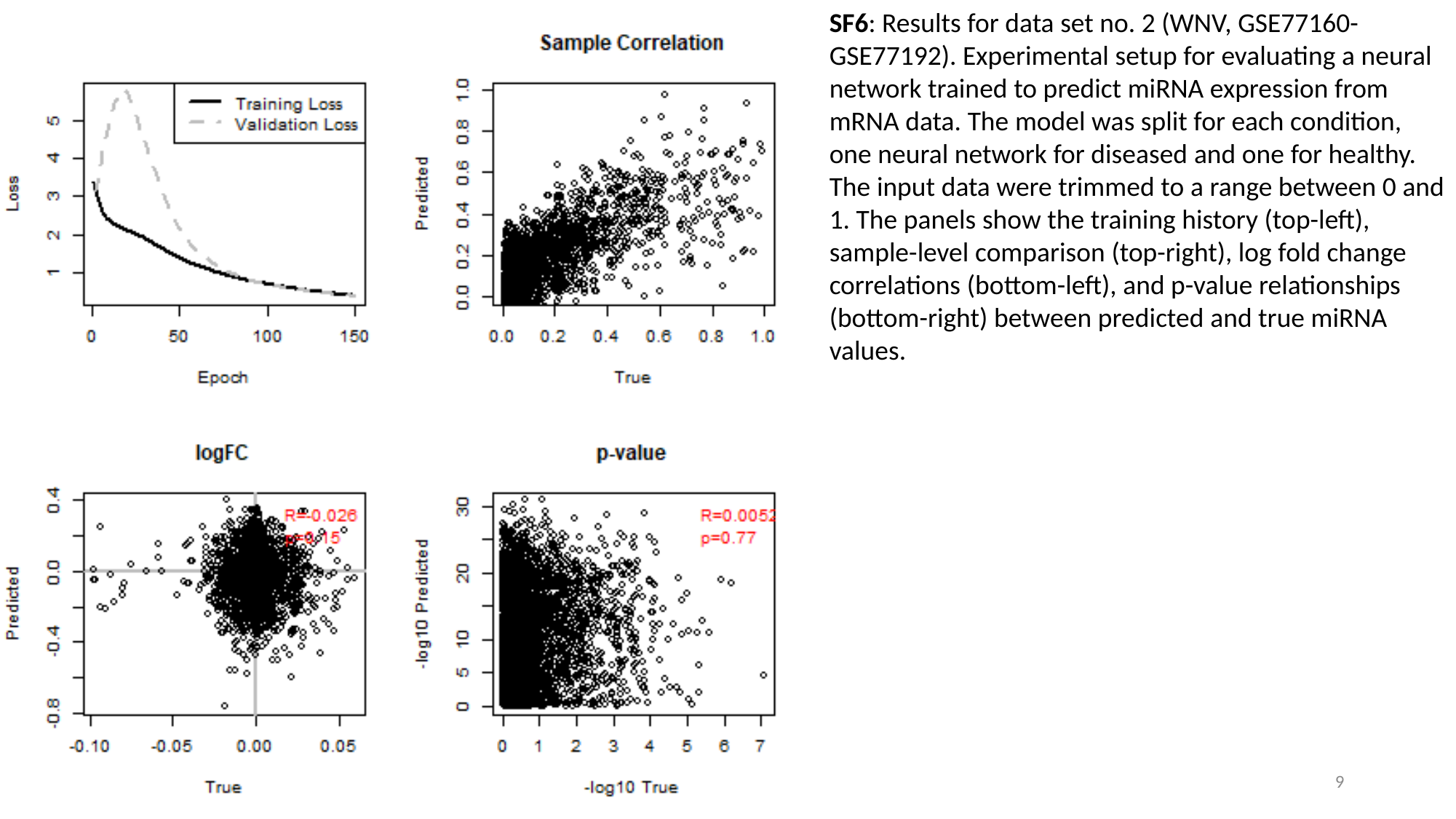

SF6: Results for data set no. 2 (WNV, GSE77160-GSE77192). Experimental setup for evaluating a neural network trained to predict miRNA expression from mRNA data. The model was split for each condition, one neural network for diseased and one for healthy. The input data were trimmed to a range between 0 and 1. The panels show the training history (top-left), sample-level comparison (top-right), log fold change correlations (bottom-left), and p-value relationships (bottom-right) between predicted and true miRNA values.
9

## Slide 10
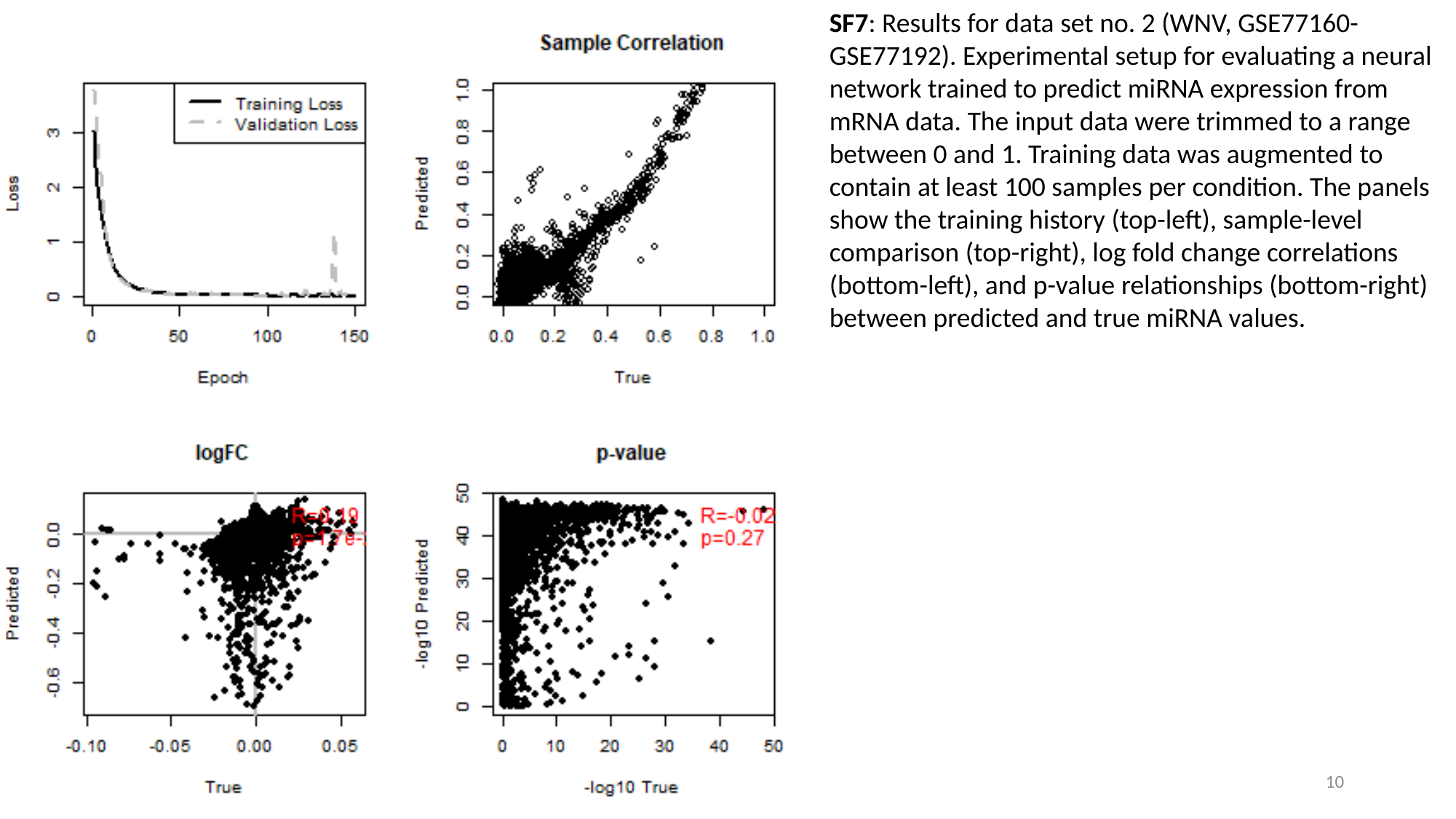

SF7: Results for data set no. 2 (WNV, GSE77160-GSE77192). Experimental setup for evaluating a neural network trained to predict miRNA expression from mRNA data. The input data were trimmed to a range between 0 and 1. Training data was augmented to contain at least 100 samples per condition. The panels show the training history (top-left), sample-level comparison (top-right), log fold change correlations (bottom-left), and p-value relationships (bottom-right) between predicted and true miRNA values.
10

## Slide 11
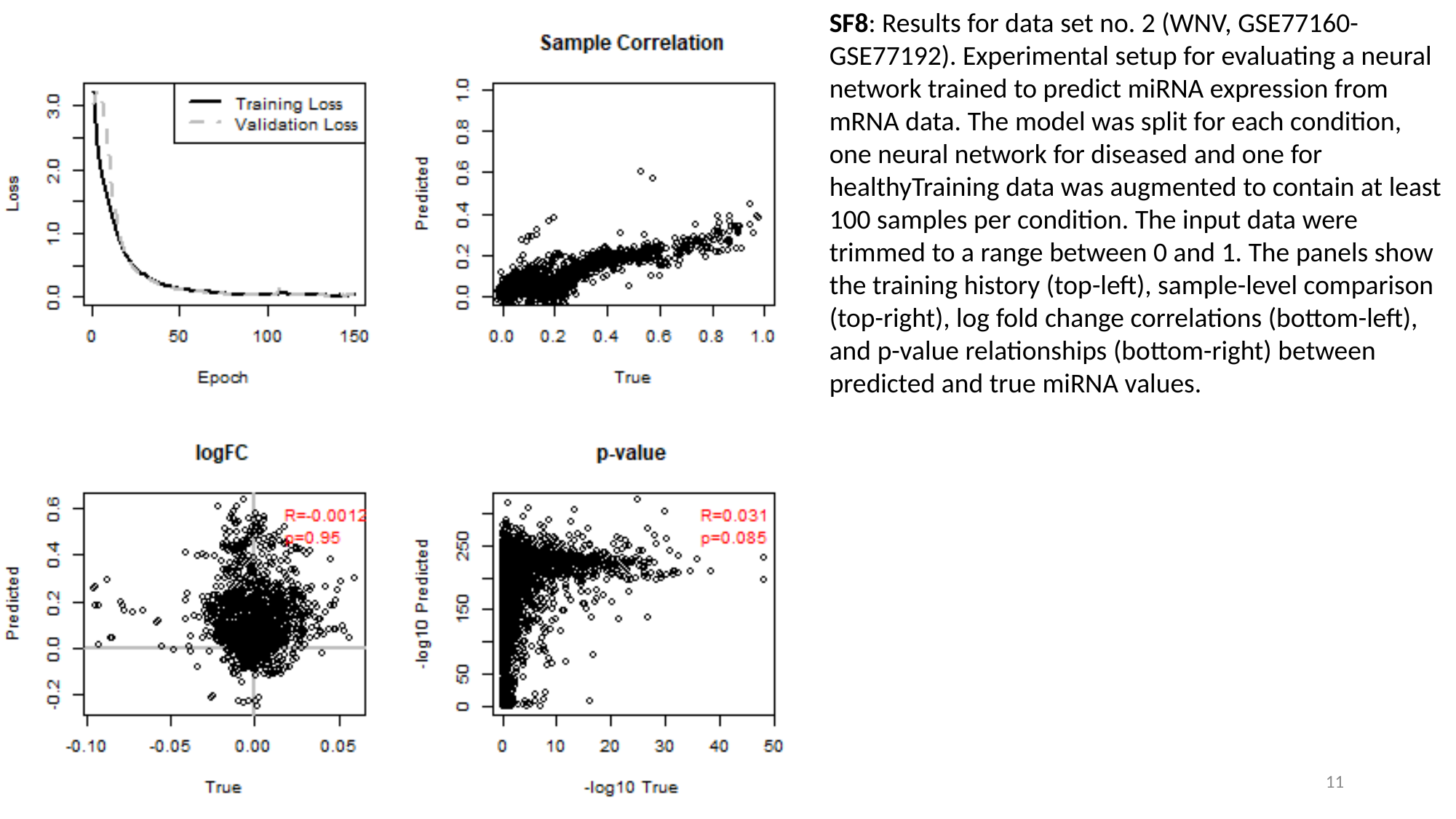

SF8: Results for data set no. 2 (WNV, GSE77160-GSE77192). Experimental setup for evaluating a neural network trained to predict miRNA expression from mRNA data. The model was split for each condition, one neural network for diseased and one for healthyTraining data was augmented to contain at least 100 samples per condition. The input data were trimmed to a range between 0 and 1. The panels show the training history (top-left), sample-level comparison (top-right), log fold change correlations (bottom-left), and p-value relationships (bottom-right) between predicted and true miRNA values.
11

## Slide 12
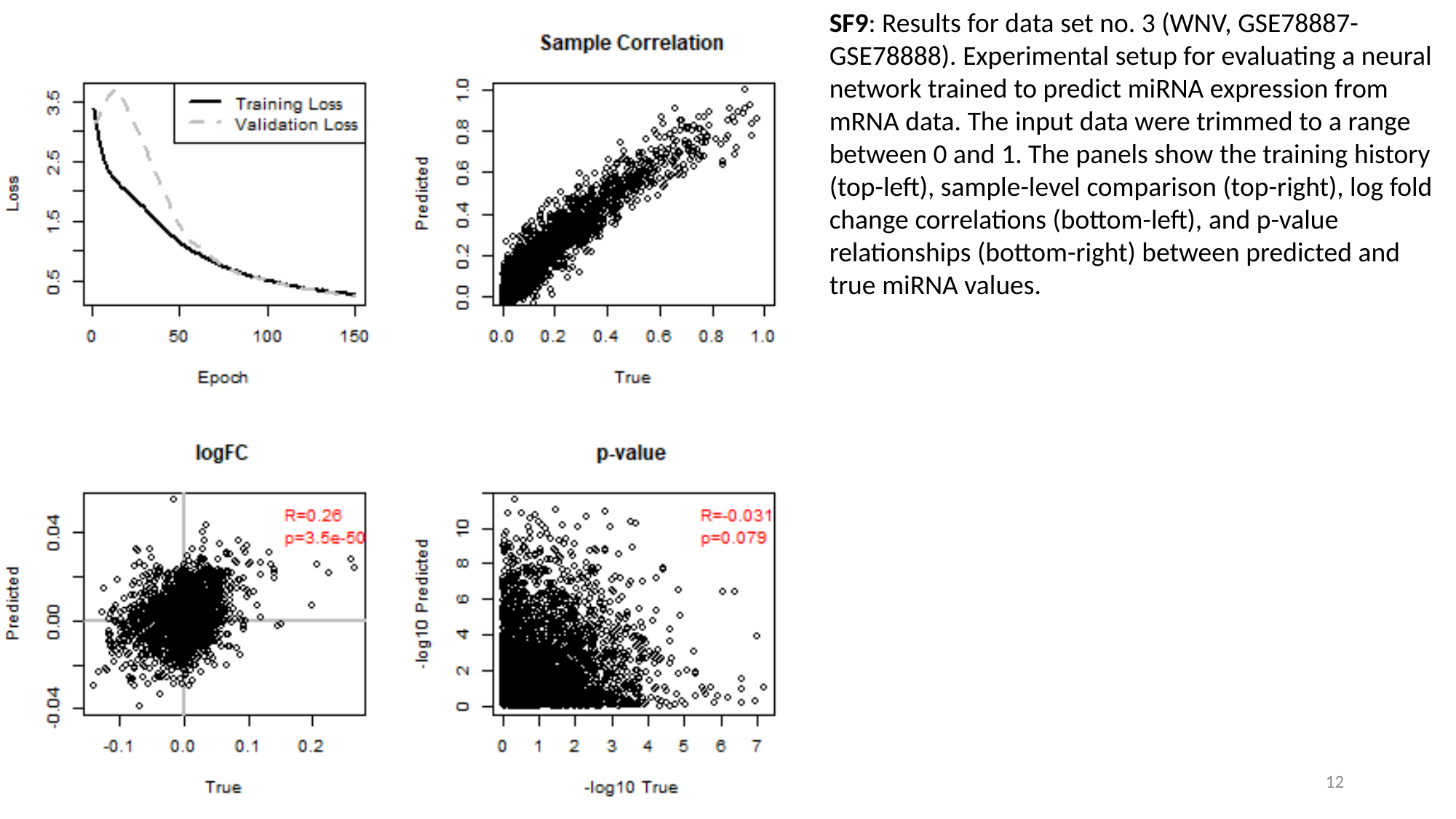

SF9: Results for data set no. 3 (WNV, GSE78887-GSE78888). Experimental setup for evaluating a neural network trained to predict miRNA expression from mRNA data. The input data were trimmed to a range between 0 and 1. The panels show the training history (top-left), sample-level comparison (top-right), log fold change correlations (bottom-left), and p-value relationships (bottom-right) between predicted and true miRNA values.
12

## Slide 13
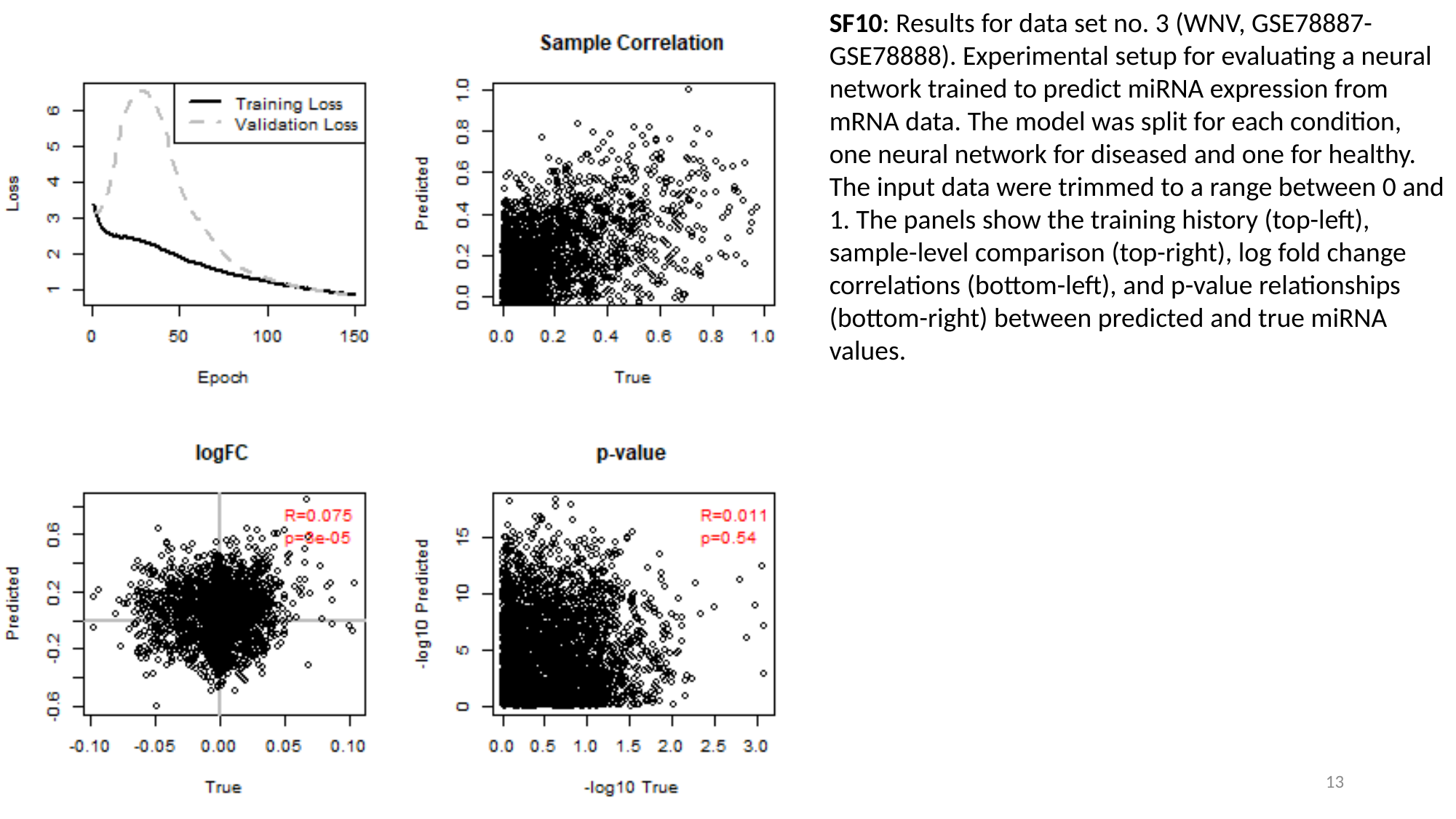

SF10: Results for data set no. 3 (WNV, GSE78887-GSE78888). Experimental setup for evaluating a neural network trained to predict miRNA expression from mRNA data. The model was split for each condition, one neural network for diseased and one for healthy. The input data were trimmed to a range between 0 and 1. The panels show the training history (top-left), sample-level comparison (top-right), log fold change correlations (bottom-left), and p-value relationships (bottom-right) between predicted and true miRNA values.
13

## Slide 14
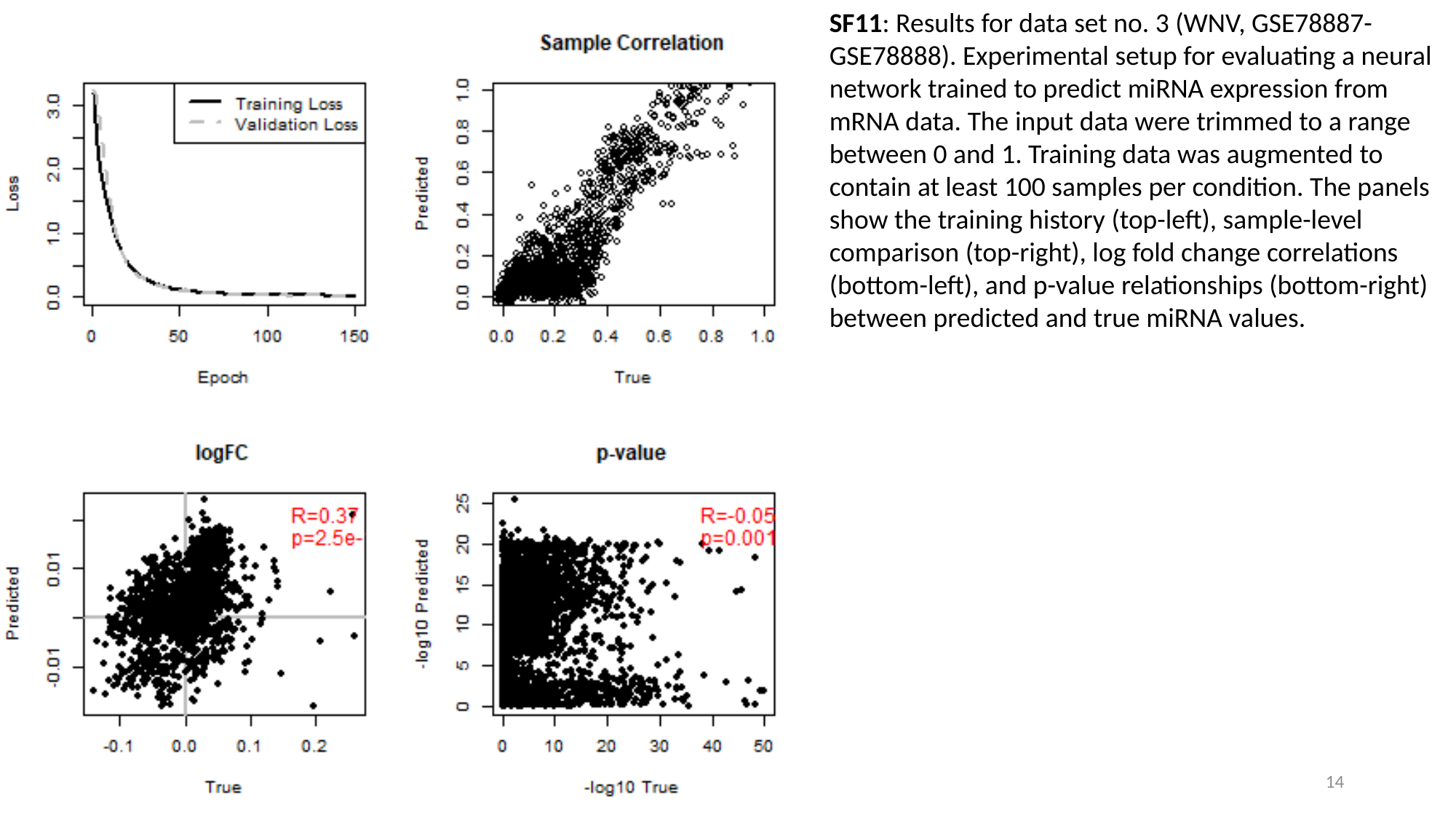

SF11: Results for data set no. 3 (WNV, GSE78887-GSE78888). Experimental setup for evaluating a neural network trained to predict miRNA expression from mRNA data. The input data were trimmed to a range between 0 and 1. Training data was augmented to contain at least 100 samples per condition. The panels show the training history (top-left), sample-level comparison (top-right), log fold change correlations (bottom-left), and p-value relationships (bottom-right) between predicted and true miRNA values.
14

## Slide 15
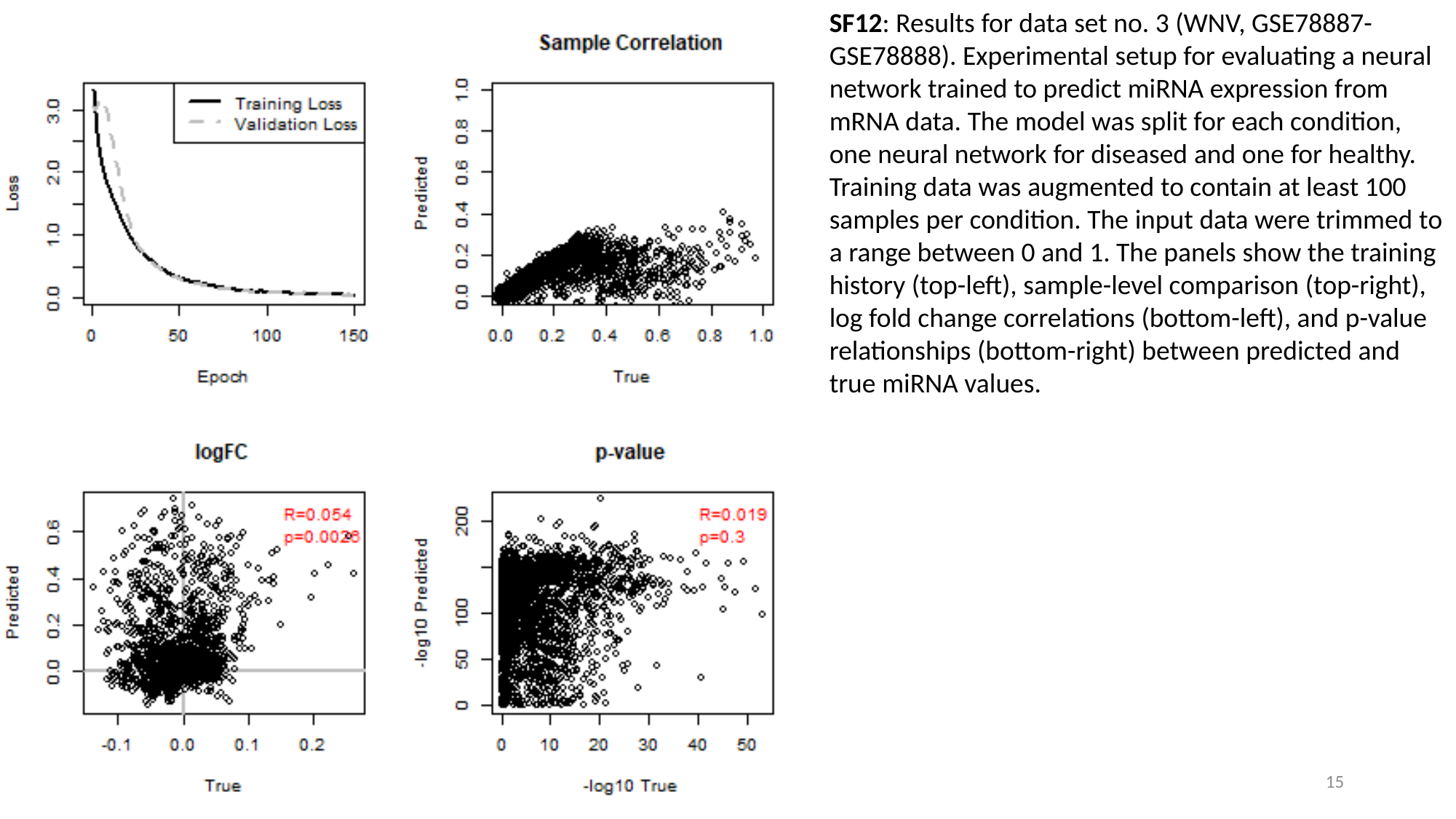

SF12: Results for data set no. 3 (WNV, GSE78887-GSE78888). Experimental setup for evaluating a neural network trained to predict miRNA expression from mRNA data. The model was split for each condition, one neural network for diseased and one for healthy. Training data was augmented to contain at least 100 samples per condition. The input data were trimmed to a range between 0 and 1. The panels show the training history (top-left), sample-level comparison (top-right), log fold change correlations (bottom-left), and p-value relationships (bottom-right) between predicted and true miRNA values.
15

## Slide 16
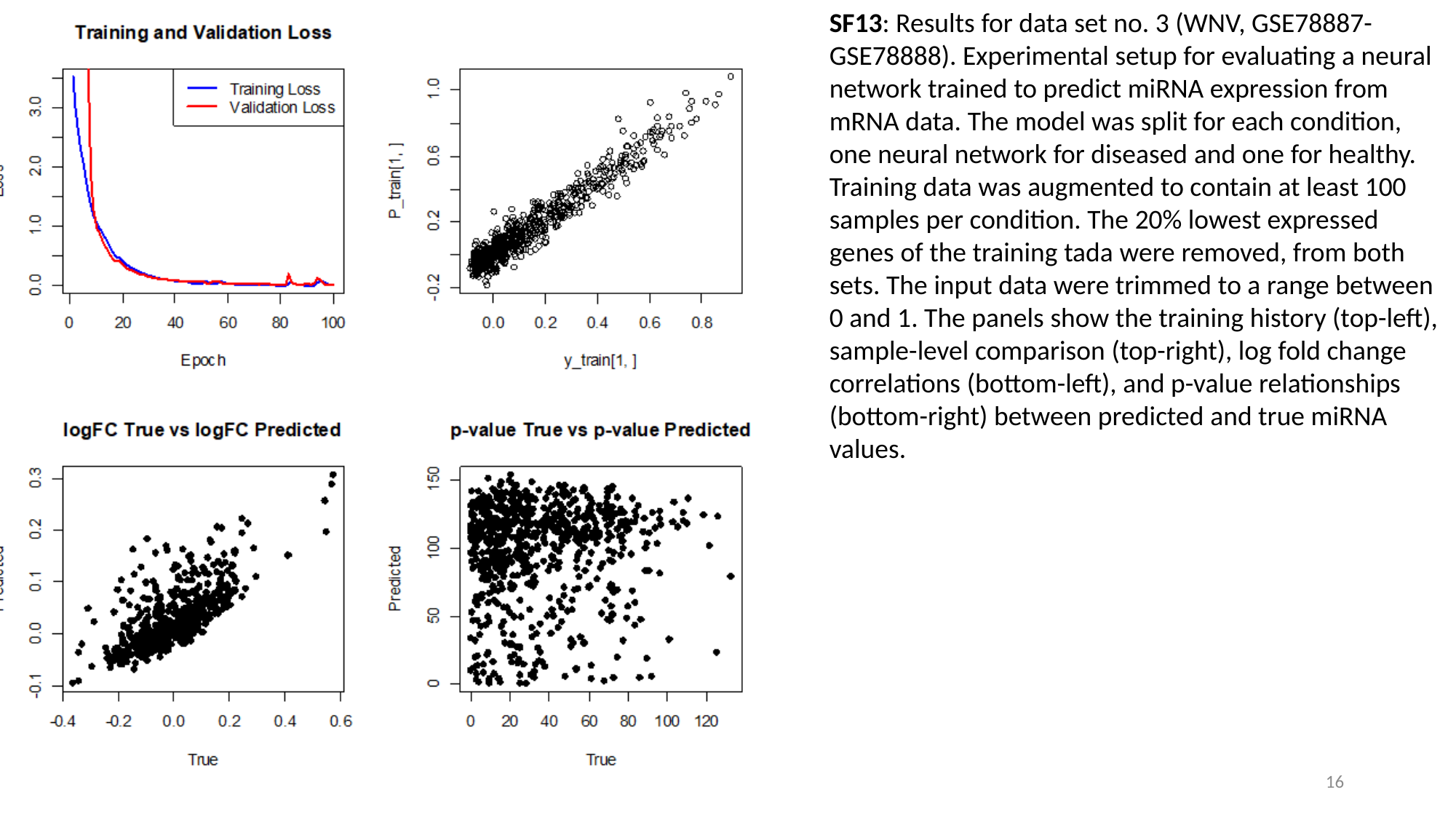

SF13: Results for data set no. 3 (WNV, GSE78887-GSE78888). Experimental setup for evaluating a neural network trained to predict miRNA expression from mRNA data. The model was split for each condition, one neural network for diseased and one for healthy. Training data was augmented to contain at least 100 samples per condition. The 20% lowest expressed genes of the training tada were removed, from both sets. The input data were trimmed to a range between 0 and 1. The panels show the training history (top-left), sample-level comparison (top-right), log fold change correlations (bottom-left), and p-value relationships (bottom-right) between predicted and true miRNA values.
16

## Slide 17
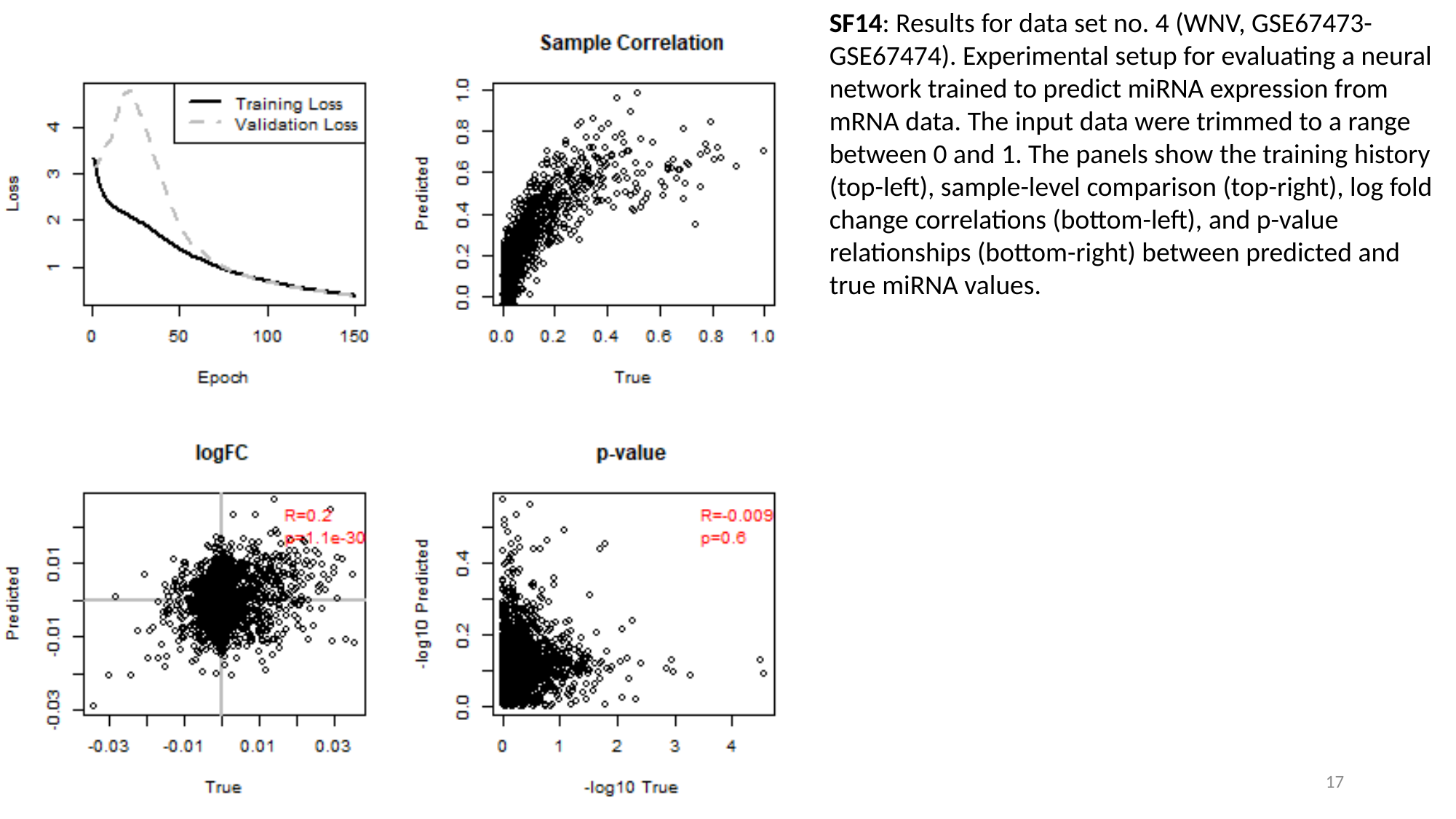

SF14: Results for data set no. 4 (WNV, GSE67473-GSE67474). Experimental setup for evaluating a neural network trained to predict miRNA expression from mRNA data. The input data were trimmed to a range between 0 and 1. The panels show the training history (top-left), sample-level comparison (top-right), log fold change correlations (bottom-left), and p-value relationships (bottom-right) between predicted and true miRNA values.
17

## Slide 18
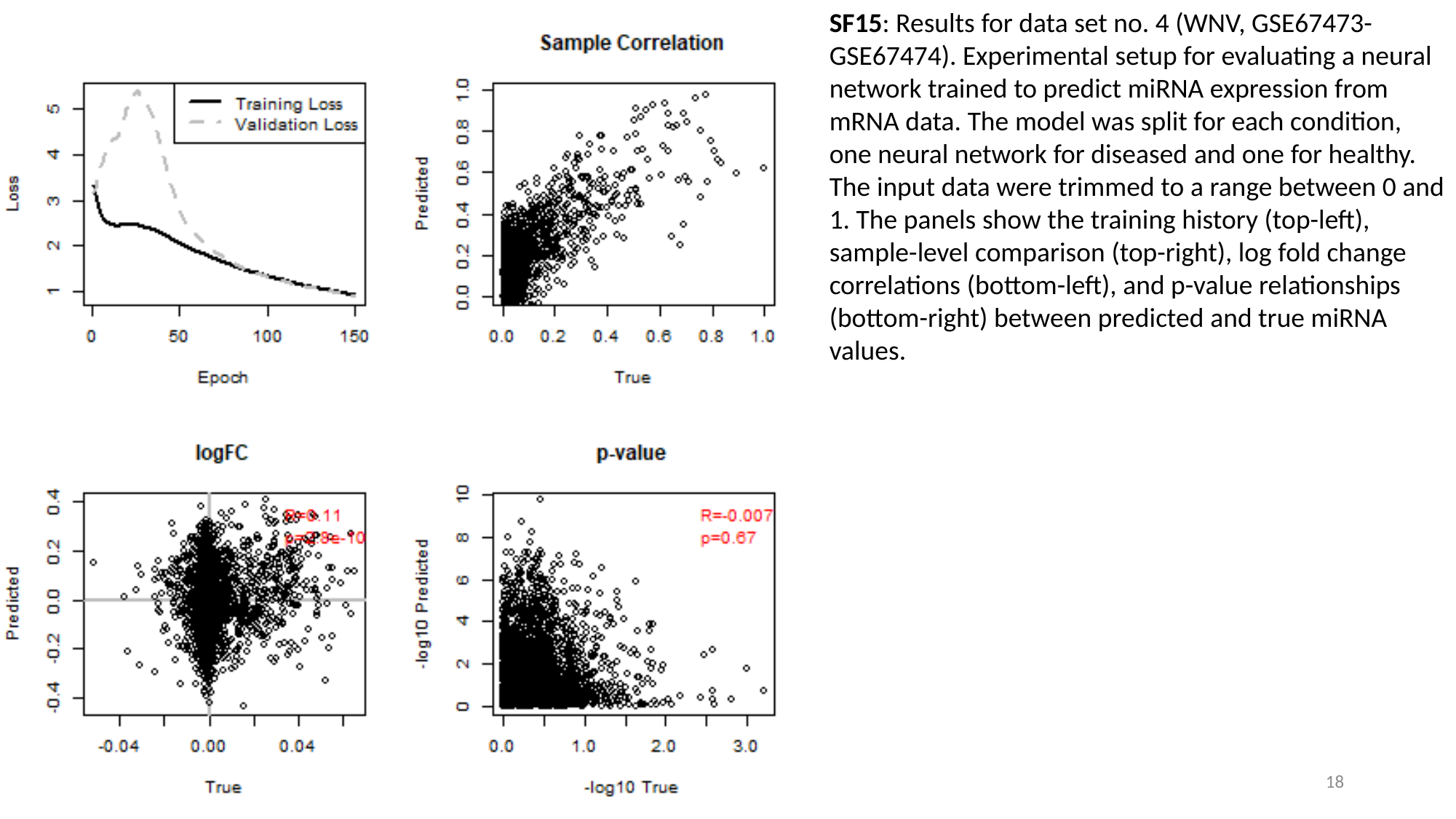

SF15: Results for data set no. 4 (WNV, GSE67473-GSE67474). Experimental setup for evaluating a neural network trained to predict miRNA expression from mRNA data. The model was split for each condition, one neural network for diseased and one for healthy. The input data were trimmed to a range between 0 and 1. The panels show the training history (top-left), sample-level comparison (top-right), log fold change correlations (bottom-left), and p-value relationships (bottom-right) between predicted and true miRNA values.
18

## Slide 19
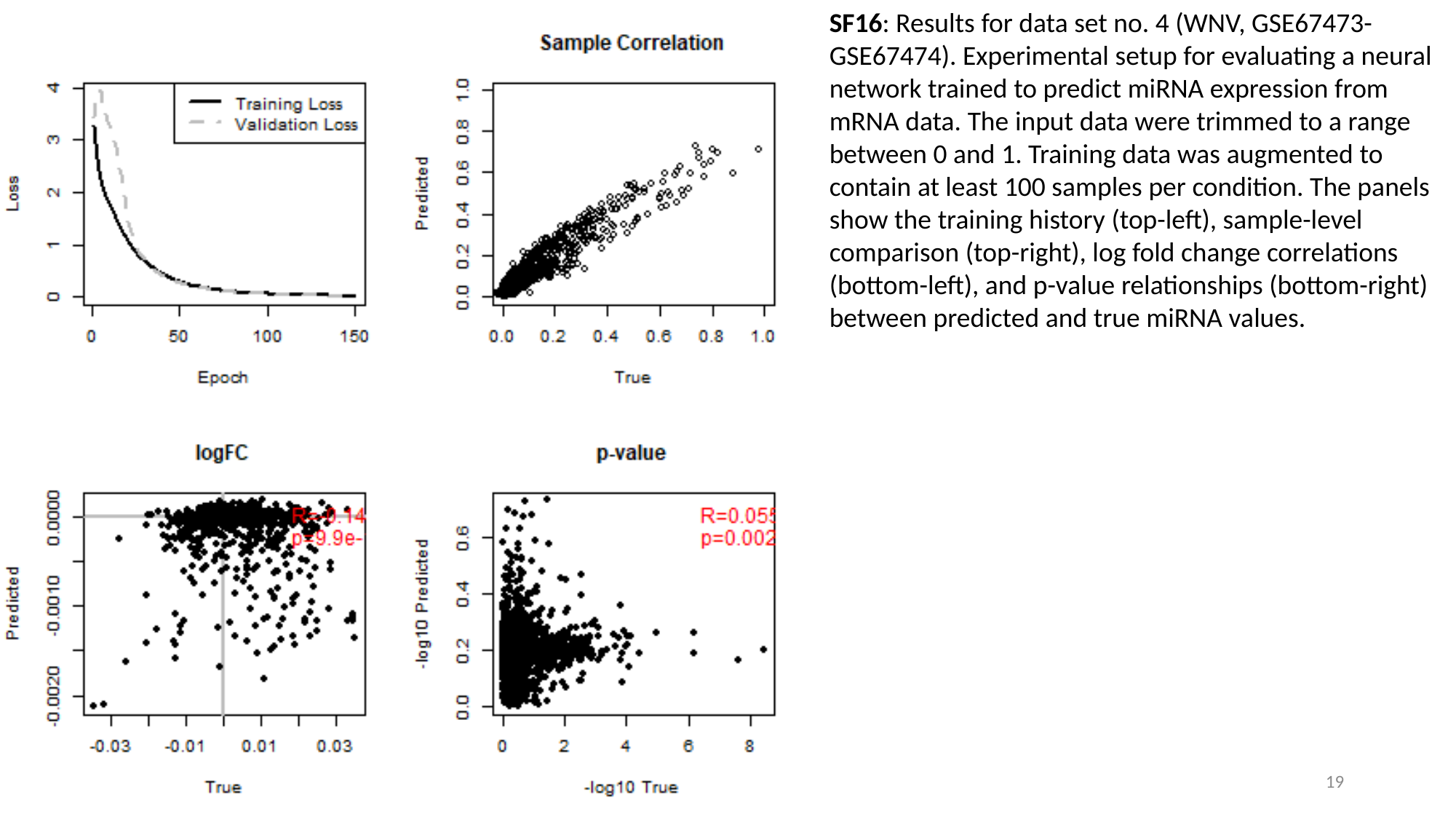

SF16: Results for data set no. 4 (WNV, GSE67473-GSE67474). Experimental setup for evaluating a neural network trained to predict miRNA expression from mRNA data. The input data were trimmed to a range between 0 and 1. Training data was augmented to contain at least 100 samples per condition. The panels show the training history (top-left), sample-level comparison (top-right), log fold change correlations (bottom-left), and p-value relationships (bottom-right) between predicted and true miRNA values.
19

## Slide 20
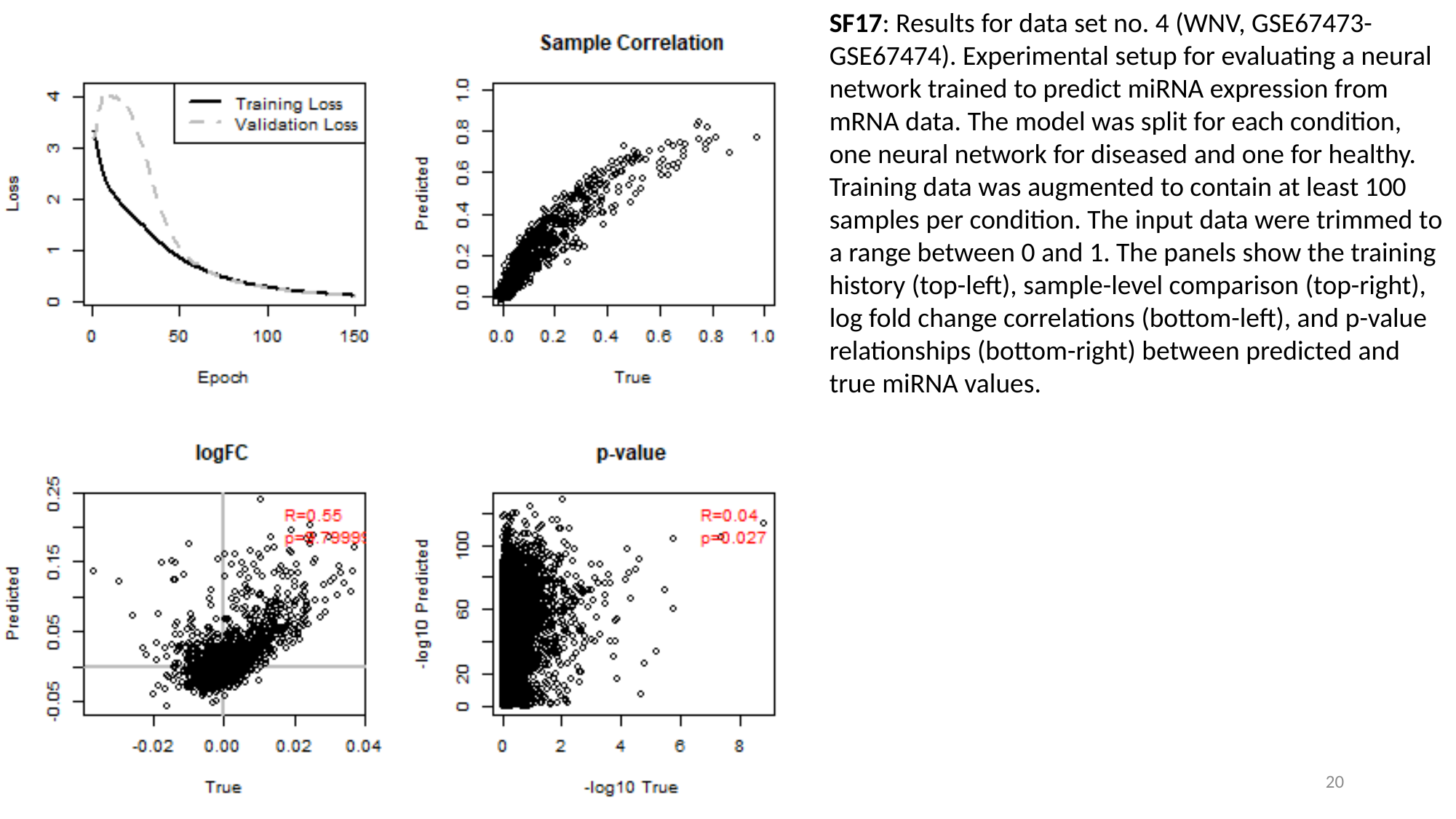

SF17: Results for data set no. 4 (WNV, GSE67473-GSE67474). Experimental setup for evaluating a neural network trained to predict miRNA expression from mRNA data. The model was split for each condition, one neural network for diseased and one for healthy. Training data was augmented to contain at least 100 samples per condition. The input data were trimmed to a range between 0 and 1. The panels show the training history (top-left), sample-level comparison (top-right), log fold change correlations (bottom-left), and p-value relationships (bottom-right) between predicted and true miRNA values.
20

## Slide 21
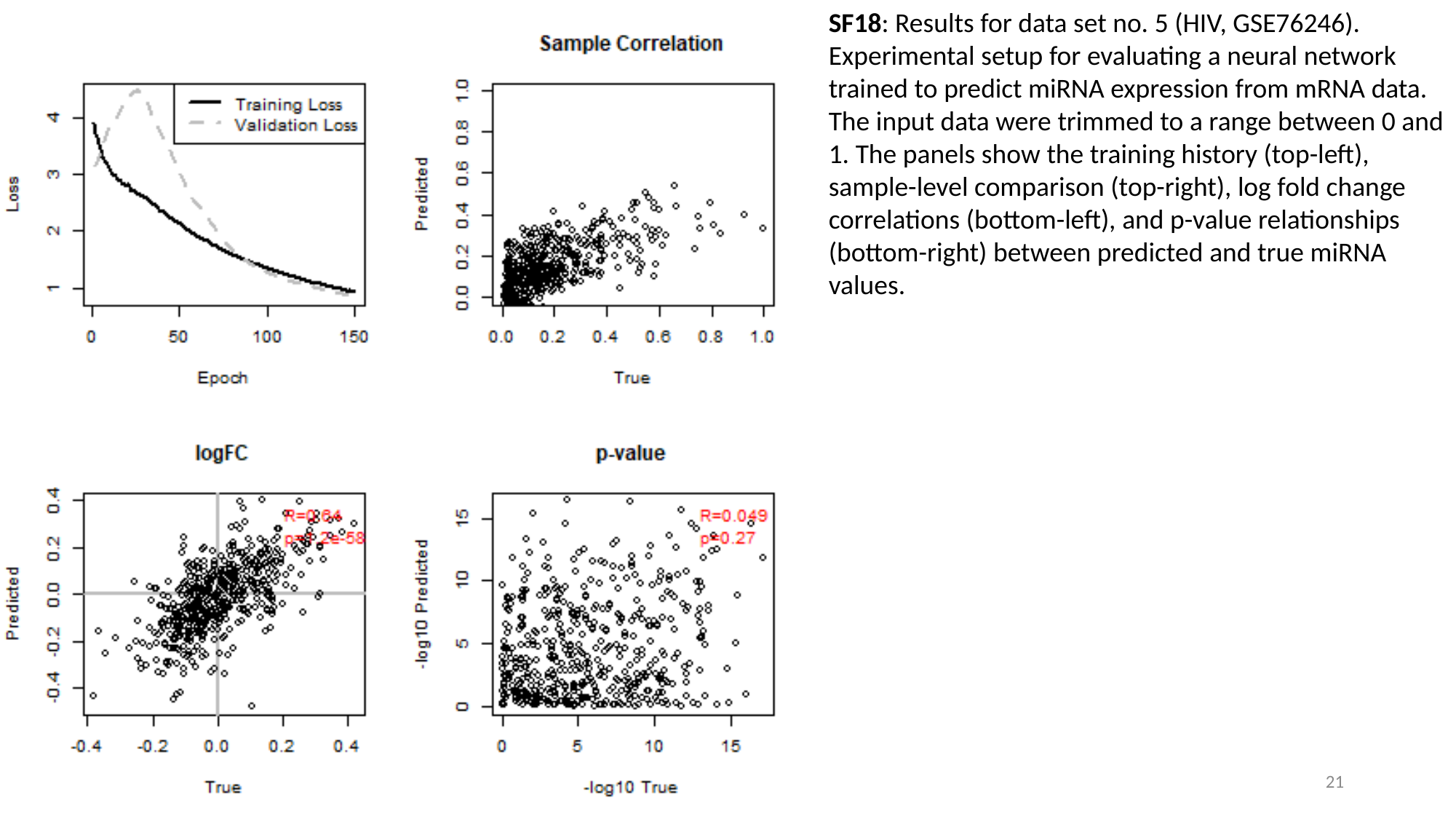

SF18: Results for data set no. 5 (HIV, GSE76246). Experimental setup for evaluating a neural network trained to predict miRNA expression from mRNA data. The input data were trimmed to a range between 0 and 1. The panels show the training history (top-left), sample-level comparison (top-right), log fold change correlations (bottom-left), and p-value relationships (bottom-right) between predicted and true miRNA values.
21

## Slide 22
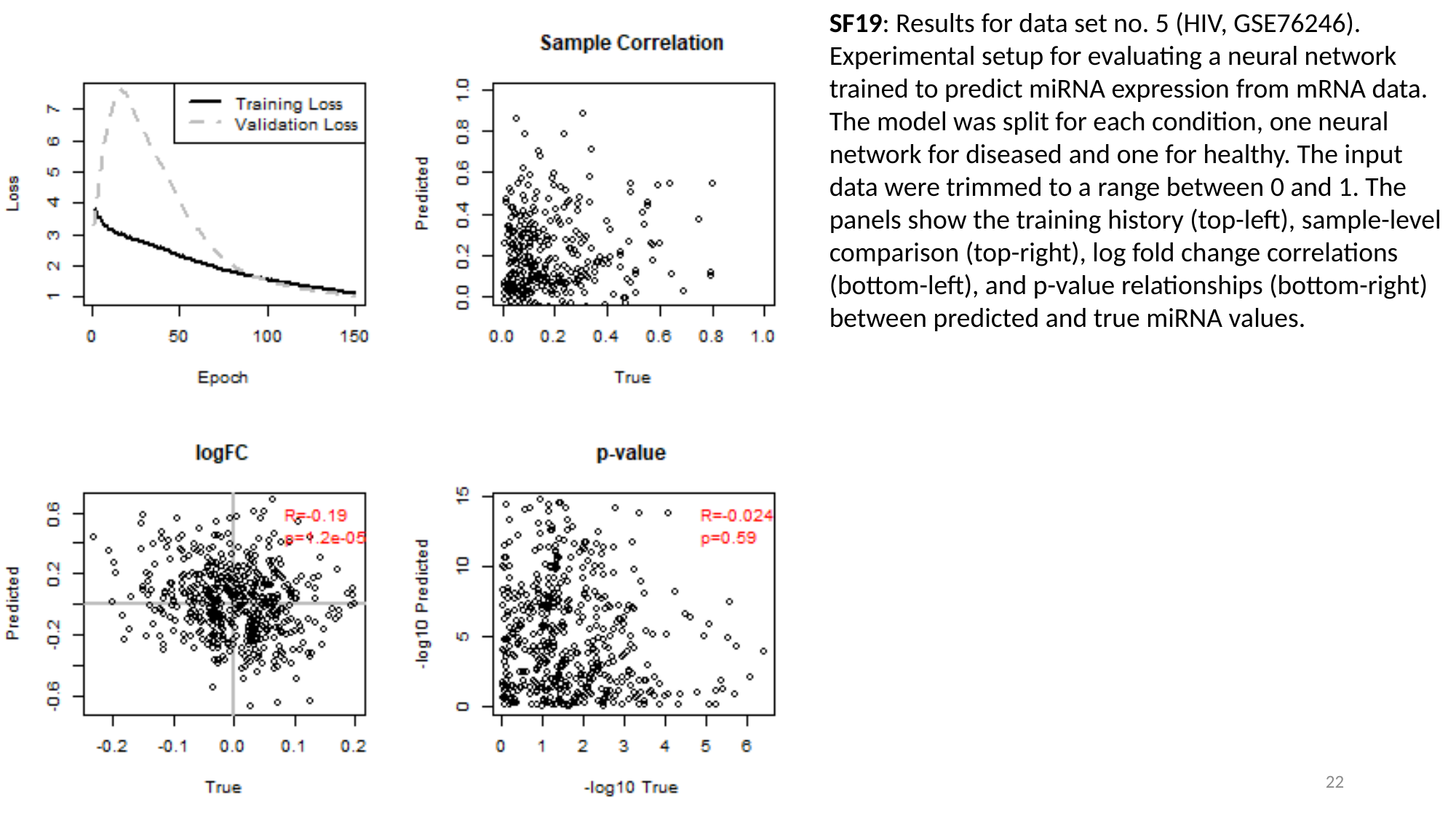

SF19: Results for data set no. 5 (HIV, GSE76246). Experimental setup for evaluating a neural network trained to predict miRNA expression from mRNA data. The model was split for each condition, one neural network for diseased and one for healthy. The input data were trimmed to a range between 0 and 1. The panels show the training history (top-left), sample-level comparison (top-right), log fold change correlations (bottom-left), and p-value relationships (bottom-right) between predicted and true miRNA values.
22

## Slide 23
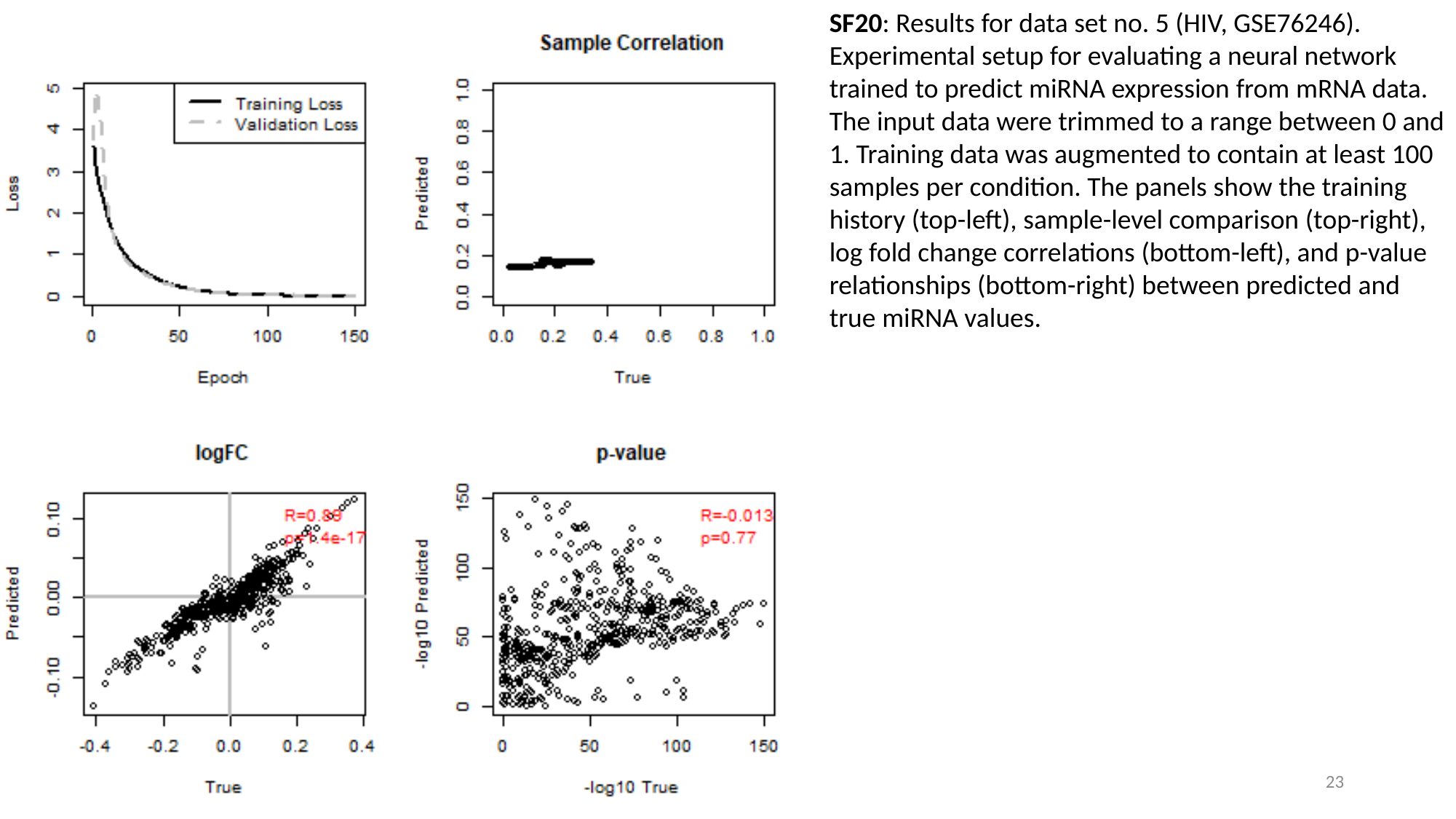

SF20: Results for data set no. 5 (HIV, GSE76246). Experimental setup for evaluating a neural network trained to predict miRNA expression from mRNA data. The input data were trimmed to a range between 0 and 1. Training data was augmented to contain at least 100 samples per condition. The panels show the training history (top-left), sample-level comparison (top-right), log fold change correlations (bottom-left), and p-value relationships (bottom-right) between predicted and true miRNA values.
23

## Slide 24
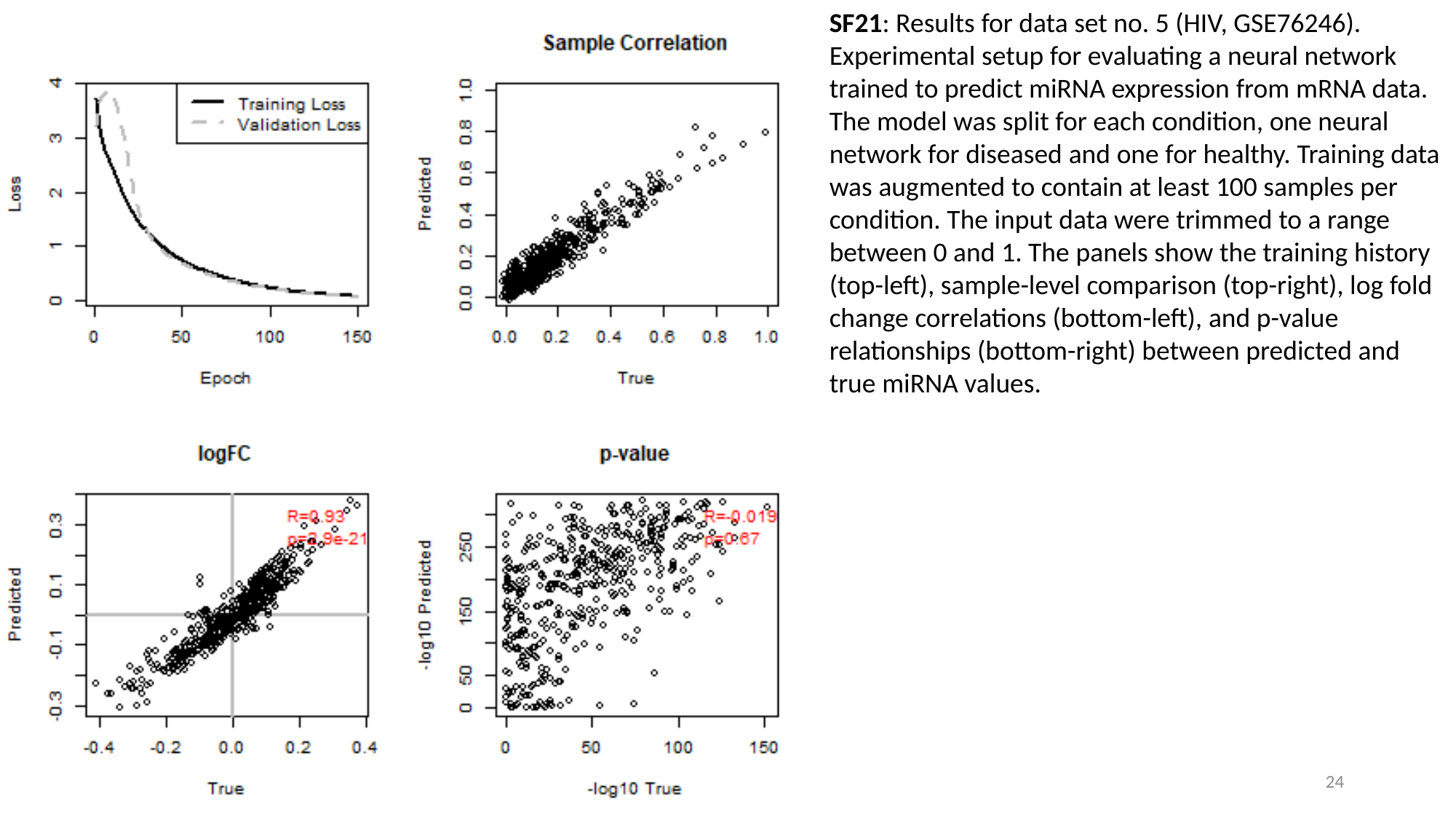

SF21: Results for data set no. 5 (HIV, GSE76246). Experimental setup for evaluating a neural network trained to predict miRNA expression from mRNA data. The model was split for each condition, one neural network for diseased and one for healthy. Training data was augmented to contain at least 100 samples per condition. The input data were trimmed to a range between 0 and 1. The panels show the training history (top-left), sample-level comparison (top-right), log fold change correlations (bottom-left), and p-value relationships (bottom-right) between predicted and true miRNA values.
24

## Slide 25
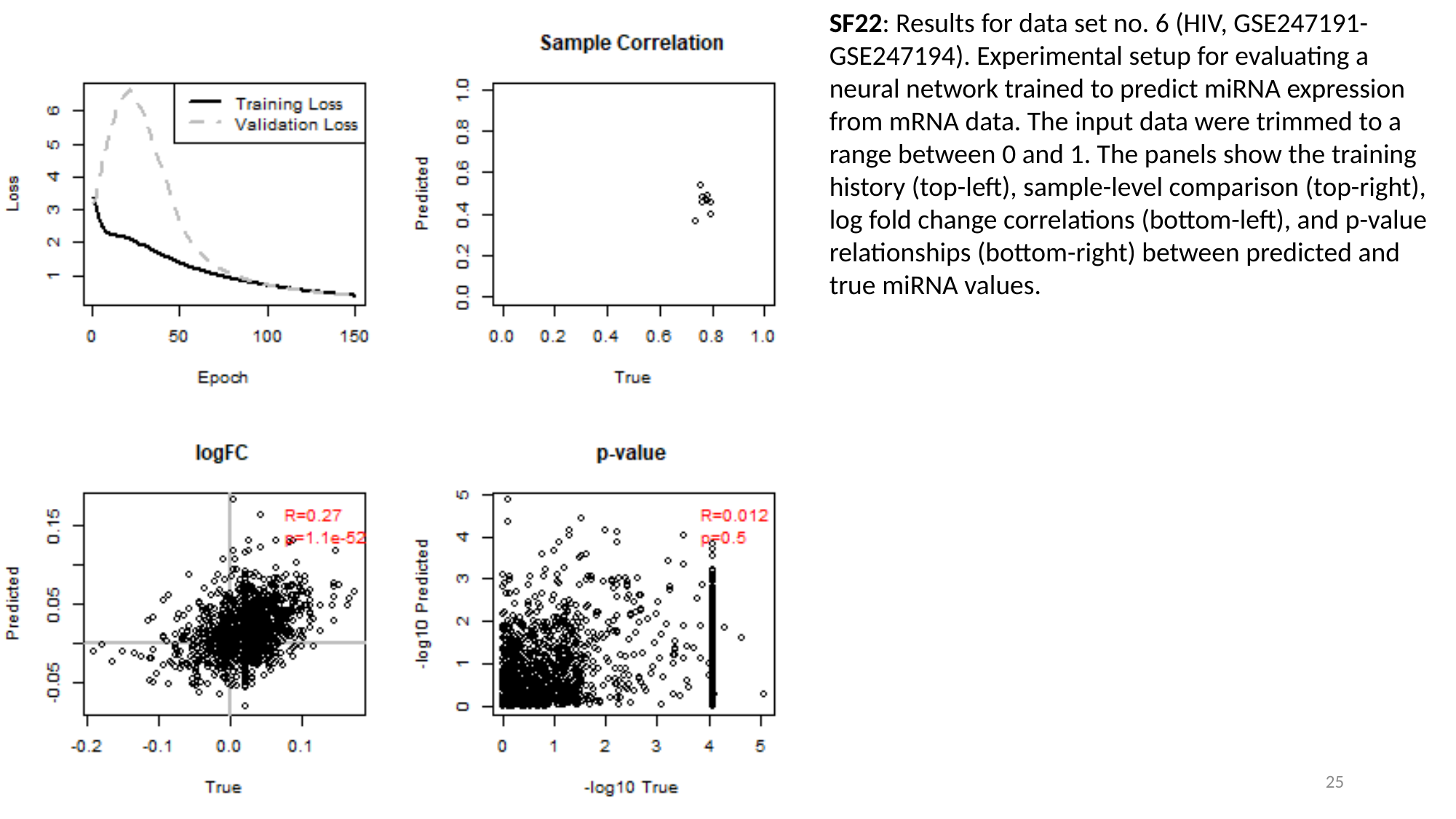

SF22: Results for data set no. 6 (HIV, GSE247191-GSE247194). Experimental setup for evaluating a neural network trained to predict miRNA expression from mRNA data. The input data were trimmed to a range between 0 and 1. The panels show the training history (top-left), sample-level comparison (top-right), log fold change correlations (bottom-left), and p-value relationships (bottom-right) between predicted and true miRNA values.
25

## Slide 26
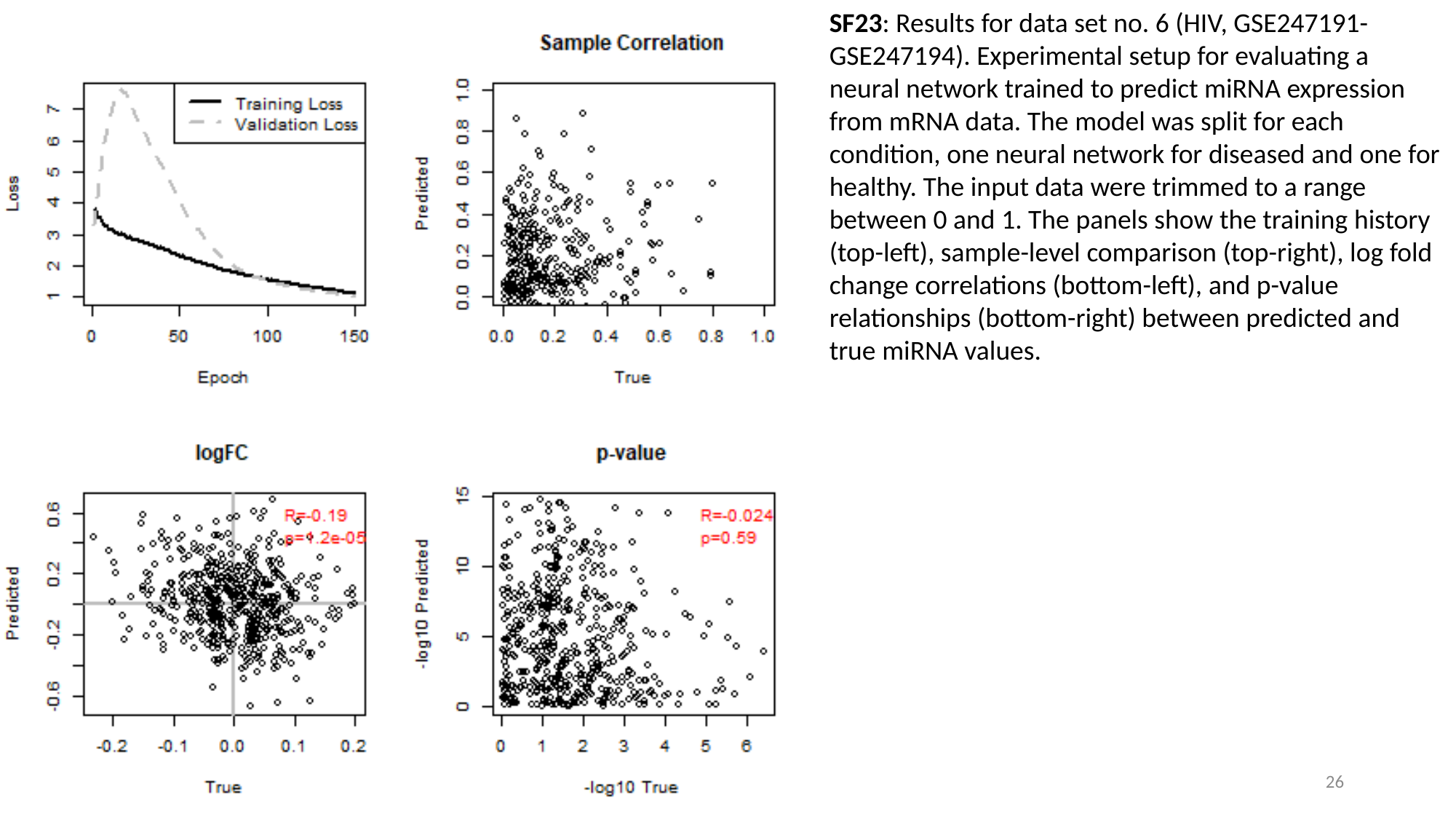

SF23: Results for data set no. 6 (HIV, GSE247191-GSE247194). Experimental setup for evaluating a neural network trained to predict miRNA expression from mRNA data. The model was split for each condition, one neural network for diseased and one for healthy. The input data were trimmed to a range between 0 and 1. The panels show the training history (top-left), sample-level comparison (top-right), log fold change correlations (bottom-left), and p-value relationships (bottom-right) between predicted and true miRNA values.
26

## Slide 27
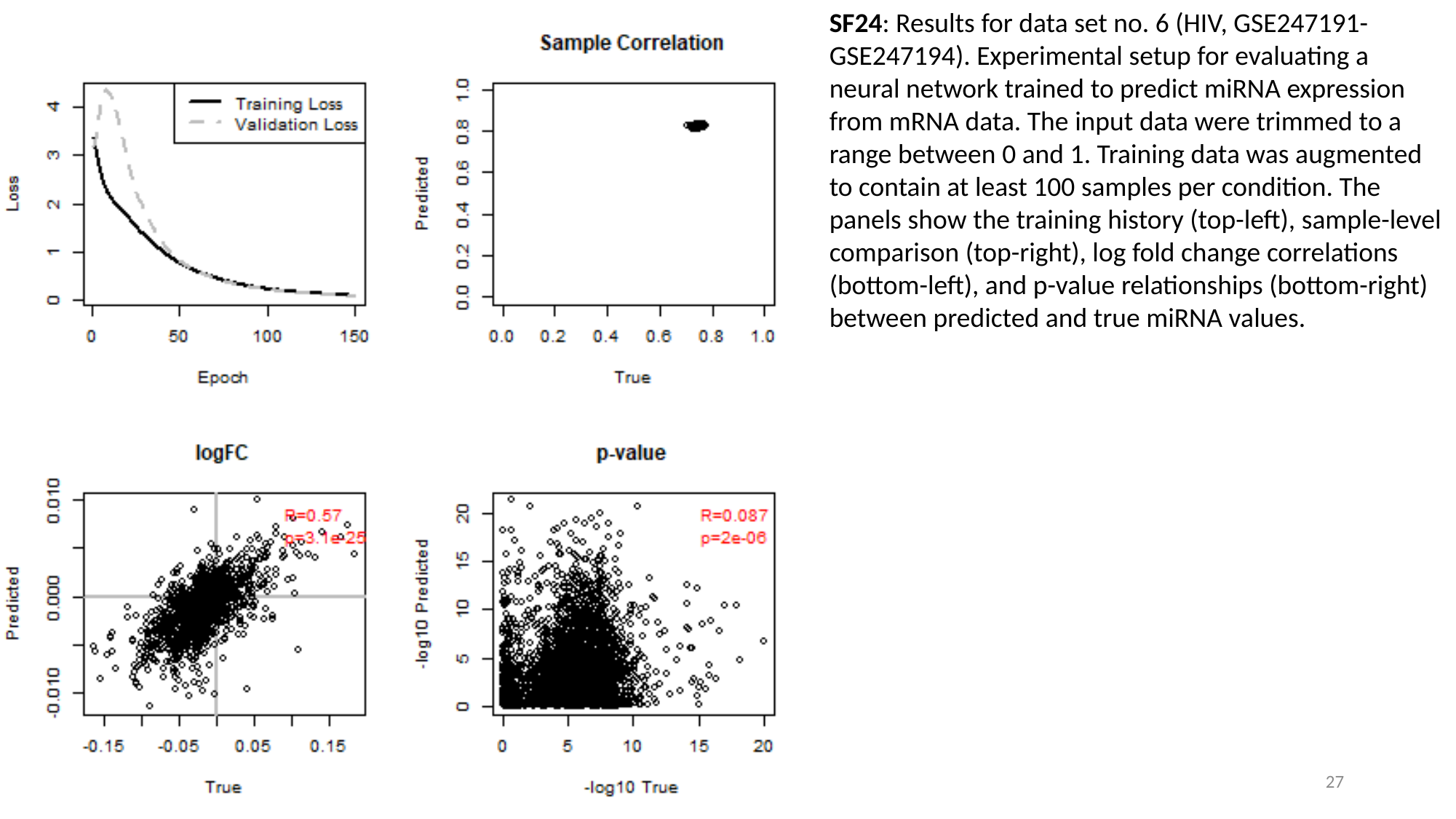

SF24: Results for data set no. 6 (HIV, GSE247191-GSE247194). Experimental setup for evaluating a neural network trained to predict miRNA expression from mRNA data. The input data were trimmed to a range between 0 and 1. Training data was augmented to contain at least 100 samples per condition. The panels show the training history (top-left), sample-level comparison (top-right), log fold change correlations (bottom-left), and p-value relationships (bottom-right) between predicted and true miRNA values.
27

## Slide 28
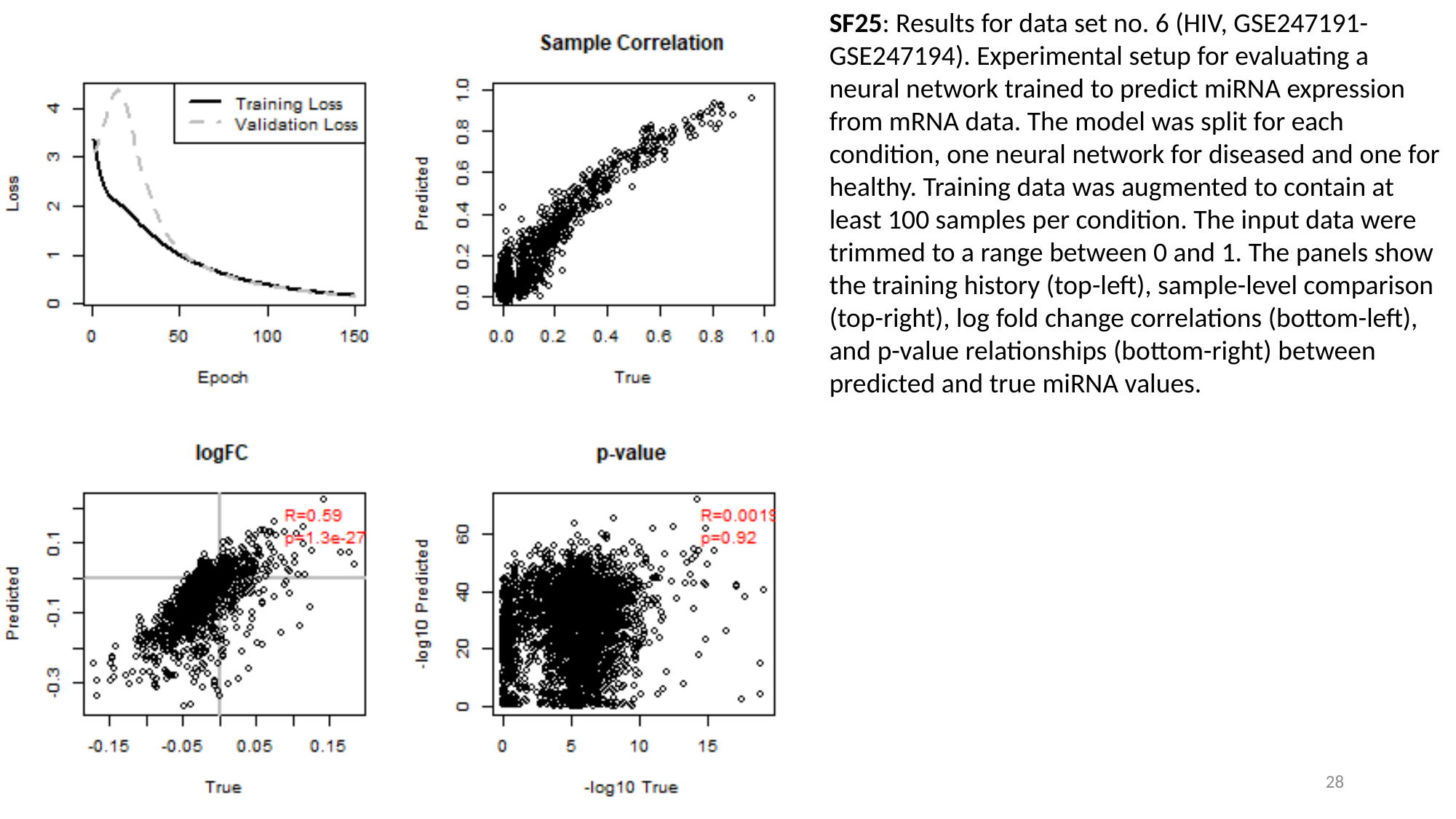

SF25: Results for data set no. 6 (HIV, GSE247191-GSE247194). Experimental setup for evaluating a neural network trained to predict miRNA expression from mRNA data. The model was split for each condition, one neural network for diseased and one for healthy. Training data was augmented to contain at least 100 samples per condition. The input data were trimmed to a range between 0 and 1. The panels show the training history (top-left), sample-level comparison (top-right), log fold change correlations (bottom-left), and p-value relationships (bottom-right) between predicted and true miRNA values.
28

## Slide 29
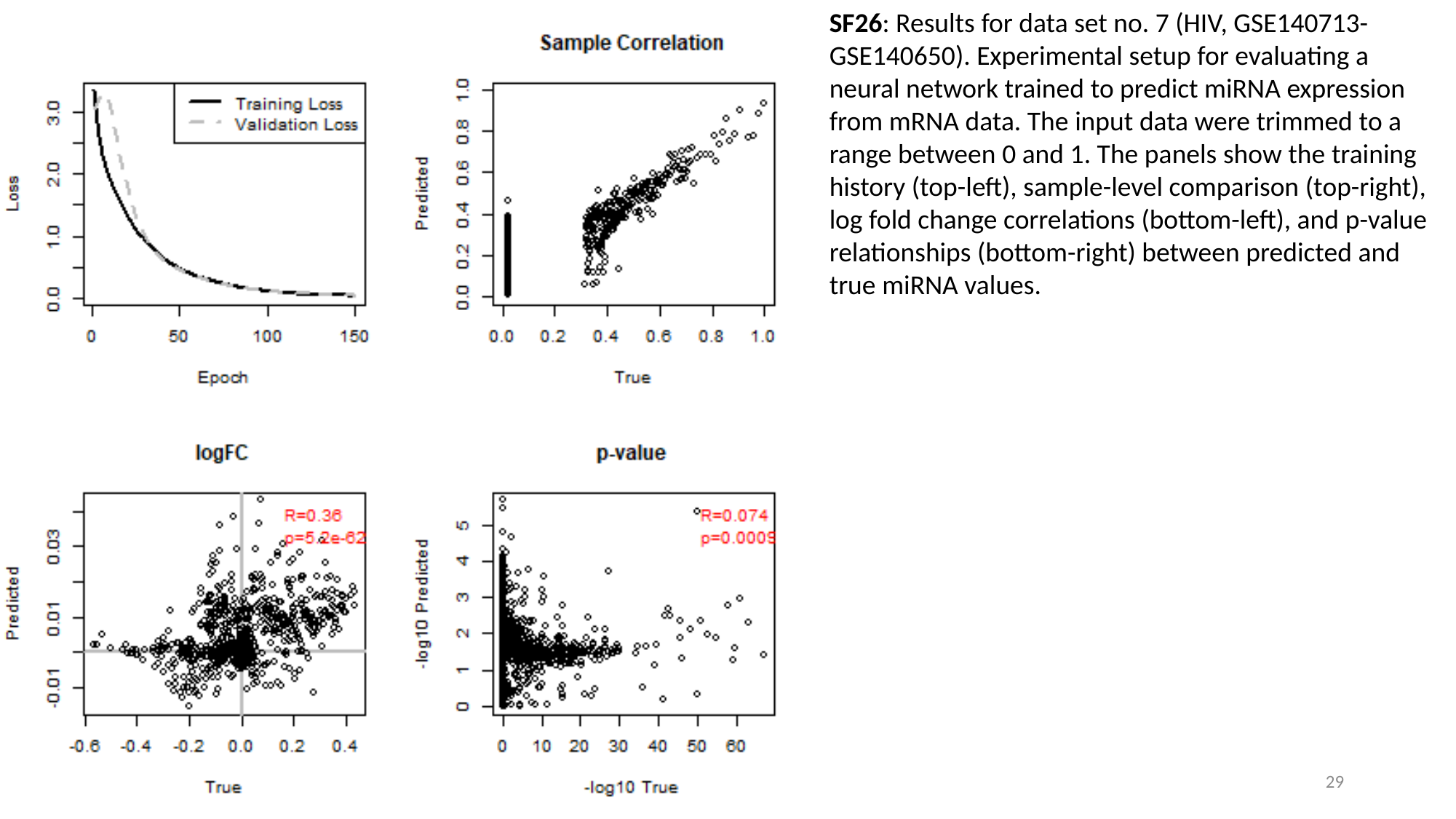

SF26: Results for data set no. 7 (HIV, GSE140713-GSE140650). Experimental setup for evaluating a neural network trained to predict miRNA expression from mRNA data. The input data were trimmed to a range between 0 and 1. The panels show the training history (top-left), sample-level comparison (top-right), log fold change correlations (bottom-left), and p-value relationships (bottom-right) between predicted and true miRNA values.
29

## Slide 30
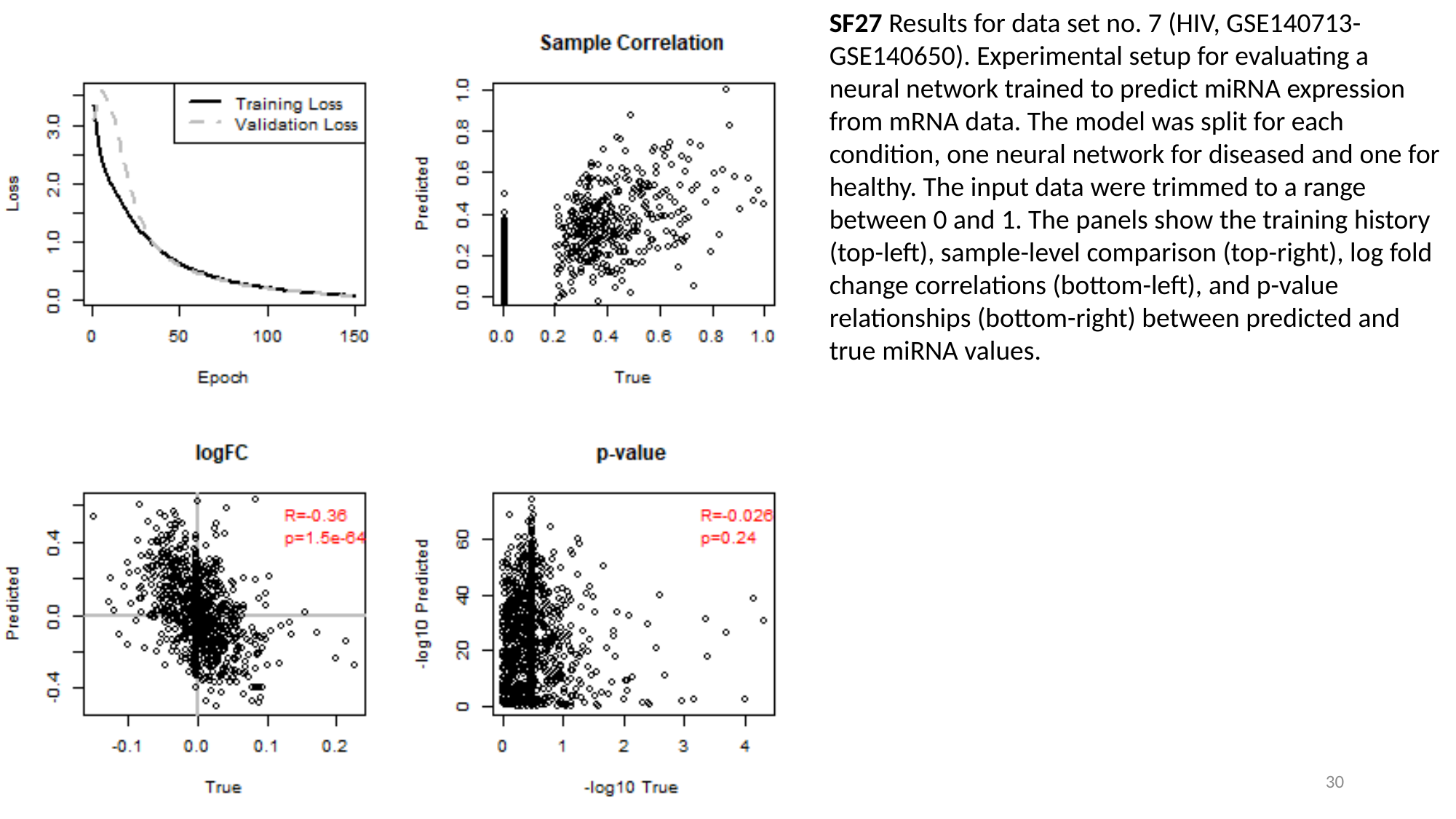

SF27 Results for data set no. 7 (HIV, GSE140713-GSE140650). Experimental setup for evaluating a neural network trained to predict miRNA expression from mRNA data. The model was split for each condition, one neural network for diseased and one for healthy. The input data were trimmed to a range between 0 and 1. The panels show the training history (top-left), sample-level comparison (top-right), log fold change correlations (bottom-left), and p-value relationships (bottom-right) between predicted and true miRNA values.
30

## Slide 31
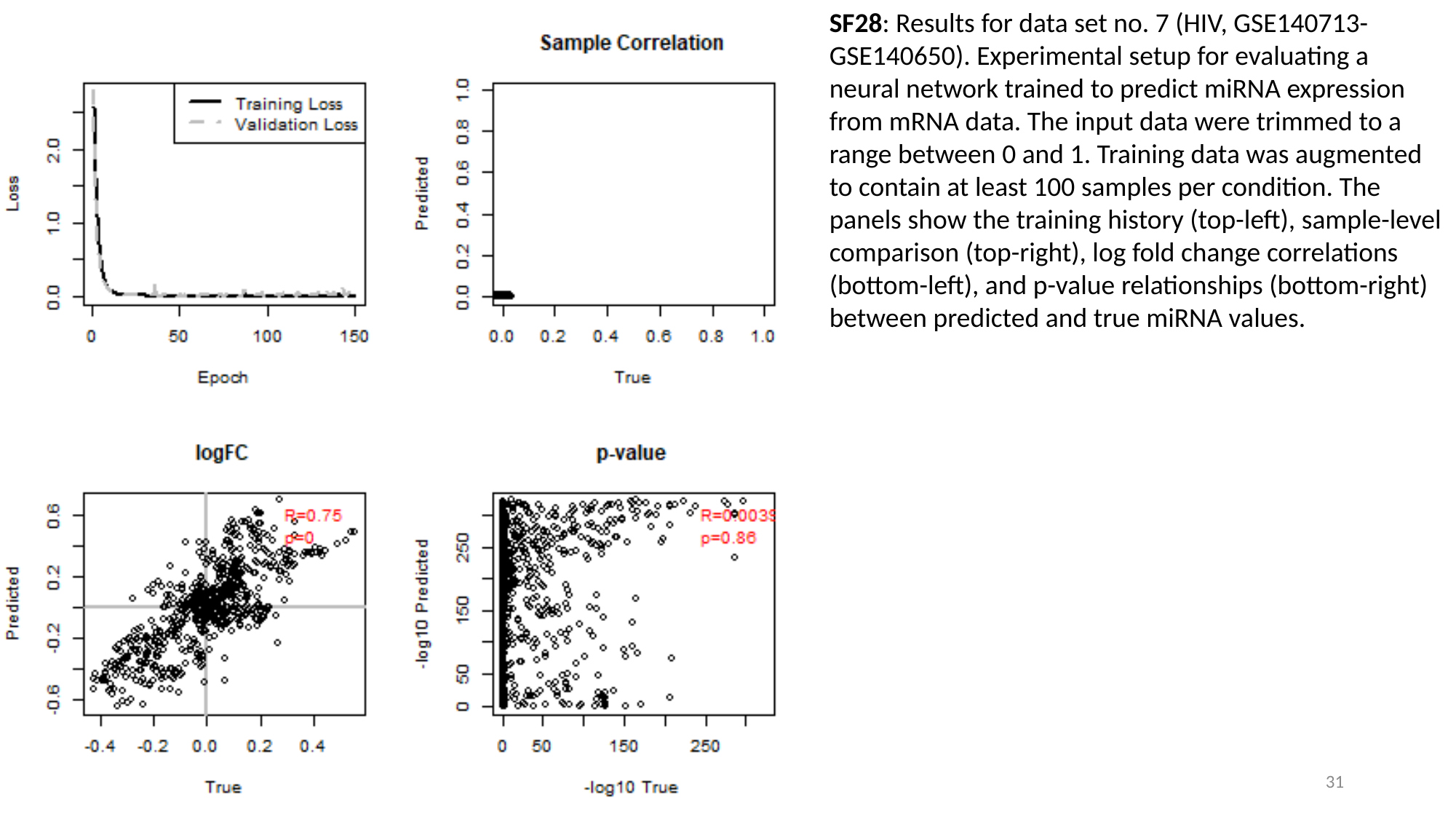

SF28: Results for data set no. 7 (HIV, GSE140713-GSE140650). Experimental setup for evaluating a neural network trained to predict miRNA expression from mRNA data. The input data were trimmed to a range between 0 and 1. Training data was augmented to contain at least 100 samples per condition. The panels show the training history (top-left), sample-level comparison (top-right), log fold change correlations (bottom-left), and p-value relationships (bottom-right) between predicted and true miRNA values.
31

## Slide 32
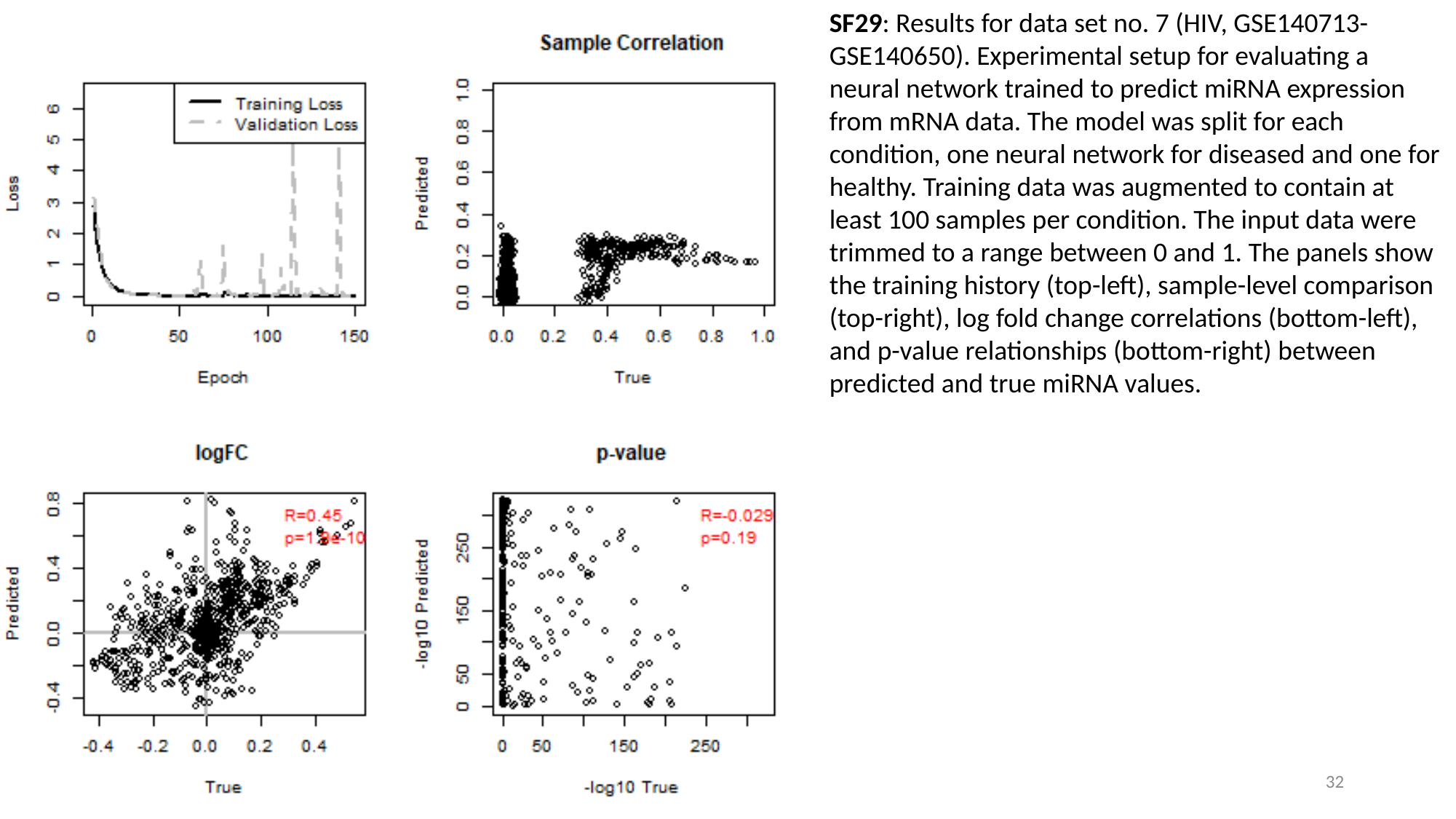

SF29: Results for data set no. 7 (HIV, GSE140713-GSE140650). Experimental setup for evaluating a neural network trained to predict miRNA expression from mRNA data. The model was split for each condition, one neural network for diseased and one for healthy. Training data was augmented to contain at least 100 samples per condition. The input data were trimmed to a range between 0 and 1. The panels show the training history (top-left), sample-level comparison (top-right), log fold change correlations (bottom-left), and p-value relationships (bottom-right) between predicted and true miRNA values.
32

## Slide 33
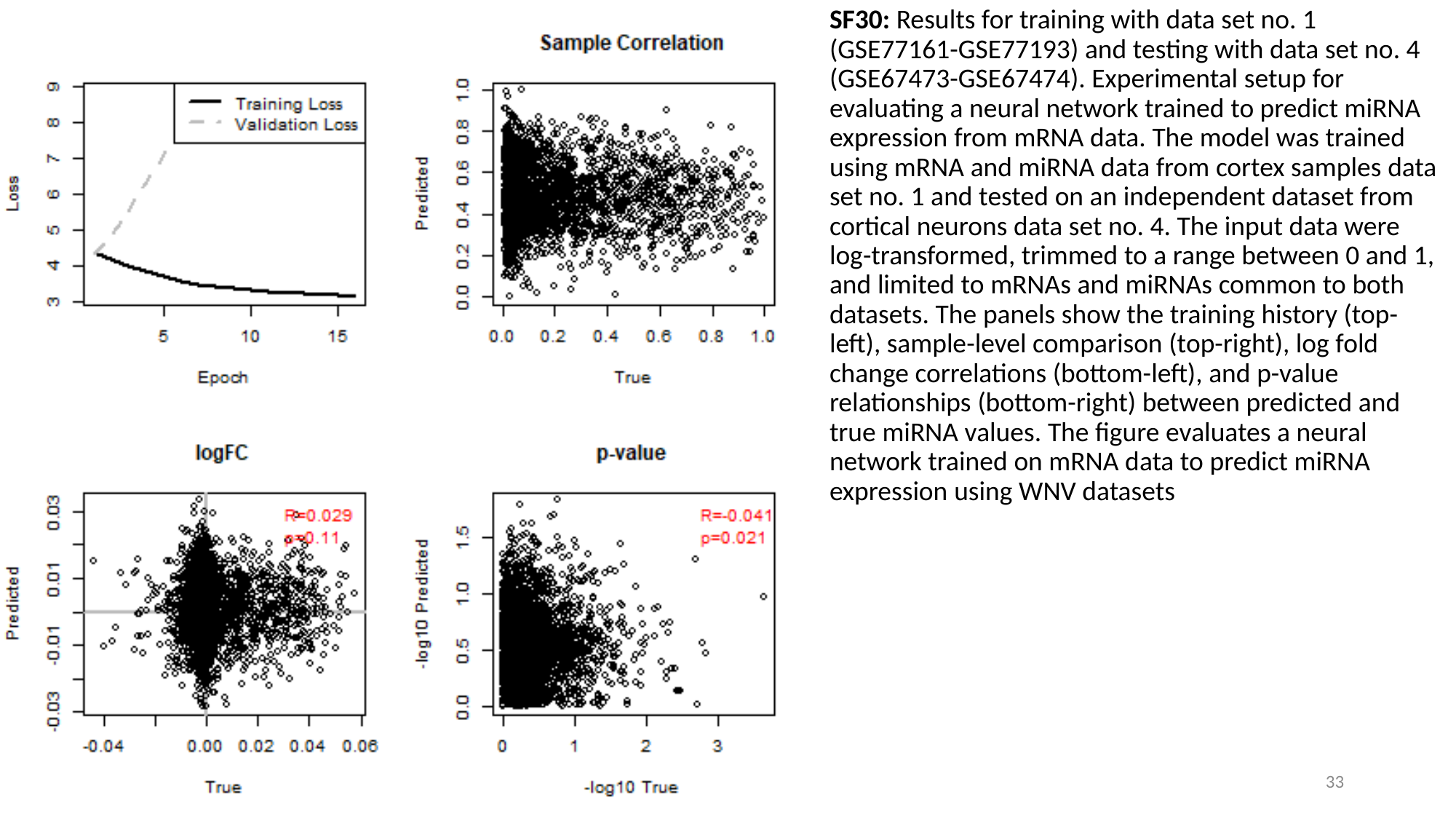

# SF30: Results for training with data set no. 1 (GSE77161-GSE77193) and testing with data set no. 4 (GSE67473-GSE67474). Experimental setup for evaluating a neural network trained to predict miRNA expression from mRNA data. The model was trained using mRNA and miRNA data from cortex samples data set no. 1 and tested on an independent dataset from cortical neurons data set no. 4. The input data were log-transformed, trimmed to a range between 0 and 1, and limited to mRNAs and miRNAs common to both datasets. The panels show the training history (top-left), sample-level comparison (top-right), log fold change correlations (bottom-left), and p-value relationships (bottom-right) between predicted and true miRNA values. The figure evaluates a neural network trained on mRNA data to predict miRNA expression using WNV datasets
33

## Slide 34
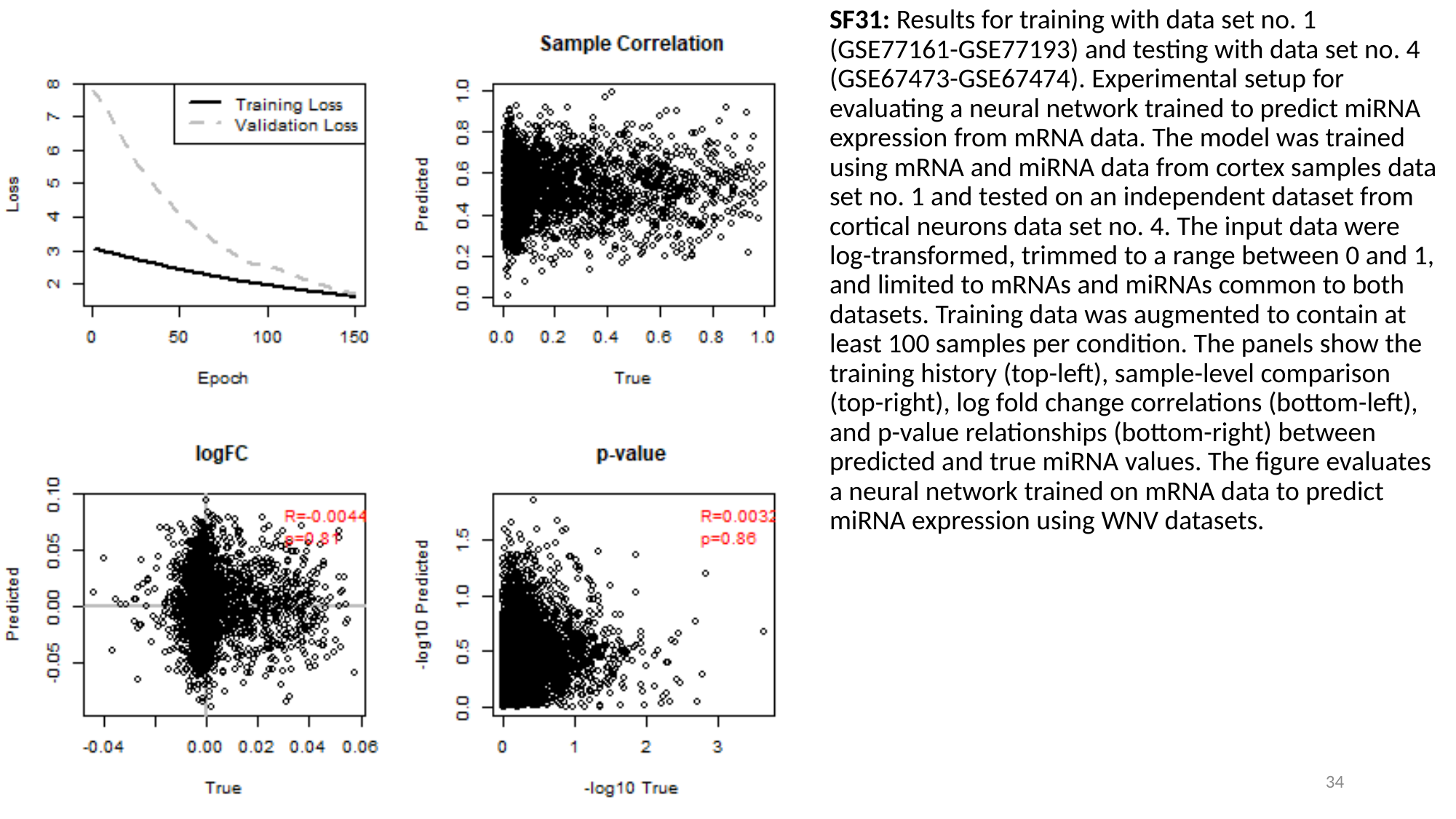

# SF31: Results for training with data set no. 1 (GSE77161-GSE77193) and testing with data set no. 4 (GSE67473-GSE67474). Experimental setup for evaluating a neural network trained to predict miRNA expression from mRNA data. The model was trained using mRNA and miRNA data from cortex samples data set no. 1 and tested on an independent dataset from cortical neurons data set no. 4. The input data were log-transformed, trimmed to a range between 0 and 1, and limited to mRNAs and miRNAs common to both datasets. Training data was augmented to contain at least 100 samples per condition. The panels show the training history (top-left), sample-level comparison (top-right), log fold change correlations (bottom-left), and p-value relationships (bottom-right) between predicted and true miRNA values. The figure evaluates a neural network trained on mRNA data to predict miRNA expression using WNV datasets.
34

## Slide 35
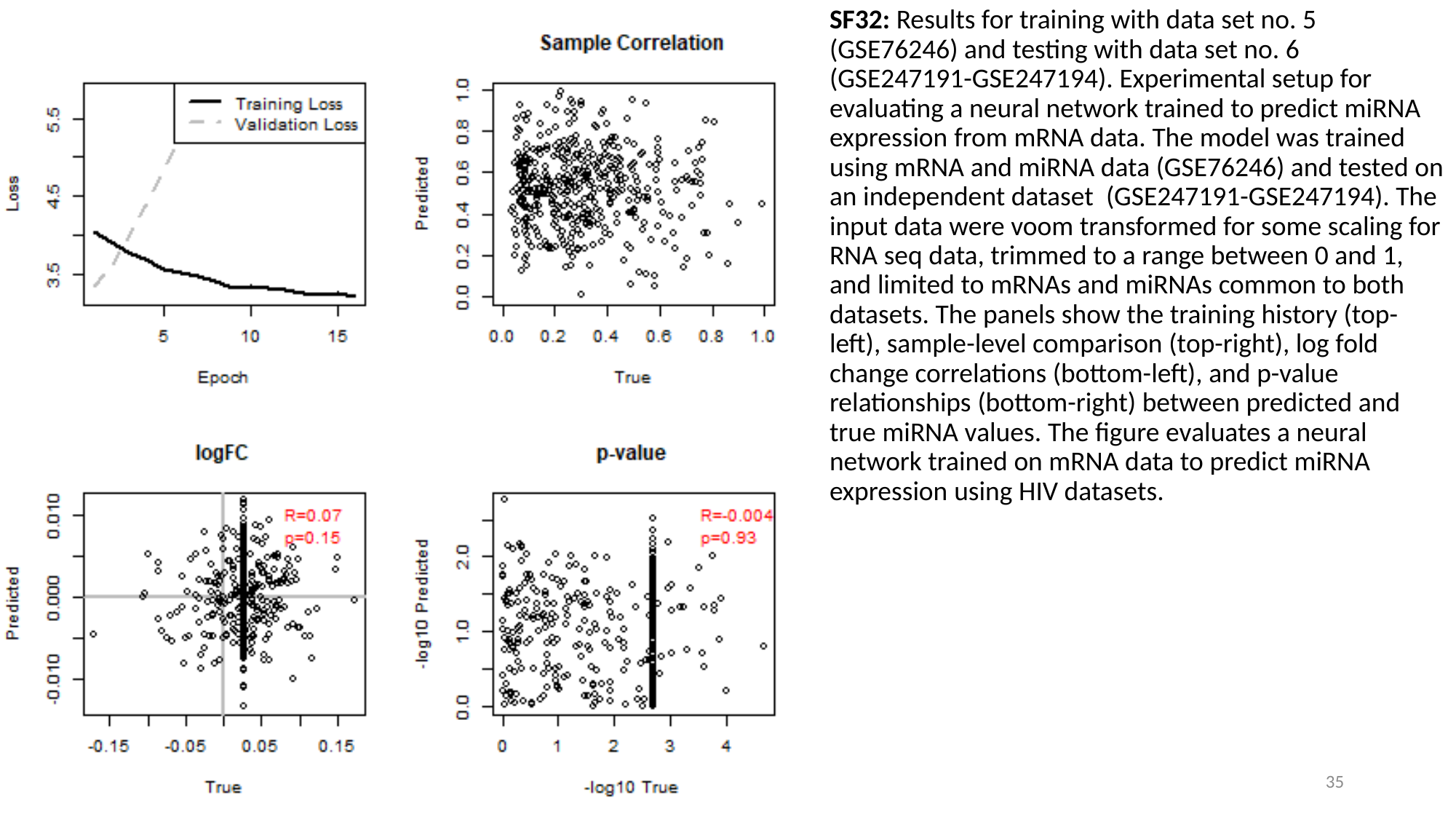

# SF32: Results for training with data set no. 5 (GSE76246) and testing with data set no. 6 (GSE247191-GSE247194). Experimental setup for evaluating a neural network trained to predict miRNA expression from mRNA data. The model was trained using mRNA and miRNA data (GSE76246) and tested on an independent dataset (GSE247191-GSE247194). The input data were voom transformed for some scaling for RNA seq data, trimmed to a range between 0 and 1, and limited to mRNAs and miRNAs common to both datasets. The panels show the training history (top-left), sample-level comparison (top-right), log fold change correlations (bottom-left), and p-value relationships (bottom-right) between predicted and true miRNA values. The figure evaluates a neural network trained on mRNA data to predict miRNA expression using HIV datasets.
35

## Slide 36
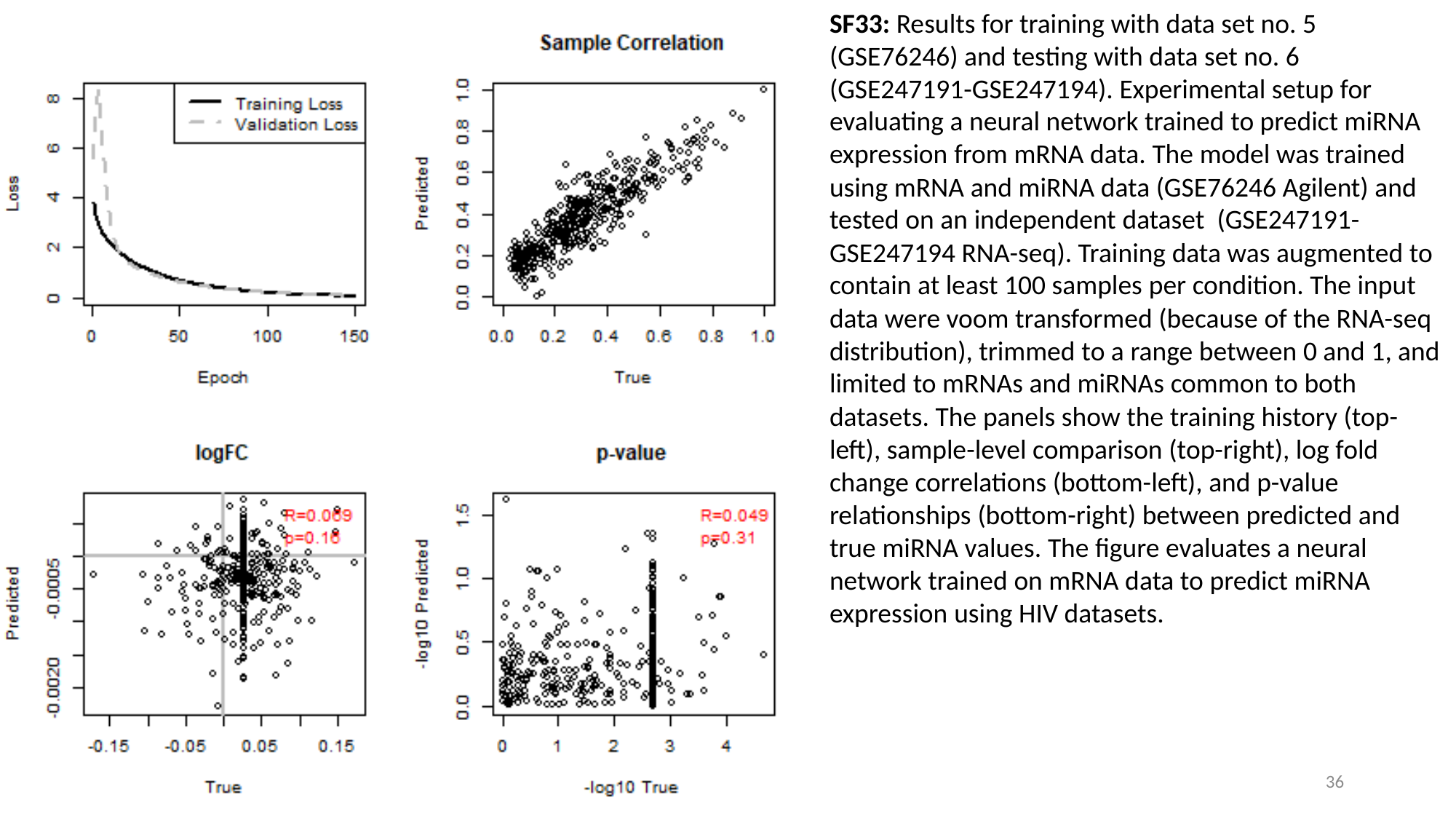

# SF33: Results for training with data set no. 5 (GSE76246) and testing with data set no. 6 (GSE247191-GSE247194). Experimental setup for evaluating a neural network trained to predict miRNA expression from mRNA data. The model was trained using mRNA and miRNA data (GSE76246 Agilent) and tested on an independent dataset (GSE247191-GSE247194 RNA-seq). Training data was augmented to contain at least 100 samples per condition. The input data were voom transformed (because of the RNA-seq distribution), trimmed to a range between 0 and 1, and limited to mRNAs and miRNAs common to both datasets. The panels show the training history (top-left), sample-level comparison (top-right), log fold change correlations (bottom-left), and p-value relationships (bottom-right) between predicted and true miRNA values. The figure evaluates a neural network trained on mRNA data to predict miRNA expression using HIV datasets.
36

## Slide 37
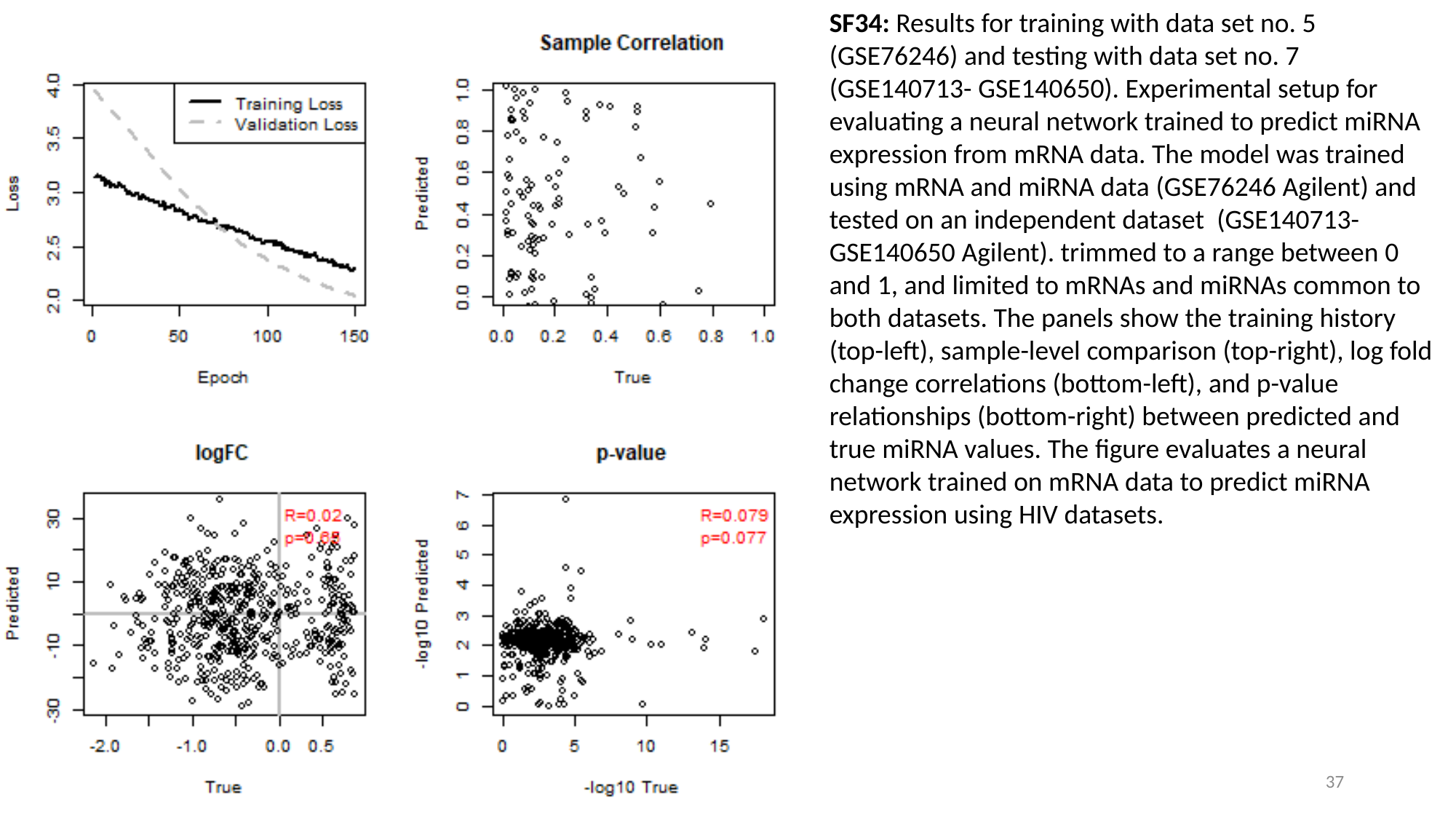

SF34: Results for training with data set no. 5 (GSE76246) and testing with data set no. 7 (GSE140713- GSE140650). Experimental setup for evaluating a neural network trained to predict miRNA expression from mRNA data. The model was trained using mRNA and miRNA data (GSE76246 Agilent) and tested on an independent dataset (GSE140713- GSE140650 Agilent). trimmed to a range between 0 and 1, and limited to mRNAs and miRNAs common to both datasets. The panels show the training history (top-left), sample-level comparison (top-right), log fold change correlations (bottom-left), and p-value relationships (bottom-right) between predicted and true miRNA values. The figure evaluates a neural network trained on mRNA data to predict miRNA expression using HIV datasets.
37

## Slide 38
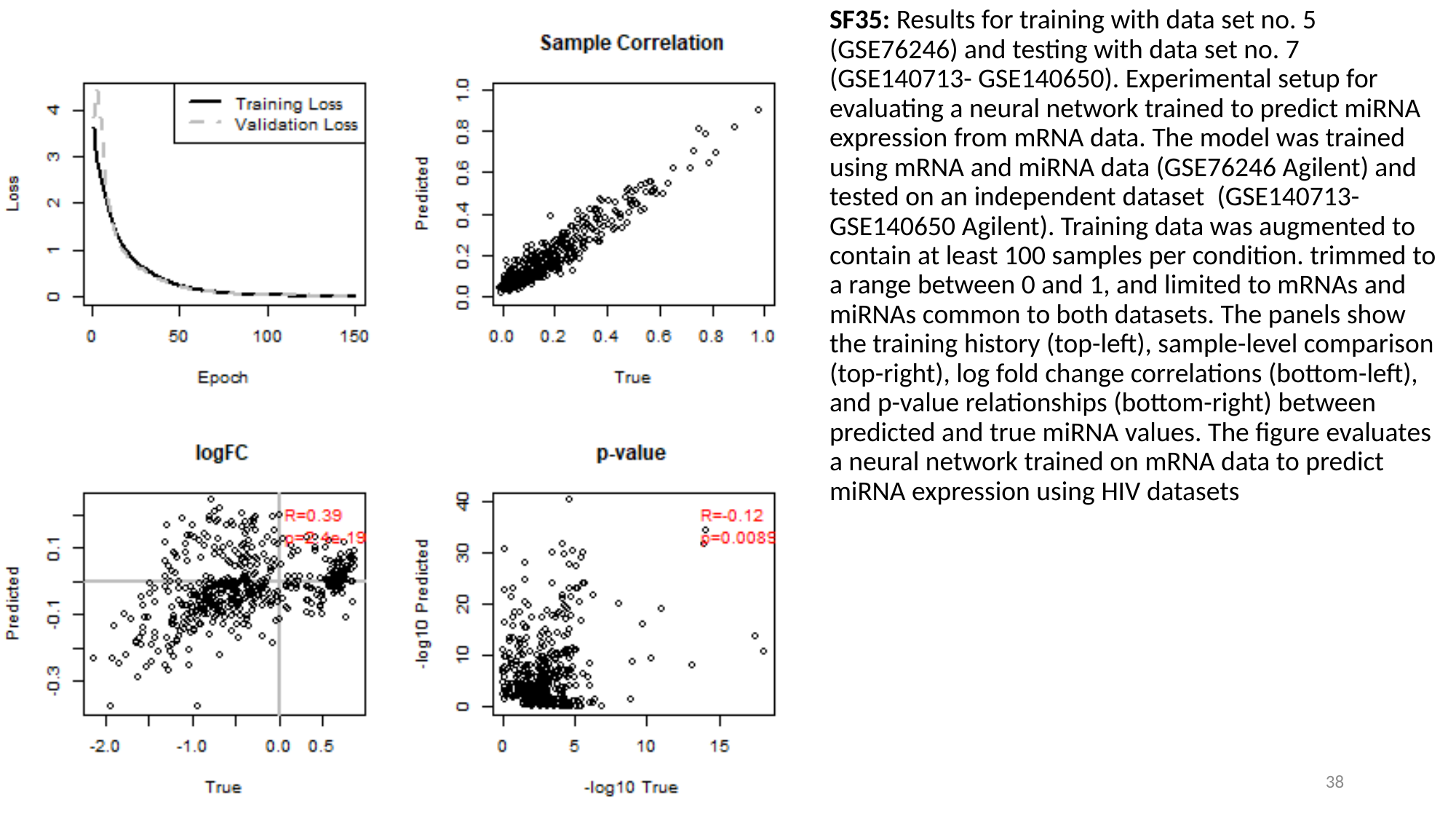

# SF35: Results for training with data set no. 5 (GSE76246) and testing with data set no. 7 (GSE140713- GSE140650). Experimental setup for evaluating a neural network trained to predict miRNA expression from mRNA data. The model was trained using mRNA and miRNA data (GSE76246 Agilent) and tested on an independent dataset (GSE140713-GSE140650 Agilent). Training data was augmented to contain at least 100 samples per condition. trimmed to a range between 0 and 1, and limited to mRNAs and miRNAs common to both datasets. The panels show the training history (top-left), sample-level comparison (top-right), log fold change correlations (bottom-left), and p-value relationships (bottom-right) between predicted and true miRNA values. The figure evaluates a neural network trained on mRNA data to predict miRNA expression using HIV datasets
38

## Slide 39
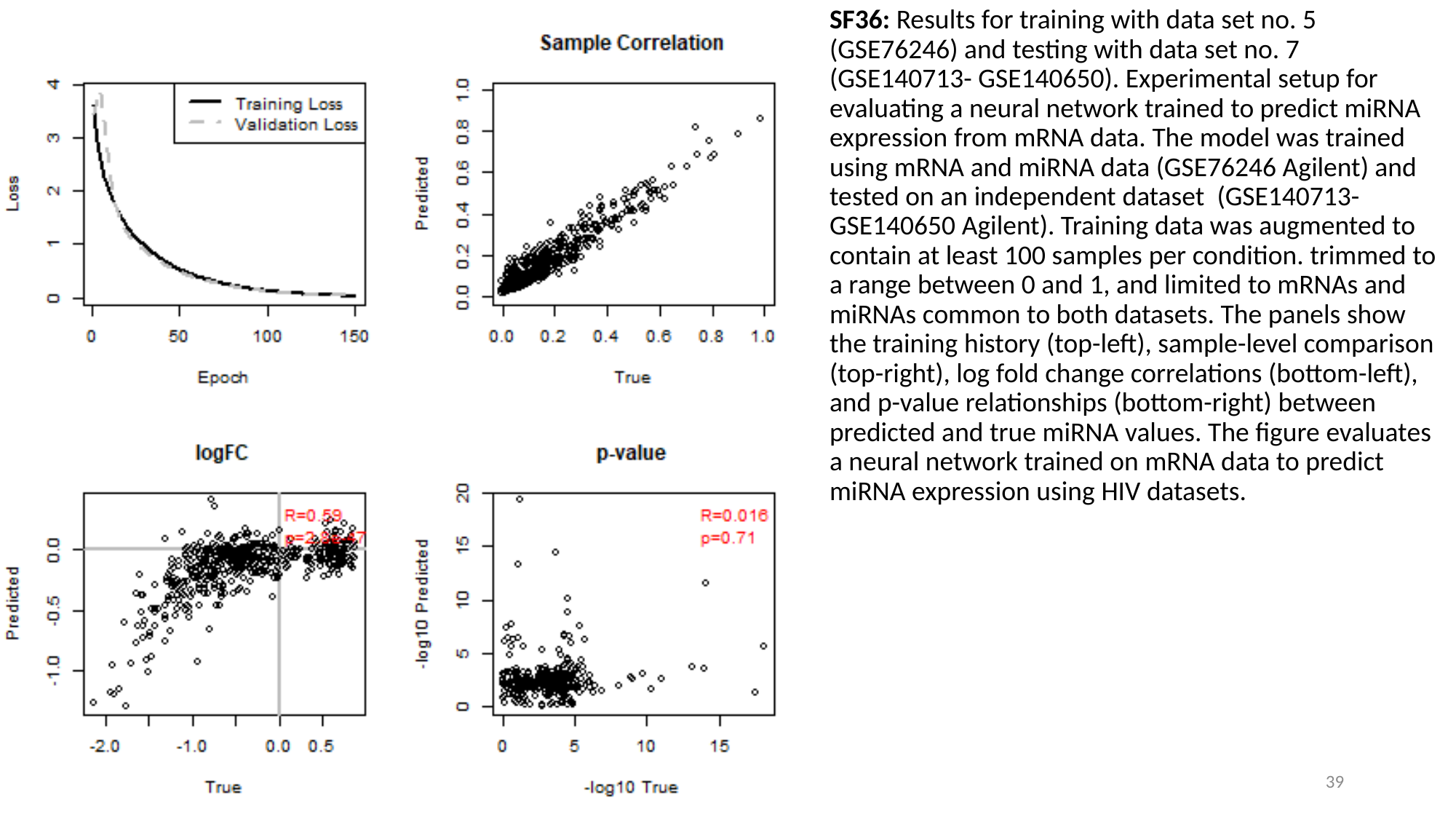

SF36: Results for training with data set no. 5 (GSE76246) and testing with data set no. 7 (GSE140713- GSE140650). Experimental setup for evaluating a neural network trained to predict miRNA expression from mRNA data. The model was trained using mRNA and miRNA data (GSE76246 Agilent) and tested on an independent dataset (GSE140713-GSE140650 Agilent). Training data was augmented to contain at least 100 samples per condition. trimmed to a range between 0 and 1, and limited to mRNAs and miRNAs common to both datasets. The panels show the training history (top-left), sample-level comparison (top-right), log fold change correlations (bottom-left), and p-value relationships (bottom-right) between predicted and true miRNA values. The figure evaluates a neural network trained on mRNA data to predict miRNA expression using HIV datasets.
39

## Slide 40
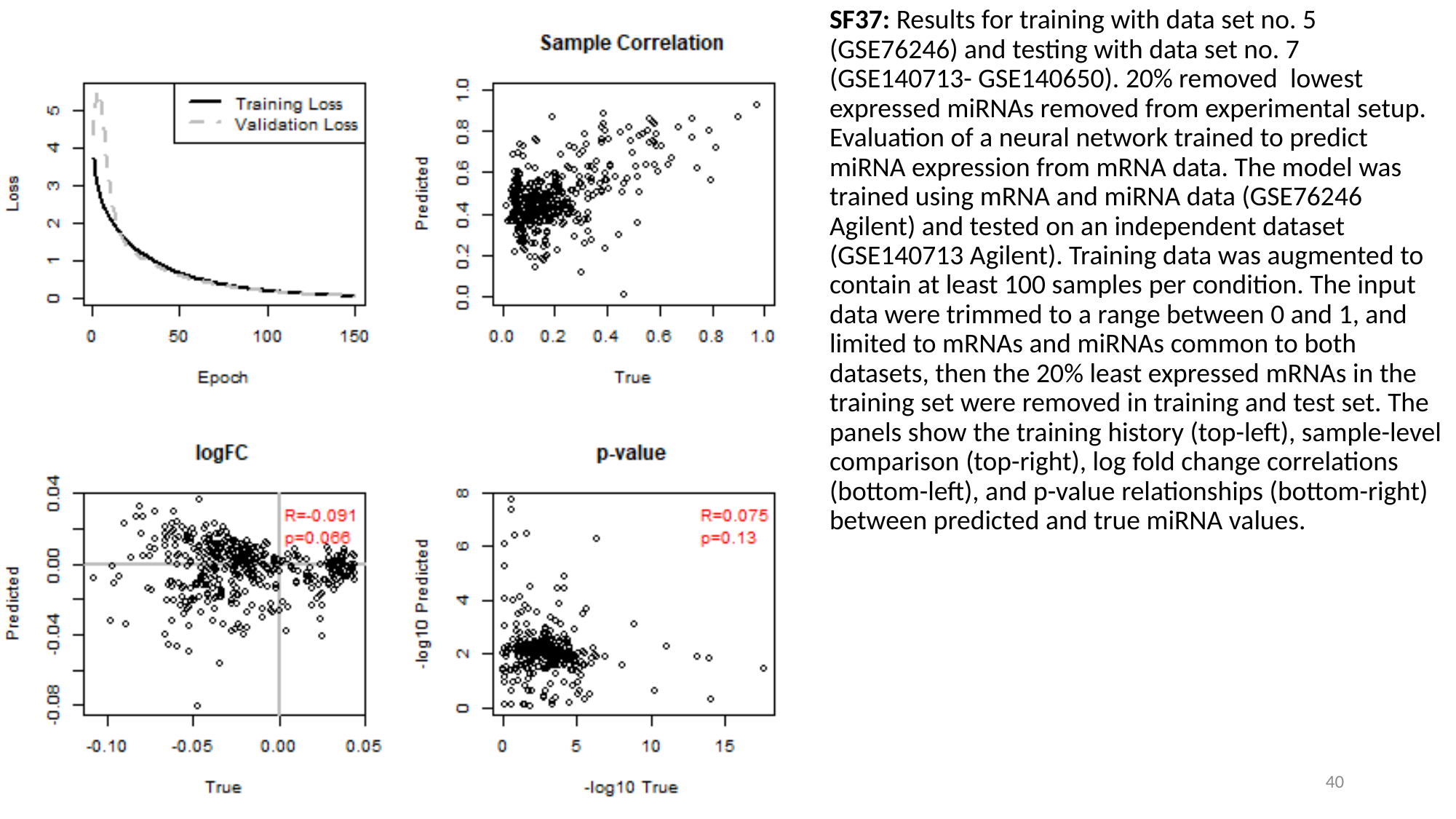

# SF37: Results for training with data set no. 5 (GSE76246) and testing with data set no. 7 (GSE140713- GSE140650). 20% removed lowest expressed miRNAs removed from experimental setup. Evaluation of a neural network trained to predict miRNA expression from mRNA data. The model was trained using mRNA and miRNA data (GSE76246 Agilent) and tested on an independent dataset (GSE140713 Agilent). Training data was augmented to contain at least 100 samples per condition. The input data were trimmed to a range between 0 and 1, and limited to mRNAs and miRNAs common to both datasets, then the 20% least expressed mRNAs in the training set were removed in training and test set. The panels show the training history (top-left), sample-level comparison (top-right), log fold change correlations (bottom-left), and p-value relationships (bottom-right) between predicted and true miRNA values.
40

## Slide 41
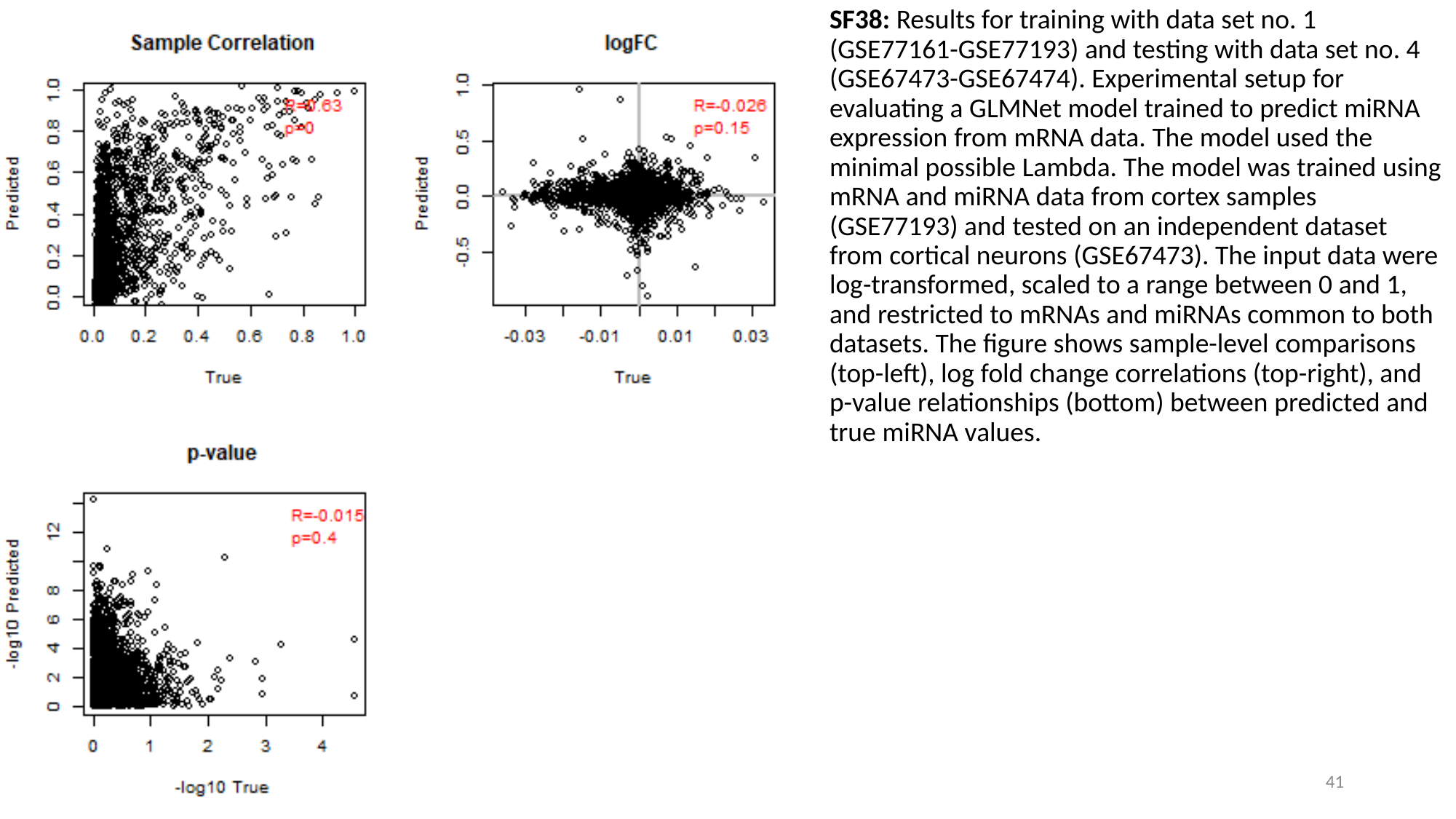

# SF38: Results for training with data set no. 1 (GSE77161-GSE77193) and testing with data set no. 4 (GSE67473-GSE67474). Experimental setup for evaluating a GLMNet model trained to predict miRNA expression from mRNA data. The model used the minimal possible Lambda. The model was trained using mRNA and miRNA data from cortex samples (GSE77193) and tested on an independent dataset from cortical neurons (GSE67473). The input data were log-transformed, scaled to a range between 0 and 1, and restricted to mRNAs and miRNAs common to both datasets. The figure shows sample-level comparisons (top-left), log fold change correlations (top-right), and p-value relationships (bottom) between predicted and true miRNA values.
41

## Slide 42
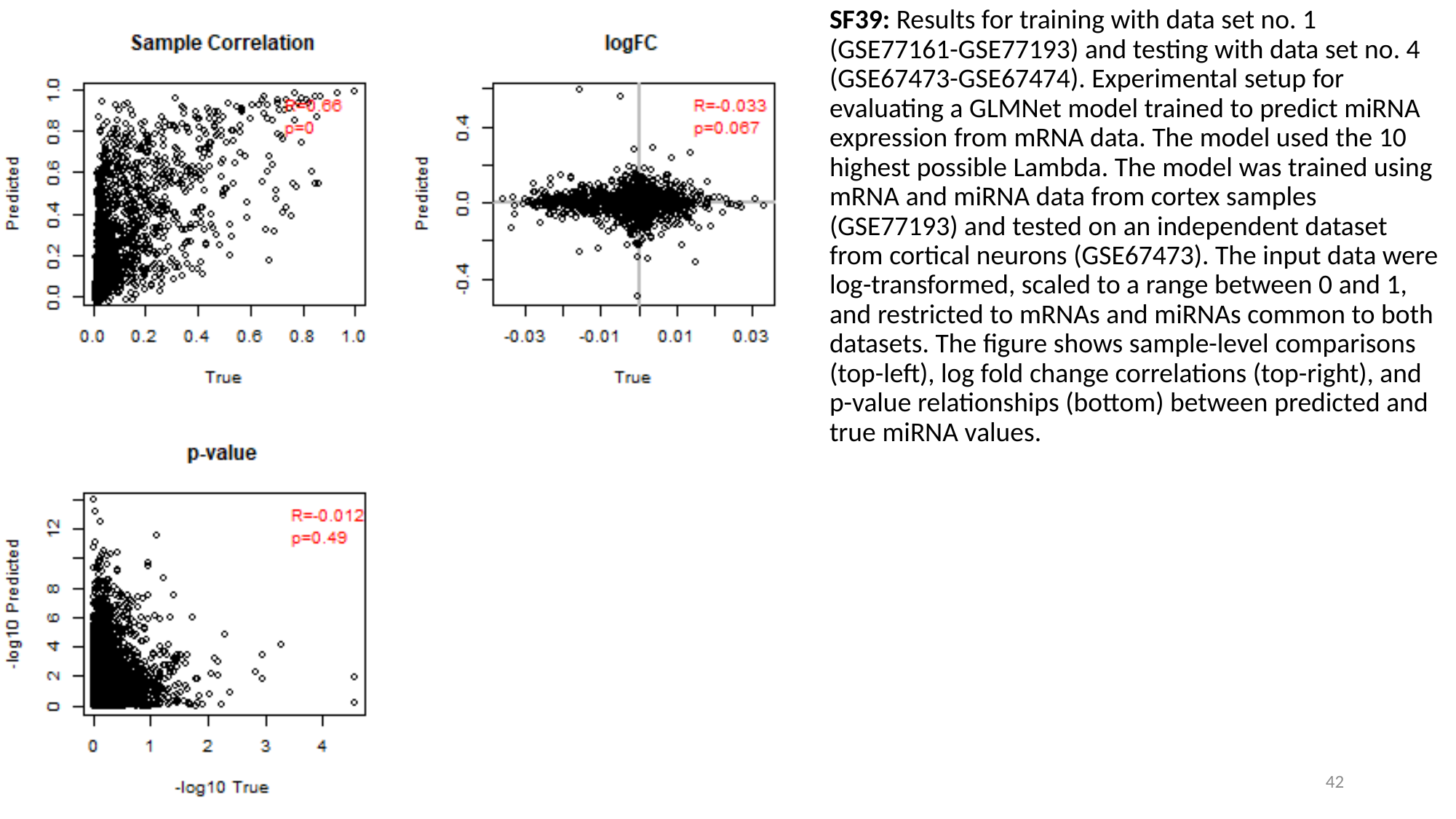

# SF39: Results for training with data set no. 1 (GSE77161-GSE77193) and testing with data set no. 4 (GSE67473-GSE67474). Experimental setup for evaluating a GLMNet model trained to predict miRNA expression from mRNA data. The model used the 10 highest possible Lambda. The model was trained using mRNA and miRNA data from cortex samples (GSE77193) and tested on an independent dataset from cortical neurons (GSE67473). The input data were log-transformed, scaled to a range between 0 and 1, and restricted to mRNAs and miRNAs common to both datasets. The figure shows sample-level comparisons (top-left), log fold change correlations (top-right), and p-value relationships (bottom) between predicted and true miRNA values.
42

## Slide 43
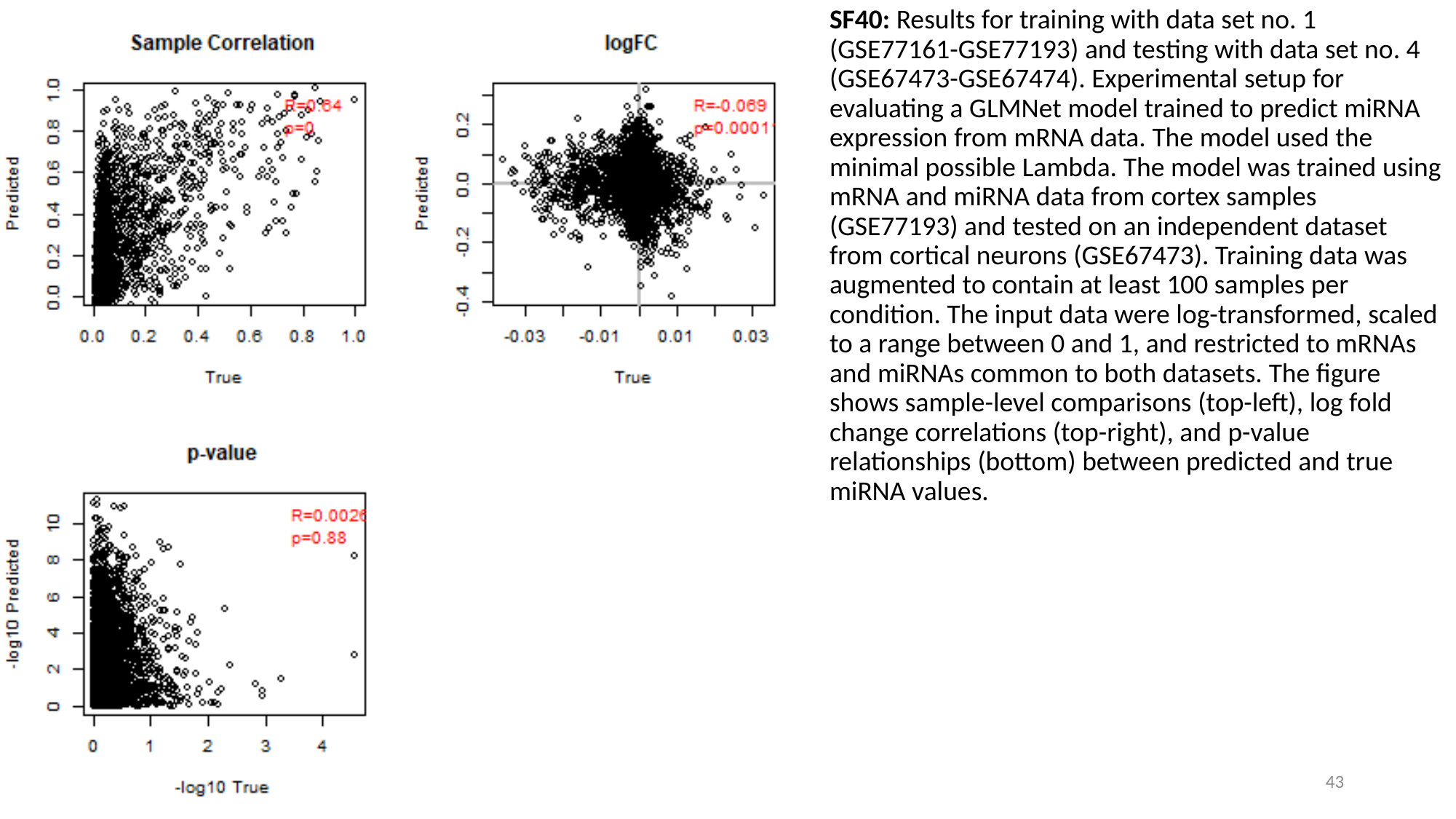

# SF40: Results for training with data set no. 1 (GSE77161-GSE77193) and testing with data set no. 4 (GSE67473-GSE67474). Experimental setup for evaluating a GLMNet model trained to predict miRNA expression from mRNA data. The model used the minimal possible Lambda. The model was trained using mRNA and miRNA data from cortex samples (GSE77193) and tested on an independent dataset from cortical neurons (GSE67473). Training data was augmented to contain at least 100 samples per condition. The input data were log-transformed, scaled to a range between 0 and 1, and restricted to mRNAs and miRNAs common to both datasets. The figure shows sample-level comparisons (top-left), log fold change correlations (top-right), and p-value relationships (bottom) between predicted and true miRNA values.
43

## Slide 44
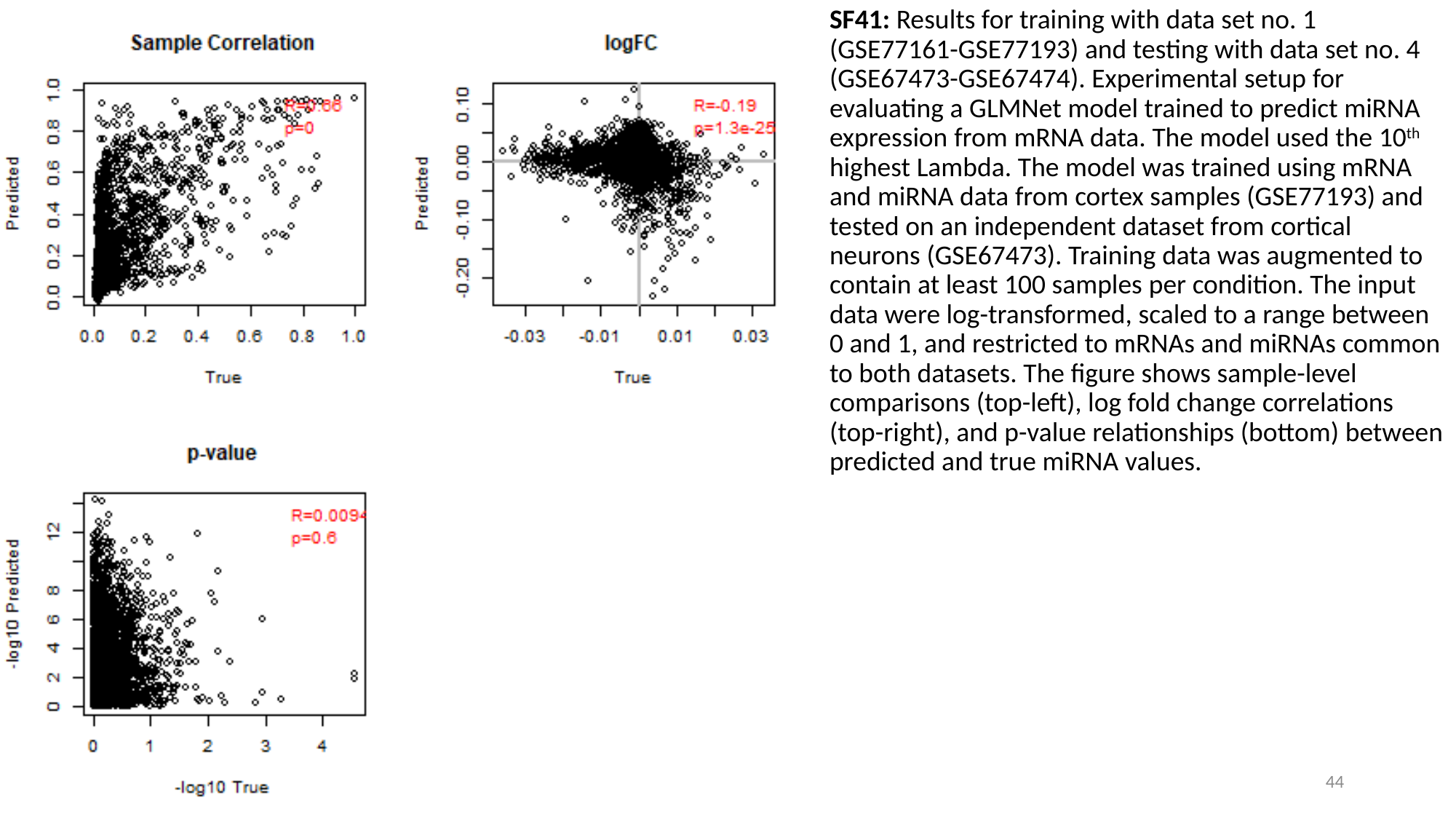

# SF41: Results for training with data set no. 1 (GSE77161-GSE77193) and testing with data set no. 4 (GSE67473-GSE67474). Experimental setup for evaluating a GLMNet model trained to predict miRNA expression from mRNA data. The model used the 10th highest Lambda. The model was trained using mRNA and miRNA data from cortex samples (GSE77193) and tested on an independent dataset from cortical neurons (GSE67473). Training data was augmented to contain at least 100 samples per condition. The input data were log-transformed, scaled to a range between 0 and 1, and restricted to mRNAs and miRNAs common to both datasets. The figure shows sample-level comparisons (top-left), log fold change correlations (top-right), and p-value relationships (bottom) between predicted and true miRNA values.
44

## Slide 45
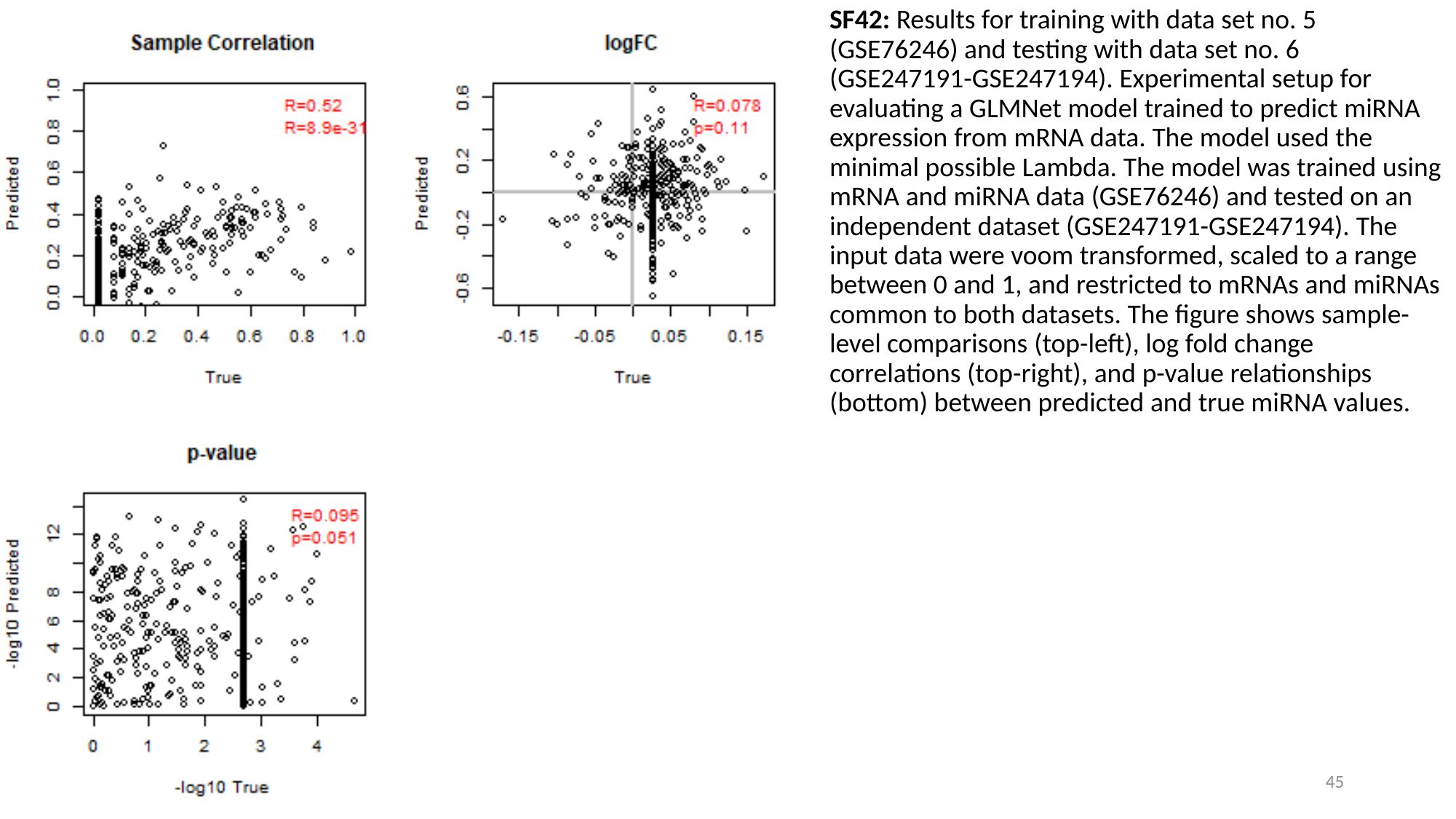

# SF42: Results for training with data set no. 5 (GSE76246) and testing with data set no. 6 (GSE247191-GSE247194). Experimental setup for evaluating a GLMNet model trained to predict miRNA expression from mRNA data. The model used the minimal possible Lambda. The model was trained using mRNA and miRNA data (GSE76246) and tested on an independent dataset (GSE247191-GSE247194). The input data were voom transformed, scaled to a range between 0 and 1, and restricted to mRNAs and miRNAs common to both datasets. The figure shows sample-level comparisons (top-left), log fold change correlations (top-right), and p-value relationships (bottom) between predicted and true miRNA values.
45

## Slide 46
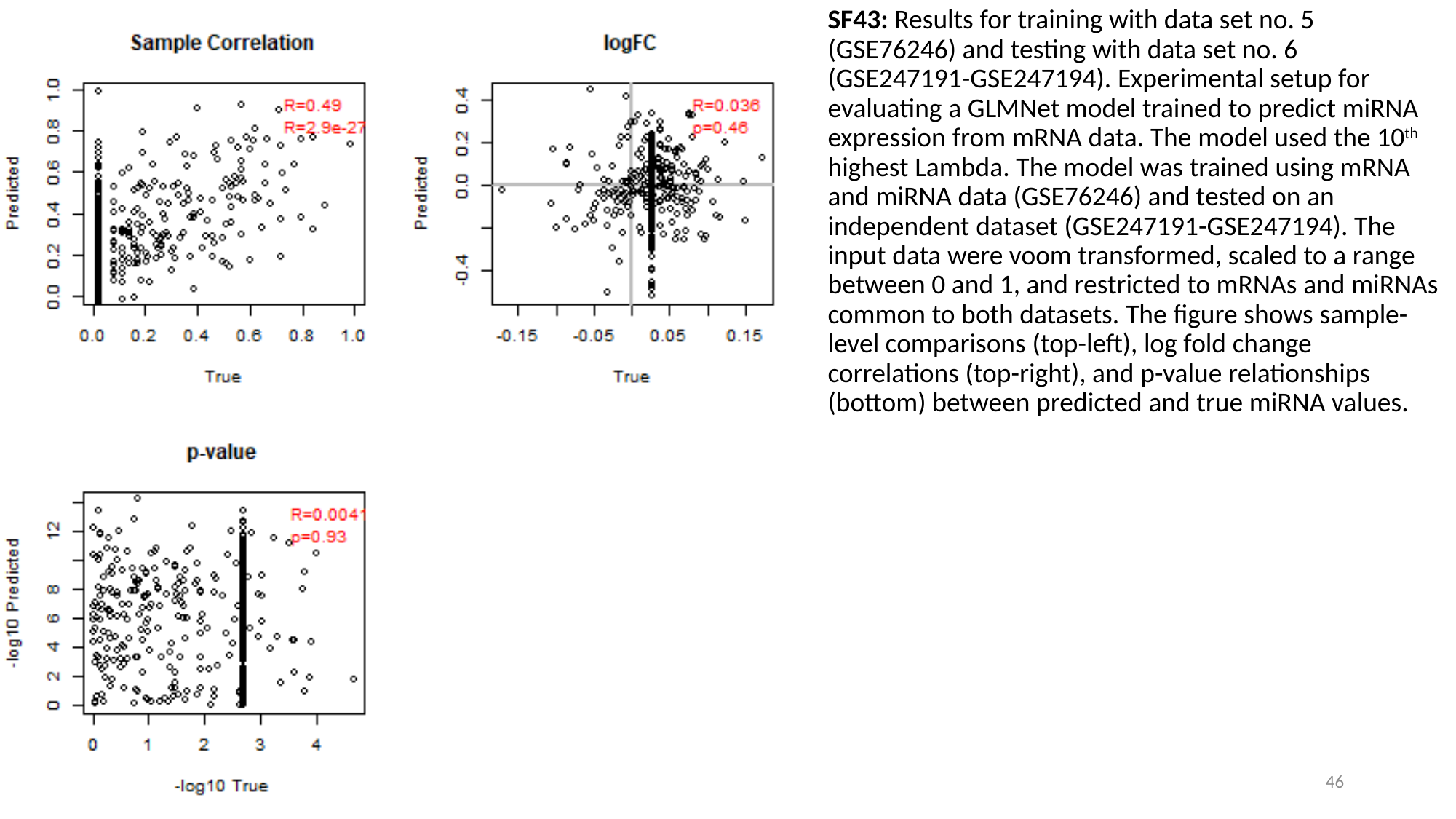

# SF43: Results for training with data set no. 5 (GSE76246) and testing with data set no. 6 (GSE247191-GSE247194). Experimental setup for evaluating a GLMNet model trained to predict miRNA expression from mRNA data. The model used the 10th highest Lambda. The model was trained using mRNA and miRNA data (GSE76246) and tested on an independent dataset (GSE247191-GSE247194). The input data were voom transformed, scaled to a range between 0 and 1, and restricted to mRNAs and miRNAs common to both datasets. The figure shows sample-level comparisons (top-left), log fold change correlations (top-right), and p-value relationships (bottom) between predicted and true miRNA values.
46

## Slide 47
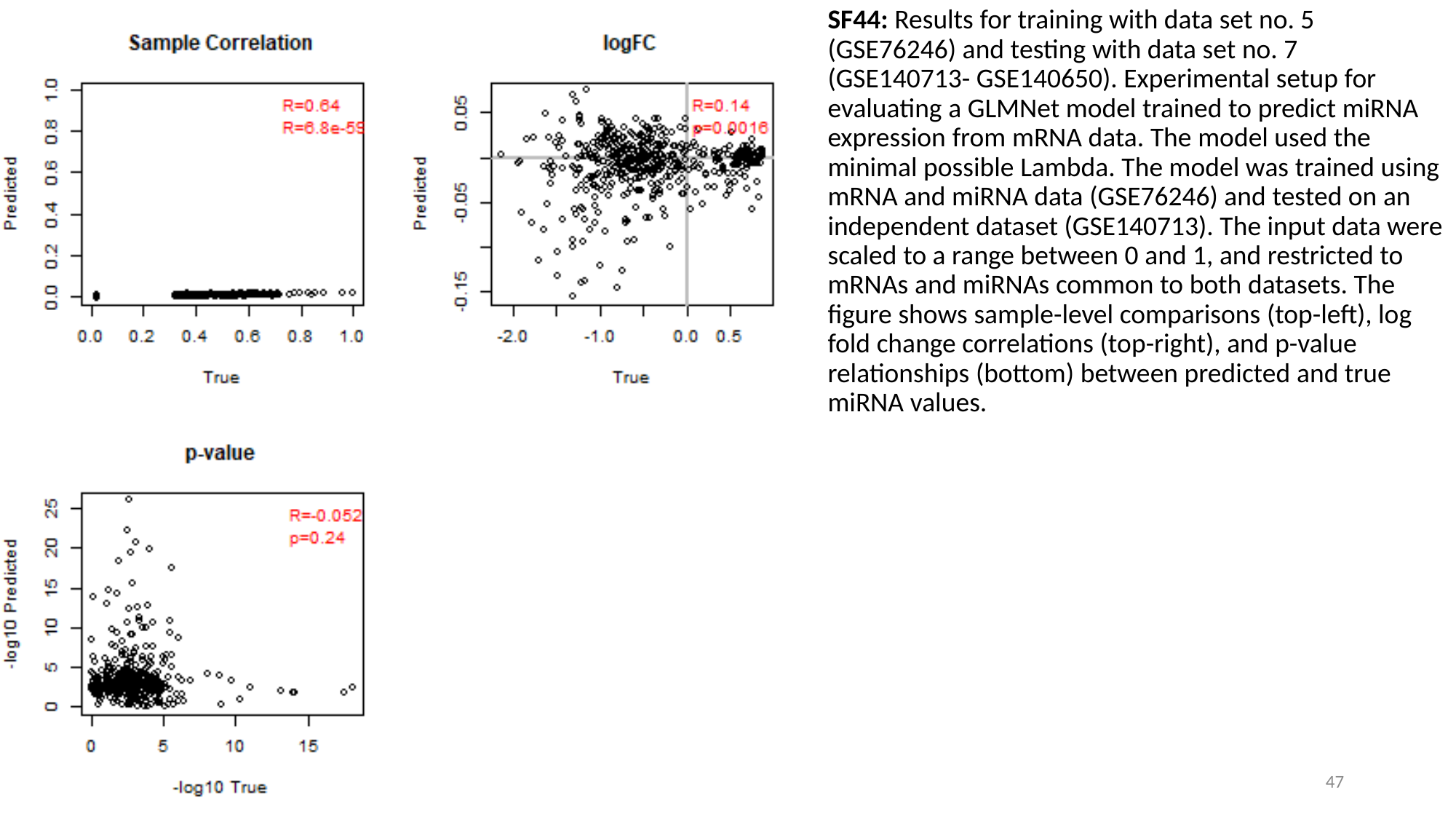

# SF44: Results for training with data set no. 5 (GSE76246) and testing with data set no. 7 (GSE140713- GSE140650). Experimental setup for evaluating a GLMNet model trained to predict miRNA expression from mRNA data. The model used the minimal possible Lambda. The model was trained using mRNA and miRNA data (GSE76246) and tested on an independent dataset (GSE140713). The input data were scaled to a range between 0 and 1, and restricted to mRNAs and miRNAs common to both datasets. The figure shows sample-level comparisons (top-left), log fold change correlations (top-right), and p-value relationships (bottom) between predicted and true miRNA values.
47

## Slide 48
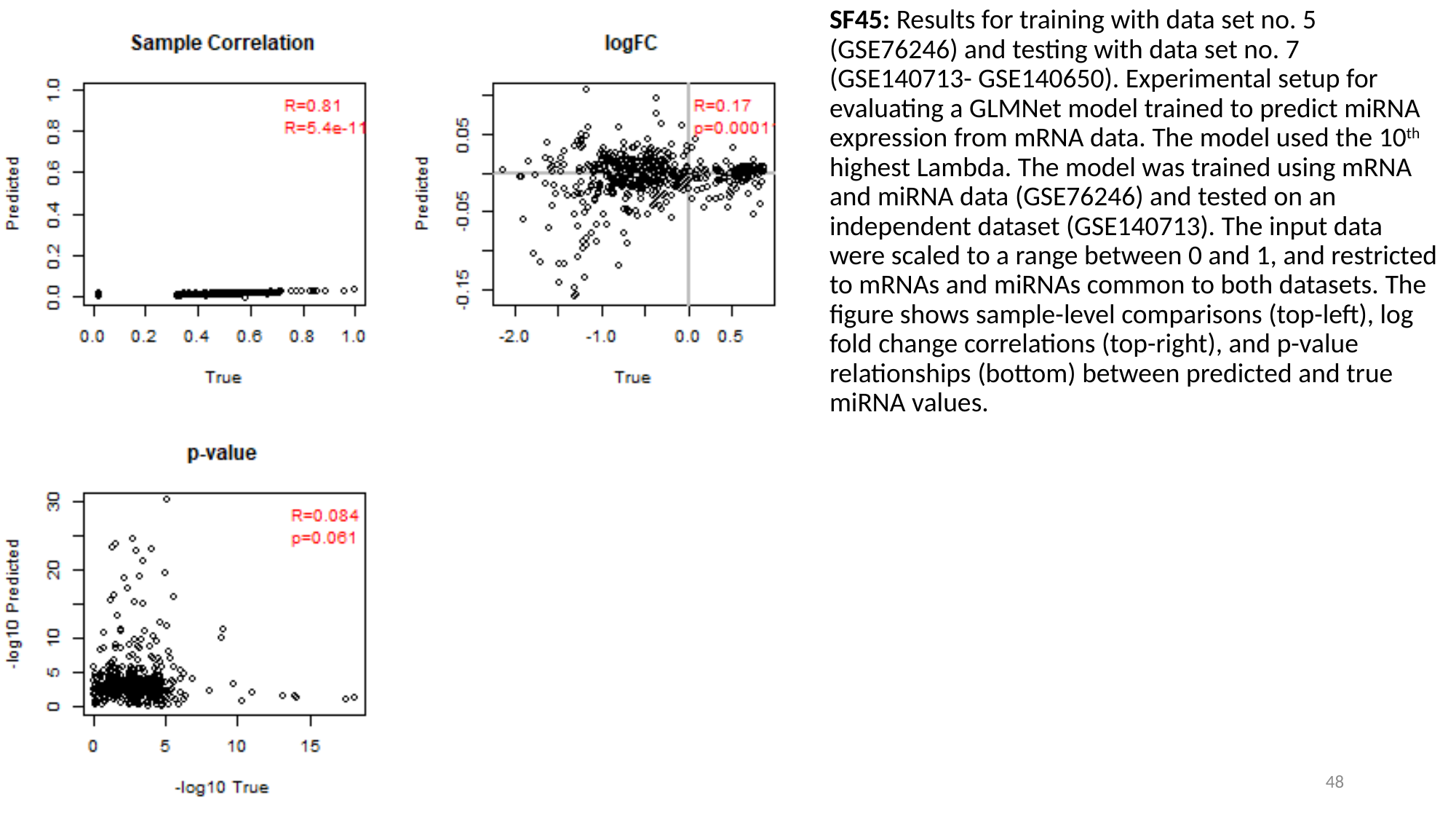

# SF45: Results for training with data set no. 5 (GSE76246) and testing with data set no. 7 (GSE140713- GSE140650). Experimental setup for evaluating a GLMNet model trained to predict miRNA expression from mRNA data. The model used the 10th highest Lambda. The model was trained using mRNA and miRNA data (GSE76246) and tested on an independent dataset (GSE140713). The input data were scaled to a range between 0 and 1, and restricted to mRNAs and miRNAs common to both datasets. The figure shows sample-level comparisons (top-left), log fold change correlations (top-right), and p-value relationships (bottom) between predicted and true miRNA values.
48

## Slide 49
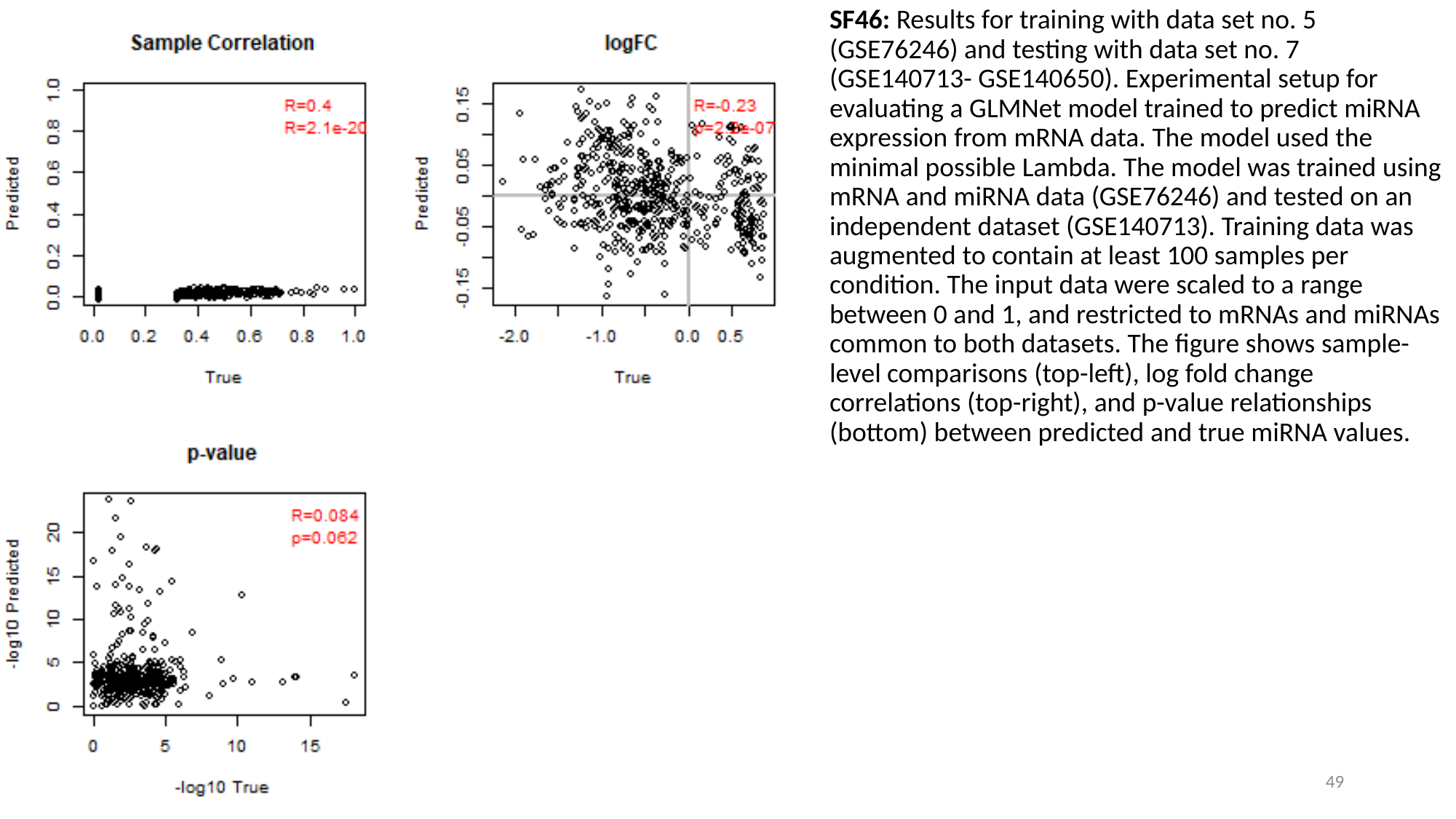

# SF46: Results for training with data set no. 5 (GSE76246) and testing with data set no. 7 (GSE140713- GSE140650). Experimental setup for evaluating a GLMNet model trained to predict miRNA expression from mRNA data. The model used the minimal possible Lambda. The model was trained using mRNA and miRNA data (GSE76246) and tested on an independent dataset (GSE140713). Training data was augmented to contain at least 100 samples per condition. The input data were scaled to a range between 0 and 1, and restricted to mRNAs and miRNAs common to both datasets. The figure shows sample-level comparisons (top-left), log fold change correlations (top-right), and p-value relationships (bottom) between predicted and true miRNA values.
49

## Slide 50
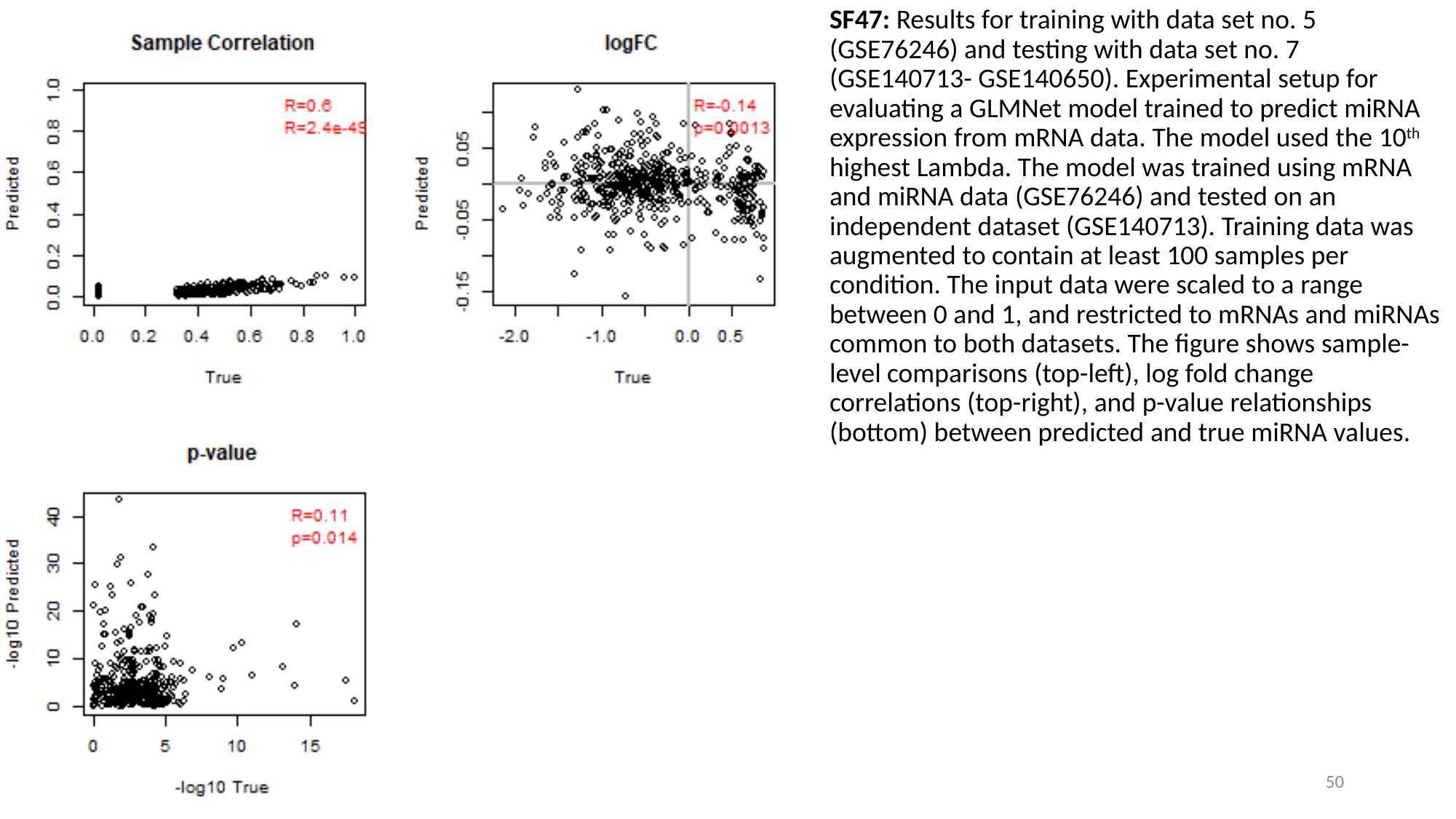

# SF47: Results for training with data set no. 5 (GSE76246) and testing with data set no. 7 (GSE140713- GSE140650). Experimental setup for evaluating a GLMNet model trained to predict miRNA expression from mRNA data. The model used the 10th highest Lambda. The model was trained using mRNA and miRNA data (GSE76246) and tested on an independent dataset (GSE140713). Training data was augmented to contain at least 100 samples per condition. The input data were scaled to a range between 0 and 1, and restricted to mRNAs and miRNAs common to both datasets. The figure shows sample-level comparisons (top-left), log fold change correlations (top-right), and p-value relationships (bottom) between predicted and true miRNA values.
50

## Slide 51
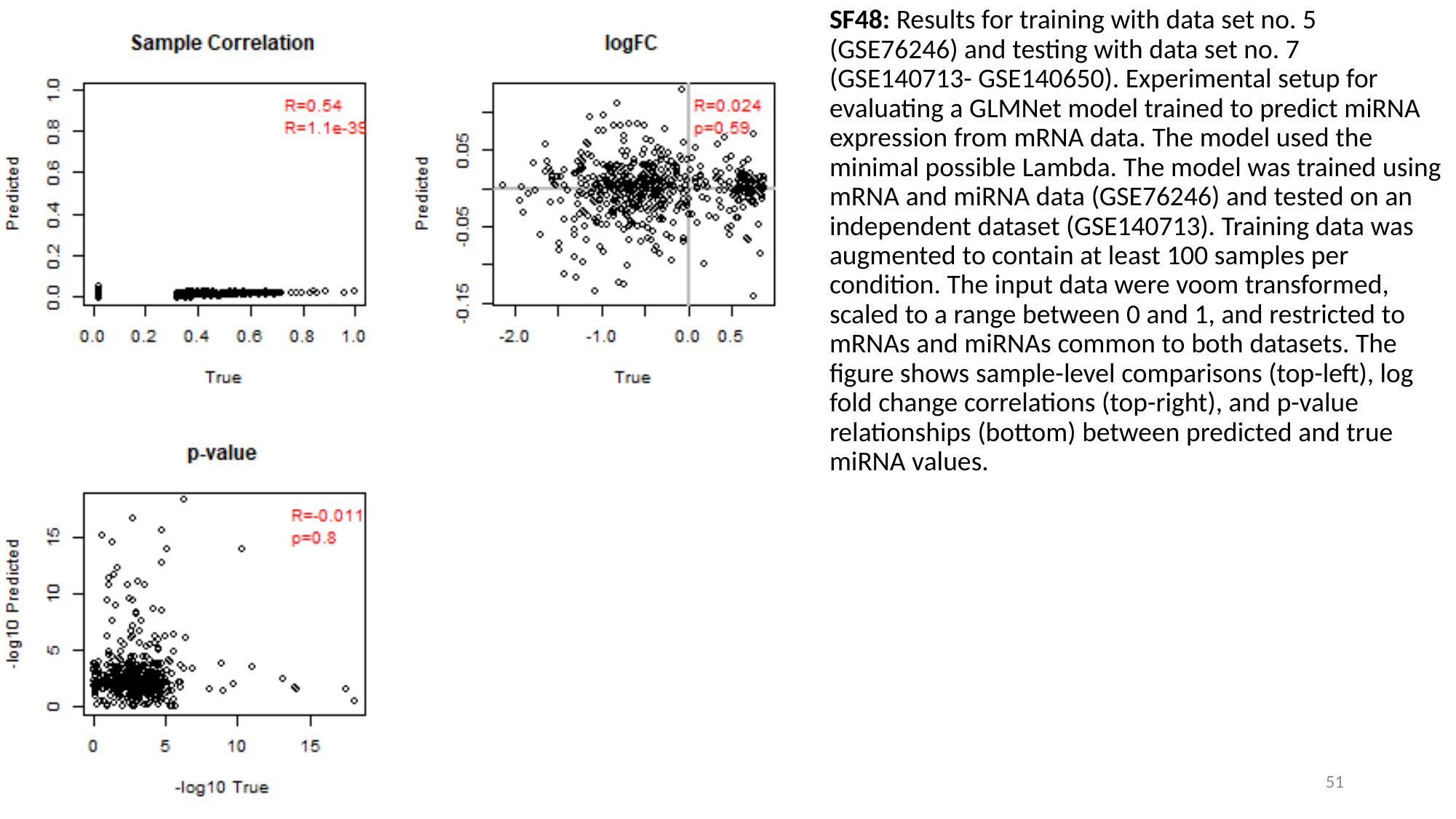

# SF48: Results for training with data set no. 5 (GSE76246) and testing with data set no. 7 (GSE140713- GSE140650). Experimental setup for evaluating a GLMNet model trained to predict miRNA expression from mRNA data. The model used the minimal possible Lambda. The model was trained using mRNA and miRNA data (GSE76246) and tested on an independent dataset (GSE140713). Training data was augmented to contain at least 100 samples per condition. The input data were voom transformed, scaled to a range between 0 and 1, and restricted to mRNAs and miRNAs common to both datasets. The figure shows sample-level comparisons (top-left), log fold change correlations (top-right), and p-value relationships (bottom) between predicted and true miRNA values.
51

## Slide 52
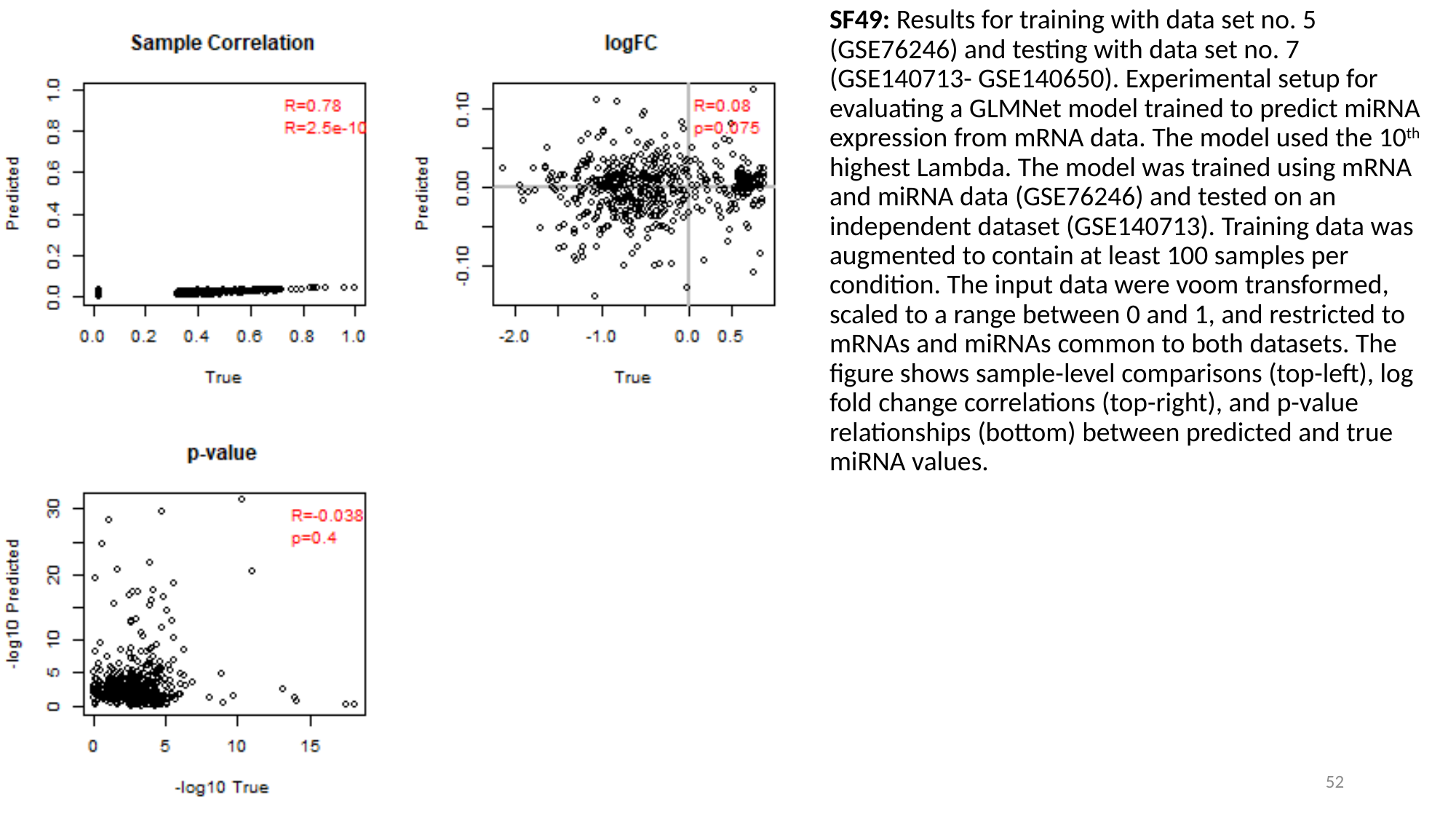

# SF49: Results for training with data set no. 5 (GSE76246) and testing with data set no. 7 (GSE140713- GSE140650). Experimental setup for evaluating a GLMNet model trained to predict miRNA expression from mRNA data. The model used the 10th highest Lambda. The model was trained using mRNA and miRNA data (GSE76246) and tested on an independent dataset (GSE140713). Training data was augmented to contain at least 100 samples per condition. The input data were voom transformed, scaled to a range between 0 and 1, and restricted to mRNAs and miRNAs common to both datasets. The figure shows sample-level comparisons (top-left), log fold change correlations (top-right), and p-value relationships (bottom) between predicted and true miRNA values.
52
